# Bidirectional cross metathesis and ring-closing metathesis/ring opening of a *C*_2_-symmetric building block: a strategy for the synthesis of decanolide natural products

**DOI:** 10.3762/bjoc.9.289

**Published:** 2013-11-18

**Authors:** Bernd Schmidt, Oliver Kunz

**Affiliations:** 1 Institut für Chemie, Organische Synthesechemie, Universität Potsdam, Karl-Liebknecht-Straße 24-25, 14476 Potsdam-Golm, Germany

**Keywords:** dienes, enzyme catalysis, lactones, metathesis, natural products, ruthenium

## Abstract

Starting from the conveniently available ex-chiral pool building block (*R*,*R*)-hexa-1,5-diene-3,4-diol, the ten-membered ring lactones stagonolide E and curvulide A were synthesized using a bidirectional olefin-metathesis functionalization of the terminal double bonds. Key steps are (i) a site-selective cross metathesis, (ii) a highly diastereoselective extended tethered RCM to furnish a (*Z*,*E*)-configured dienyl carboxylic acid and (iii) a Ru–lipase-catalyzed dynamic kinetic resolution to establish the desired configuration at C9. Ring closure was accomplished by macrolactonization. Curvulide A was synthesized from stagonolide E through Sharpless epoxidation.

## Introduction

Bidirectional synthesis through termini differentiation of *meso*, *C*_2_-symmetric or unsymmetric building blocks has emerged as an important strategy in natural product synthesis over the past two decades [1]. Early on, enantiomerically pure *C*_2_-symmetric compounds were identified as particularly useful starting materials, because their termini are homotopic. Therefore, desymmetrization can be accomplished by monofunctionalization, making elaborate reagent or catalyst-controlled transformations unnecessary [2]. In this regard, (*R*,*R*)-hexa-1,5-diene-3,4-diol (**1**) [3–6] and its enantiomer *ent*-**1** [7] have emerged as highly valuable starting points for target molecule syntheses which rely on dual olefin metathesis reactions. The two metathesis transformations may either be two identical CM [8–9] or RCM steps [10], yielding *C*_2_-symmetric products in which the newly formed double bonds remain homotopic, or two different CM or RCM steps, or a combination of one CM and one RCM transformation. The latter cases lead to *C*_1_-symmetric products and hence a differentiation of the C–C double bonds generated through metathesis. Examples for the utilization of these approaches in the synthesis of target molecules from **1** or *ent*-**1** include sialic acids [11], cladospolide C [12], iriomoteolide 3a [13–14], thromboxane B2 [15], didemniserinolipid B [16], squamostolide [17], muricatacine [18], rollicosin [19], phomopsolide C [7] and both enantiomers of seimatopolide A [20].

**Scheme 1:**
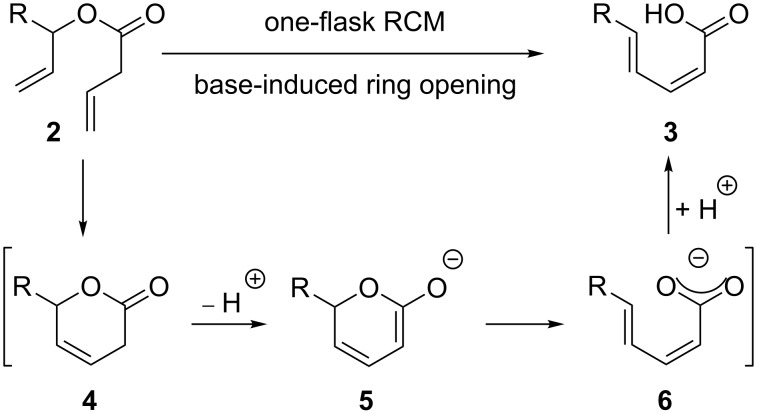
RCM/base-induced ring-opening sequence.

Over the past few years the development and application of one-flask sequences comprising at least one metathesis step has attracted increasing attention [21–23]. Such sequences provide rapid access to constitutional isomers or functionalized derivatives of the actual metathesis products in just one step. An example recently published by us combines RCM of butenoates **2** with a base-induced highly stereoselective ring opening of the transient metathesis products **4**, furnishing exclusively *Z*,*E*-dienes **3** [24]. We assume that the reaction proceeds via formation of an enolate **5**, followed by electrocyclic ring opening to carboxylates **6** [25], although a non-concerted pathway can not be excluded (Scheme 1).

**Figure 1:**
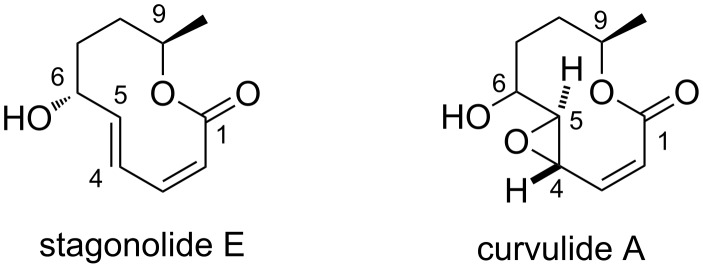
Structures and numbering scheme for stagonolide E and curvulide A.

To the best of our knowledge, metathesis/non-metathesis one flask sequences have not been used before for the two directional elaboration or desymmetrization of *C*_2_-symmetric building blocks. In this contribution, we demonstrate that this combination can be advantageously used for the synthesis of decanolides. These natural products share a ten-membered lactone structure and are normally isolated from fungi. Diverse bioactivities have been reported, such as phytotoxicity, cytotoxicity, antimalarial and antibacterial activity, which are a strong motivation for total synthesis [26–27]. In addition, several ambiguities in the structural assignment of some of these natural products still exist, and chemical synthesis has been proven to be a powerful and reliable tool for completing the structure elucidation and for correcting erroneous assignments. In particular if ex-chiral pool starting materials with well established absolute configurations are used, such as D-mannitol-derived **1** or L-tartrate-derived *ent*-**1**, highly reliable structural assignments become possible. Two decanolides, for which the absolute configuration was only assigned based on analogy to related natural products are stagonolide E [28–29] and curvulide A [30]. Stagonolide E is a secondary metabolite of *Stagonospora cirsii*, which is a fungal pathogen of the weed *Cirsium arvense* [28]. It has also been isolated from the fungus *Curvularia* sp. PSU-F22 [29]. Curvulide A was identified as a metabolite of a different strain of *Curvularia* sp. [30]. In this case, the absolute configuration at C9 was assigned as 9*R* based on a comparison of its CD spectrum with that of a structurally related compound, whereas the configuration at C6 could not be clarified. For the epoxide moiety of curvulide A, only the relative configurations at C4 and C5 were elucidated based on H,H-coupling constants (Figure 1) [30].

So far, two syntheses of stagonolide E have been published, which both rely on asymmetric synthesis for establishing both stereocenters. The crucial (2*Z*,4*E*)-configuration of the diene moiety was constructed via Still–Gennari olefination [31] or via RCM of an acrylate with an *E*-configured diene at the opposite terminus [32]. Curvulide A has, to the best of our knowledge, not been synthesized previously.

## Results and Discussion

**Scheme 2:**
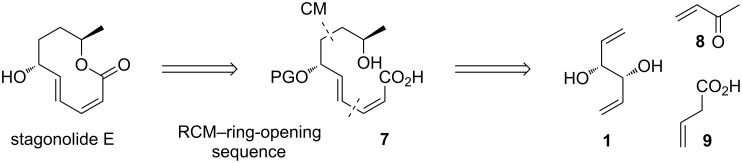
Synthetic plan for stagonolide E.

We planned to use a macrolactonization of precursor **7** as the cyclization step. For the synthesis of **7**, a cross metathesis of **1** (or a protected derivative) with methyl vinyl ketone (**8**) was envisaged as the first step, followed by an esterification of the more distant OH group with vinylacetic acid (**9**). This would provide a precursor **2** (Scheme 1) for the RCM-ring opening sequence (Scheme 2).

**Scheme 3:**
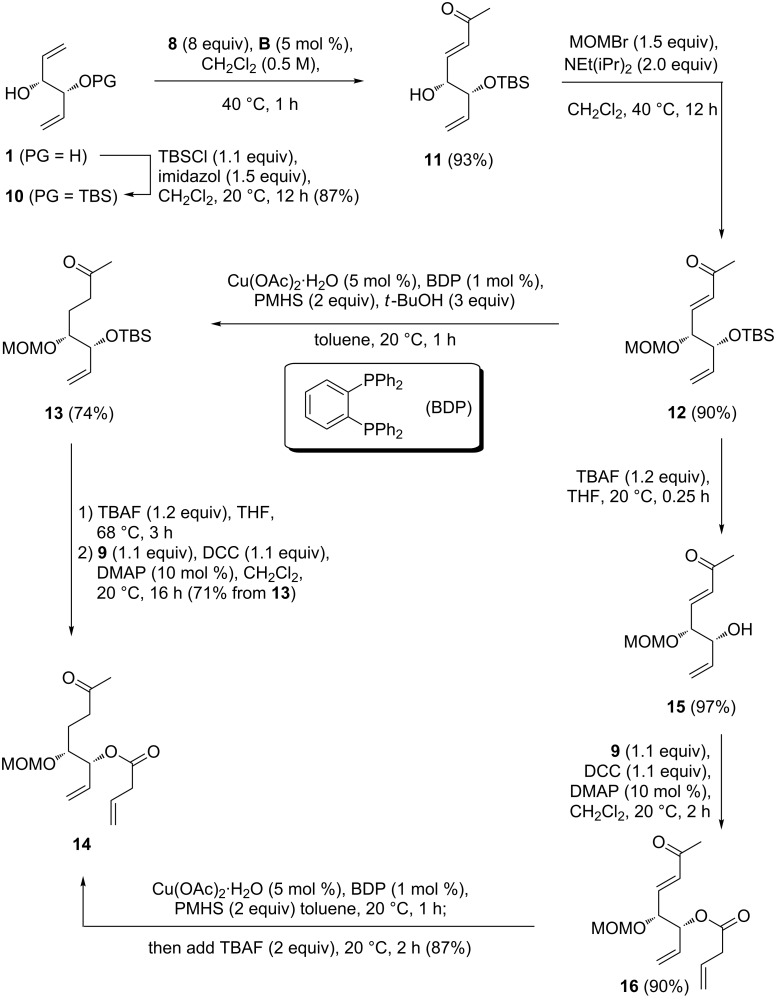
Synthesis of RCM/ring opening precursor **14**.

Our synthesis of stagonolide E started with a selective protection of one hydroxy group of diol **1** to furnish the known TBS-ether **10** [6,19], because we knew from previous experience that a desymmetrization based solely on cross metathesis would most likely be inefficient and yield substantial amounts of double functionalized product (Scheme 3) [9]. We tested methyl vinyl ketone (**8**) as a cross-metathesis partner, and the recently described Umicore M5_1_ initiator (**A**) [20,33–34], as well as Grubbs’ second generation carbene complex **B** [35] as precatalysts (Table 1). Phosphine-free precatalysts with a hemilabile alkoxy ligand are supposed to be well-suited for cross metathesis reactions [36–37]. With a moderate catalyst loading of 2.0 mol % of **A**, we isolated the expected cross metathesis product **11** in an acceptable yield of 76% (Table 1, entry 1). Surprisingly, the yield decreased considerably to 51% with an increased catalyst loading of 5.0 mol %, and formation of a byproduct with an *R*_f_ value very similar to that of the cross metathesis product **11** was observed by TLC (Table 1, entry 2). Inspection of the ^1^H NMR spectrum of the crude reaction mixture revealed that two singlets at 2.35 ppm and 6.75 ppm in a 1:3 ratio were present, which matches the NMR data previously reported for (*E*)-hex-3-ene-2,5-dione [38]. Obviously, catalyst **A** is sufficiently active to initiate the self metathesis of methyl vinyl ketone (**8**) to a considerable extent at a catalyst loading of 5.0 mol %, and consequently lower amounts of catalyst were tested in the following experiments. With 0.5 mol %, the isolated yield was 67% under otherwise identical conditions (Table 1, entry 3), and 85% of **11** were obtained with 1.0 mol % of **A** (Table 1, entry 4). With these catalyst loadings self metathesis of **8** is largely suppressed and the chromatographic isolation of **11** is facilitated, which might explain the improved yields under these conditions. With second generation Grubbs’ catalyst **B**, the self metathesis of the supposedly less reactive CM partner occurs only to a minor extent, even at elevated temperatures and catalyst loadings. When we used 2 mol % of **B** (Table 1, entry 5) the yield was 61%, but could be improved to 78% by adding phenol (Table 1, entry 6). The beneficial effect of phenol in cross metathesis reactions is known and has been attributed to a stabilization of the reactive 14-electron species [39]. A very high yield of 93% was eventually obtained by using 5 mol % of **B** in refluxing dichloromethane (Table 1, entry 7).

**Table 1:** Optimization of conditions for CM of **10** and methyl vinyl ketone (**8**).^a^

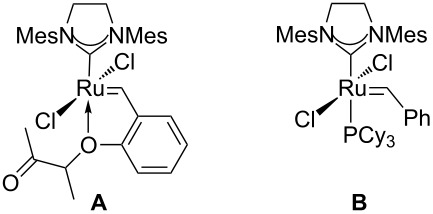				

entry	catalyst (mol %)	solvent	*T*	yield of **11**

1	**A** (2.0)	CH_2_Cl_2_	40 °C	76%
2^b^	**A** (5.0)	CH_2_Cl_2_	40 °C	51%
3	**A** (0.5)	CH_2_Cl_2_	40 °C	67%
4	**A** (1.0)	CH_2_Cl_2_	40 °C	85%
5	**B** (2.0)	toluene	80 °C	61%
6^c^	**B** (2.0)	toluene	80 °C	78%
7	**B** (5.0)	CH_2_Cl_2_	40 °C	93%

^a^General conditions: 8.0 equiv of **8**, initial substrate concentration: *c* = 0.5 M; ^b^formation of (*E*)-hex-3-ene-2,5-dione observed in the ^1^H NMR spectrum of the crude reaction mixture. ^c^With phenol (0.5 equiv) as additive.

In the next step, the remaining hydroxy group was protected as a MOM ether **12**, which was then selectively reduced at the electron-deficient double bond, using polymethylhydrosiloxane (PMHS) [40] as a reducing agent and a BDP–Cu hydride catalyst. This variant of Stryker’s reagent [41] has more recently been described by Lipshutz et al. [42] and was found to furnish ketone **13** in 74% yield. Desilylation of **13** could be accomplished quantitatively using TBAF at elevated temperature. However, an unidentified byproduct presumably a six-membered lacol was formed, which could not be separated by chromatography. Therefore the mixture was subjected to the second step, a Steglich esterification [43] with vinylacetic acid, to give the desired precursor **14** in pure form in 71% yield. To avoid the formation of the inseparable byproduct, we investigated a reversed order of steps. To this end, **12** was first desilylated to allyl alcohol **15**, which was then converted to butenoate **16**, again via Steglich esterification. For the selective reduction of the enoate **16**, the Stryker–Lipshutz protocol was again the method of choice and optimized conditions eventually furnished **14** in 87% yield (Scheme 3).

For the Stryker–Lipshutz reduction of **16** slightly different conditions were used than for the reduction of **12**. In particular, *tert*-butanol was omitted as a co-solvent, and TBAF was added to the reaction mixture after completed reduction. This modification was the result of an optimization study based on mechanistic considerations (Table 2) [44].

**Table 2:** Optimization of Cu–H-catalysed reduction of **16**.

entry	Cu(OAc)_2_·H_2_O (mol %)	BDP (mol %)	PMHS (equiv)	solvent	yield of **14**

1	5	1	2	toluene/*t*-BuOH (5:1)	72%
2	5	1	2	toluene/*t*-BuOH (2:1)	78%
3	1	0.5	1.2	toluene/*t*-BuOH (2:1)	67%
4^a^	5	1	2	toluene	87%

^a^TBAF (2 equiv) added after complete consumption of starting material.

The conditions previously used for the reduction of enoate **12** involved the use of *tert*-butanol as a co-solvent, together with toluene. Under these conditions, reproducible yields in the range between 67% and 78% were obtained (Table 2, entries 1–3). The alcohol is believed to protonate the Cu-enolate formed upon conjugate addition, resulting in the ketone and a Cu-alkoxide, which is then reduced with silane to regenerate the Cu-hydride. Alternatively, the Cu-enolate might enter a competing catalytic cycle by reacting with silane, furnishing a silyl enol ether and the catalytically active Cu-hydride species. The silyl enol ether is inert to protonation by *tert*-butanol, and therefore the competing secondary cycle will result in a decreased yield of reduction product. This reasoning prompted us to run the reaction in toluene without any protic co-solvent, which should exclusively lead to the silyl enol ether, and add TBAF as a desilylating agent after complete consumption of the starting material. The reduced product **14** was isolated under these conditions in 87% yield (Table 2, entry 4). With ketone **14** in hands, we decided to establish the required configuration at C9 in the next step. To this end, a CBS reduction [45–46] catalysed by the oxazaborolidine **17** was tested first (Table 3).

**Table 3:** Investigation of CBS reduction of ketone **14**.

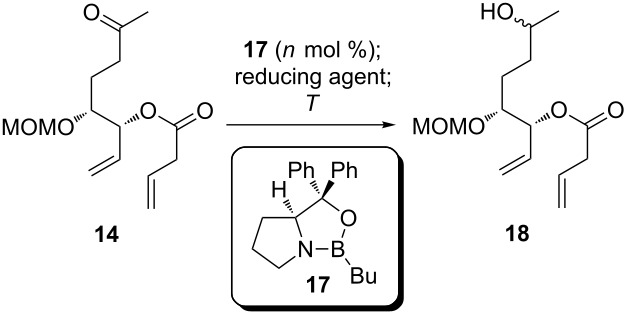				

entry	catalyst (mol %)	reducing agent	*T*	dr^a^

1	**17** (10)	H_3_B·SMe_2_	−78 °C	no conversion
2	**17** (20)	H_3_B·THF	20 °C	complex mixture
3	**17** (20)	H_3_B·THF	−50 °C	1:1
4	**17** (20)	catechol borane	−78 °C	3:2

^a^Determined from ^1^H NMR spectra of the crude reaction mixtures.

**Scheme 4:**
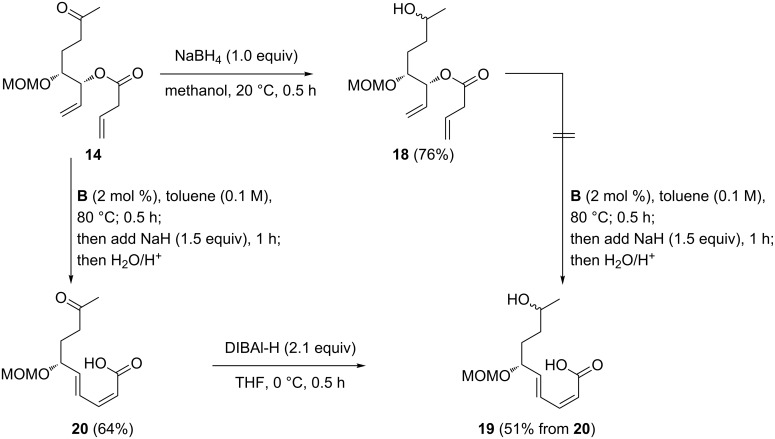
Synthesis of a substrate **19** for “late stage” resolution.

With borane–dimethylsulfide complex as the reductant and 10 mol % of catalyst, no conversion was observed at −78 °C (Table 3, entry 1), whereas attempted reduction at ambient temperature (Table 3, entry 2) resulted in the formation of a complex mixture, presumably due to competing hydroboration of the alkenes. With borane–THF at −50 °C the reduction proceeded to completion, but gave a 1:1 mixture of diastereomers (Table 3, entry 3). With catechol borane at −78 °C conversion was again complete, but the diastereoselectivity was far from being synthetically useful (Table 3, entry 4). Due to these rather discouraging results we did not pursue enantioselective reduction methods further to establish the required 9*R*-configuration, but considered a resolution approach. Ketone **14** was first reduced with NaBH_4_ to the expected diastereomeric mixture of alcohols **18**, which were then subjected to the conditions of the RCM/base-induced ring-opening sequence. Unfortunately, the expected macrolactonization precursor **19** was not obtained, but an inseparable mixture of products. To access the intended substrate for the resolution, secondary alcohol **19**, we investigated an inverted sequence of steps: ketone **14** was first converted to the 9-oxodienoic acid **20** under RCM/ring-opening conditions, followed by a reduction of the ketone with DIBAl-H to furnish **19**. Unfortunately, the yields obtained via this two-step sequence were only moderate and most likely to low to provide sufficient amounts of material for an efficient resolution (Scheme 4).

**Scheme 5:**
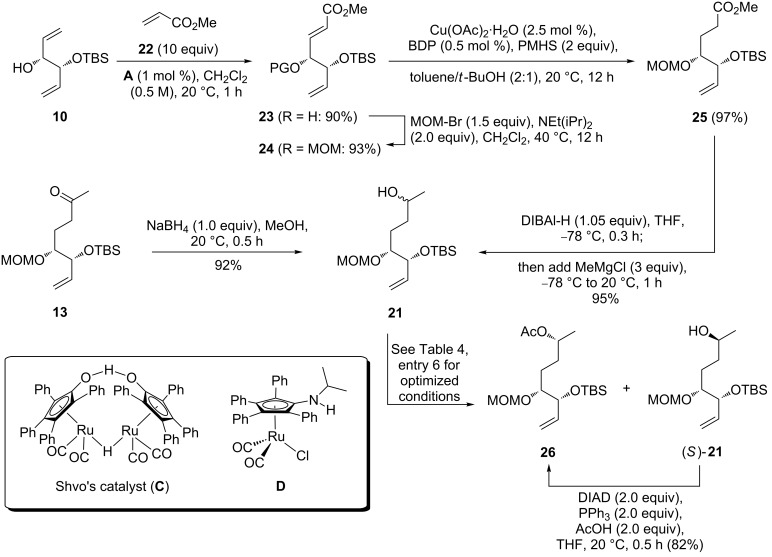
Synthesis of substrate **21** for “early stage” resolution.

These unsuccessful attempts to establish the correct configuration at C9 led to a revision of the synthetic strategy. We decided to investigate a dynamic kinetic resolution (DKR) approach at an earlier stage of the synthesis and identified the secondary alcohol **21** as a promising starting point for this approach (Scheme 5).

Compound **21** was obtained via two alternate routes, either by reduction of ketone **13** (Scheme 3) with NaBH_4_ or from ester **25** via one-flask reduction to the corresponding aldehyde and addition of methylmagnesium chloride. Ester **25** was in turn synthesized in three steps from monoprotected dienediol **10** via cross metathesis with methyl acrylate (**22**) [47] using a comparatively low loading of phosphine-free catalyst **A**, followed by MOM protection and Stryker–Lipshutz reduction of **24**. Notably the latter step proceeds significantly more efficient in a toluene/*tert*-butanol solvent mixture than the analogous enone reductions outlined in Scheme 3 and Table 2. Compared to these reactions, the saturated ester **25** was obtained in a nearly quantitative yield using half the amount of Cu precatalyst and BDP ligand.

In order to obtain enantiomerically pure **21**, an enzyme/transition metal-catalysed approach was investigated [48–49]. In this regard, the combination of Ru complexes such as Shvo’s catalyst (**C**) [50], the amino-Cp catalyst **D** [51], or [Ru(CO)_2_Cl(η^5^C_5_Ph_5_)] [52], and the lipase novozym 435 has emerged as particularly useful [53–54]. We tested Ru catalysts **C** and **D** under a variety of conditions (Table 4). In the absence of a Ru catalyst, a kinetic resolution occurs and **26** and the resolved alcohol (2*S*)-**21** were isolated in similar yields (Table 4, entry 1). Upon addition of Shvo’s catalyst **C**, only minor amounts of the desired acetate **26** and no resolved alcohol were obtained. Instead, the dehydrogenation product **13** was the predominant product (Table 4, entry 2). Addition of the base Na_2_CO_3_ led only to a small improvement (Table 4, entry 3). Ketone formation has previously been described in attempted DKR’s of secondary alcohols when catalyst **C** was used in combination with isopropenyl or vinyl acetate as acylating agents [54]. For this reason, the aminocyclopentadienyl–Ru complex **D** was evaluated next. Very similar results were obtained with a catalytic amount of KO*t*-Bu in the presence or absence of a stoichiometric amount of Na_2_CO_3_ as a base, at ambient temperature and a reaction time of one week (Table 4, entries 4 and 5). A slightly elevated temperature led to a significantly improved yield of 67% of **26** and 31% of (2*S*)-**21**. Both compounds were obtained in a diastereomeric ratio higher than 19:1, as judged from the ^1^H NMR spectrum (Table 4, entry 6). In an attempt to further improve the yield of **26**, the amount of catalyst **D** was increased to 5 mol %. This resulted indeed in an increased amount of **26**, but at the expense of diastereoselectivity, which dropped to 6:1 (Table 4, entry 7). A prolonged reaction time of 14 d led, under otherwise identical conditions, to a slightly improved yield, but at the same time to an even more dramatic erosion of diastereoselectivity (dr = 3:1, Table 4, entry 8). Thus, the conditions listed in Table 4, entry 6 were identified as the optimum. As the alcohol (2*S*)-**21** could also be isolated in a diastereomeric ratio higher than 19:1, it was converted to **26** via Mitsunobu inversion [55] with acetic acid as the nucleophile.

**Table 4:** Optimization of conditions for Ru–lipase-catalysed DKR of **21**.

entry	conditions^a^	**26**	(2*S*)-**21**^b,c^	**13**^c^

1^d^	Novozym 435, iPPA (1.0 equiv), toluene, 20 °C, 24 h	49%	44%	n. d.
2^d^	**C** (2 mol %), Novozym 435, iPPA (10.0 equiv), toluene, 70 °C, 72 h	17%	n. d.	65%
3^d^	**C** (1 mol %), Novozym 435, iPPA (10.0 equiv), Na_2_CO_3_ (1.0 equiv), toluene, 70 °C, 24 h	30%	n. d.	30%
4^d^	**D** (2 mol %), Novozym 435, iPPA (1.5 equiv), Na_2_CO_3_ (1.0 equiv); *t*-BuOK (5 mol %), toluene, 20 °C, 7 d	50%	38%	n. d.
5^d^	**D** (2 mol %); Novozym 435, iPPA (1.5 equiv), *t*-BuOK (5 mol %), toluene, 20 °C, 7 d	50%	n. i.	n. d.
6^d^	**D** (2 mol %), Novozym 435, iPPA (3.0 equiv), Na_2_CO_3_ (1.0 equiv), *t*-BuOK (3 mol %), toluene, 30 °C, 7 d	67%	31%	n. d.
7^e^	**D** (5 mol %), Novozym 435, iPPA (1.5 equiv), Na_2_CO_3_ (1.0 equiv), *t*-BuOK (6 mol %), toluene, 30 °C, 5 d	76%	20%	n. d.
8^f^	**D** (5 mol %), Novozym 435, iPPA (3.0 equiv), Na_2_CO_3_ (1.0 equiv), *t*-BuOK (6 mol %), toluene, 30 °C, 14 d	80%	n. i.	n. d.

^a^iPPA: isopropenyl acetate; ^b^n. d.: not determined; ^c^n. i.: not isolated; ^d^dr’s of **26** and (2*S*)-**21** >19:1; ^e^dr of **26** = 6:1; ^f^dr of **26** = 3:1.

**Scheme 6:**
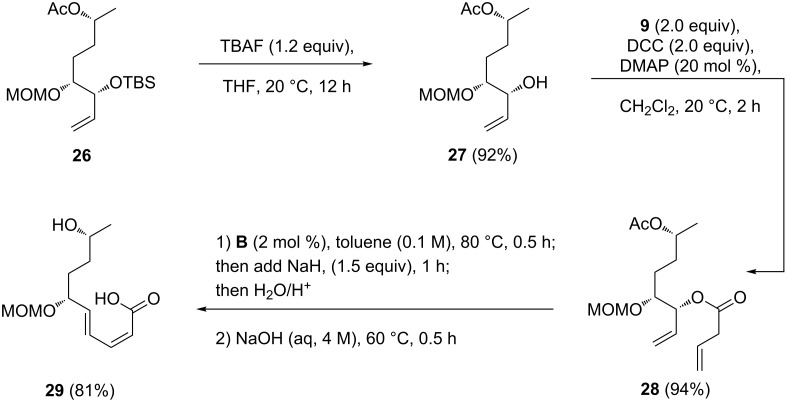
Synthesis of macrolactonization precursor **29**.

The synthesis of stagonolide E commenced with the desilylation of **26** and Steglich esterification of the resulting allyl alcohol **27**. One-flask reaction of **28** with catalyst **B**, followed by treatment with NaH, resulted in the expected RCM/ring-opening sequence, but also in a partial deacetylation. For this reason, the crude reaction mixture was subsequently treated with aqueous NaOH to complete the ester cleavage, giving the macrolactonization precursor **29** [31] in 81% yield (Scheme 6).

**Scheme 7:**
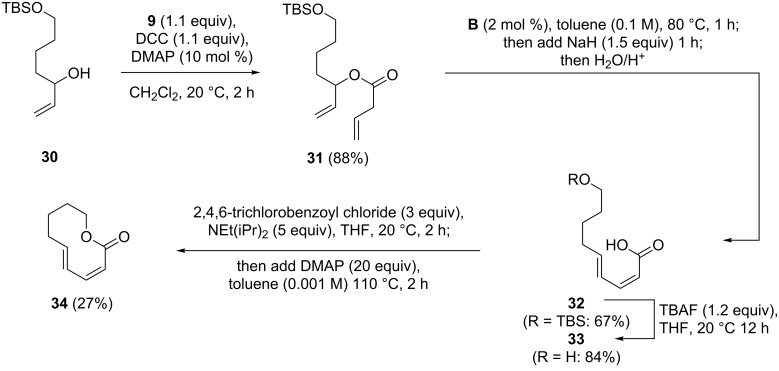
Synthesis of (2*Z*,4*E*)-9-hydroxy-2,4-dienoic acid (**33**) and its macrolactonization.

In a previous study [24], we had investigated the macrolactonization of the 6-deoxygenated derivative of **29**, which is itself a natural product named curvulalic acid (**35**) [29], and experienced enormous difficulties. No conversion to the expected cyclization product, another naturally occurring decanolide named fusanolide A (**36**) [56], was observed using the Steglich [43], Mukaiyama [57], Yamaguchi [58] or Shiina method [59] under their published standard conditions. For these reasons, we decided to investigate whether the macrolactonization of (2*Z*,4*E*)-9-hydroxy-2,4-dienoic acids is generally hampered, which might be caused by the build-up of ring strain. We started this investigation with the simple derivative **33**, which was synthesized from **30** [60] via a sequence of three steps. For the macrolactonization of **33** we chose Yamaguchi’s method, but applied significantly more forcing conditions by using increased amounts of reagents and in particular a large excess of DMAP, in combination with higher dilution and elevated reaction temperatures. This led indeed to the formation of the desired lactone **34**, which could be isolated in a moderate yield of 27% (Scheme 7).

**Scheme 8:**
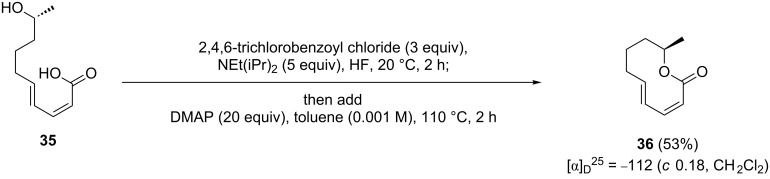
Synthesis of published structure of fusanolide A (**36**).

With this result in hand, we reinvestigated the cyclization of **35** [24] to fusanolide A (**36**) under the conditions outlined above. Gratifyingly, **36** was obtained in a yield of 53%, which allowed us to compare its analytical data with those reported for natural fusanolide A [56]. This comparison confirmed our previously suggested revision of the ten-membered lactone structure originally assigned to fusanolide A, as the spectroscopic data obtained for synthetic **36** differ significantly from those reported for the natural product. As we mentioned in our previous publication describing the synthesis of curvulalic acid (**35**) [24], all spectroscopic data obtained for this compound match those reported for fusanolide A [56] perfectly, suggesting that curvulalic acid and fusanolide A are probably identical. It should, however, be noted that **36** might well be a natural product which has not yet been isolated from a natural source (Scheme 8).

**Scheme 9:**
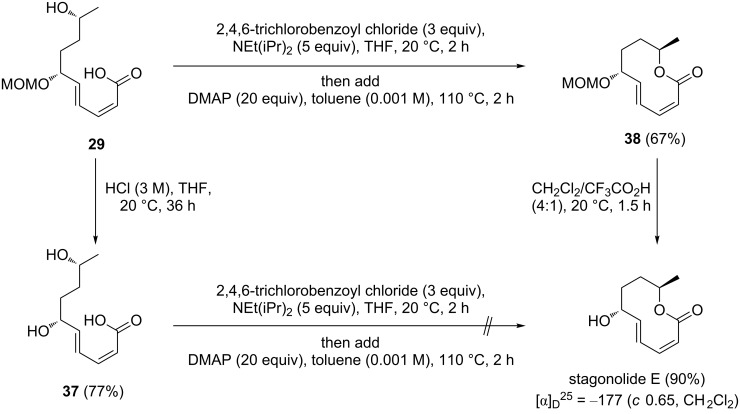
Completion of stagonolide E synthesis.

To complete the synthesis of stagonolide E, the MOM-protected precursor **29** and the deprotected derivative **37** were subjected to the Yamaguchi conditions that were found to be successful for the synthesis of **34** and **36** (Scheme 9). While the attempted Yamaguchi lactonization of **37** failed completely and resulted only in the quantitative recovery of unreacted starting material, the 6-MOM-protected precursor **29** underwent cyclization to the protected decanolide **38** [31] in 67% yield. Deprotection of **38** was accomplished with TFA in dichloromethane at ambient temperature without noticeable epimerization or elimination of water. Stagonolide E was isolated in 90% yield and its analytical data were identical to those reported for the natural product [28].

Only few examples for the macrolactonization of ω-hydroxy-2*Z*,4*E*-dienoic acids such as **29**, **33** and **34** have been described in the literature, and we are not aware of another study which describes the cyclization of differently substituted derivatives under identical conditions. Notably, the yield of macrolactones is significantly affected by the substitution pattern and increases from 27% for the unsubstituted lactone **34** (Scheme 7) to 53% for the 9-methyl-substituted derivative **36** (Scheme 8) and to 67% for the 6,9-disubstituted compound **38** (Scheme 9). The presence of substituents and their relative configuration may have severe conformational effects on transition states, activation barriers and product stability [61–62]. An example for which a dramatically increased yield was reported upon incorporation of substituents has been reported in the course of an octalactin synthesis [61].

**Scheme 10:**
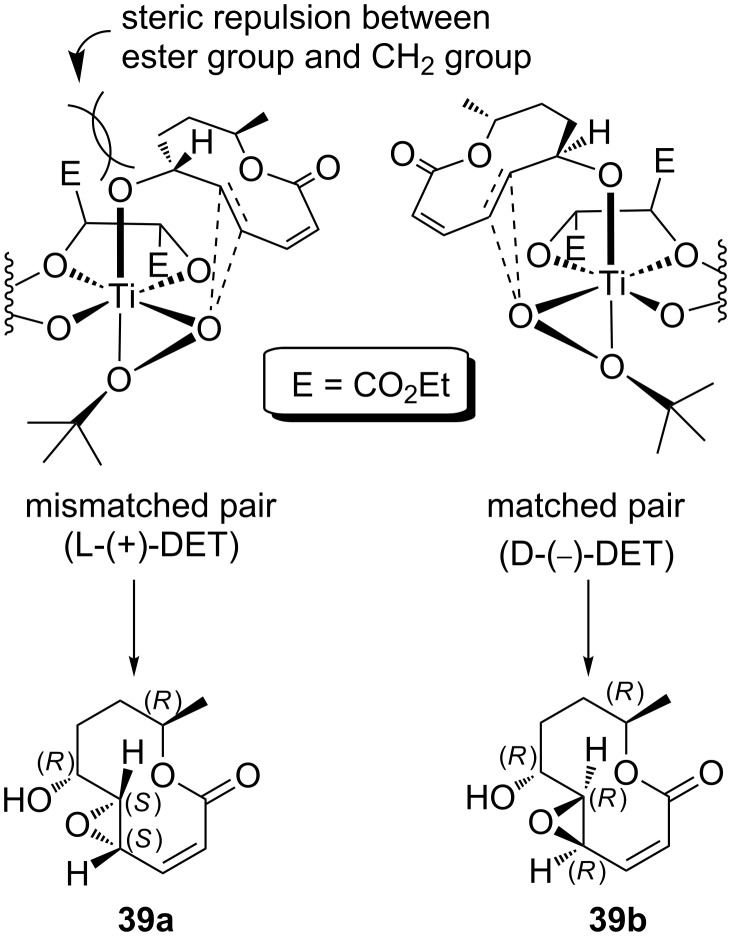
Transition-state models for the Sharpless epoxidation of stagonolide E with L-(+)-DET (left) and D-(−)-DET (right).

Having established a reliable route to stagonolide E, we investigated its epoxidation under Sharpless conditions [63]. We expected that this transformation would give either curvulide A [30] or one of its diastereomers, and help to resolve the remaining structural ambiguities, i.e. the absolute configurations at C4, C5 and C6. Based on the transition-state model for the Sharpless epoxidation of allylic alcohols bearing a stereogenic centre in the allylic position [64], we expected that levorotatory stagonolide E and L-(+)-diethyl tartrate (DET) should form the mismatched pair, while the matched pair would result with D-(−)-DET (Scheme 10).

**Scheme 11:**
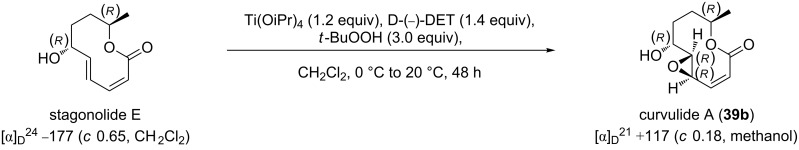
Synthesis of **39b** (curvulide A) from stagonolide E.

We subjected (−)-stagonolide E to the conditions of a Sharpless epoxidation, using both L-(+)-DET and D-(−)-DET. As expected on the basis of the transition-state model, no reaction occurred after 2 d with L-(+)-DET, and the starting material could be recovered nearly quantitatively. In contrast, the use of D-(−)-DET led to the formation of an epoxide **39b** in 58% yield. A comparison of the analytical data of **39b** with those reported for curvulide A revealed that the NMR spectroscopic data are identical, and the value for the specific rotation of **39b** is reasonably close to the value reported for the natural product ([α]_D_^23^ +133) [30]. Therefore, we conclude that the Sharpless-epoxidation product of stagonolide E is identical with curvulide A and suggest the (4*R*,5*R*,6*R*,9*R*)-configuration shown for **39b** (Scheme 11).

**Figure 2:**
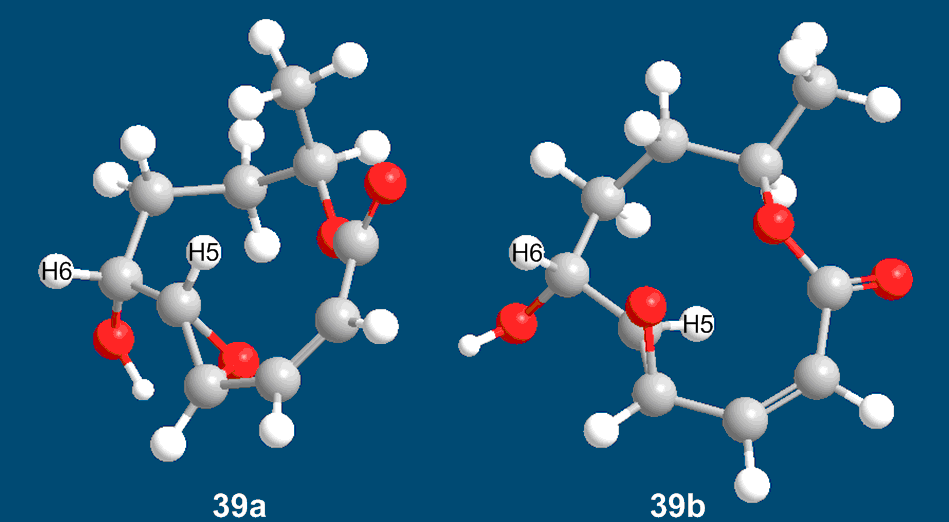
MM2 energy-minimized structures of **39a** and **39b**.

While the *R*-configuration assigned to C6 and C9 is unequivocally established, because these stereocenters originate from stagonolide E, there still remains an uncertainty for the absolute configurations at C4 and C5. While the relative *trans*-configuration at these stereocenters is evident from a small ^3^*J*(H4–H5) value of 2.2 Hz and from the *E*-configuration of the precursor, the relative configuration of C6 and C5, and hence the absolute configurations at C4 and C5, can not be assigned with absolute reliability. However, a comparatively large coupling constant ^3^*J*(H5–H6) of 8.2 Hz is pointing towards a *trans*-orientation of these protons with a large dihedral angle. Unfortunately, we could not obtain the (4*S*,5*S*,6*R*,9*R*)-configured **39a** and compare the crucial ^3^*J*(H5–H6) coupling constants of the two diastereoisomers. However, the calculated energy-minimized structures of **39a** and **39b** suggest that the H5–H6 dihedral angles should differ substantially (Figure 2). For **39a**, this angle should be close to 90°, which is not in agreement with a coupling constant of 8.2 Hz. In contrast, the same dihedral angle can be expected to be approximately 170° in the case of the diastereomeric epoxide **39b**, and this value fits well to the observed ^3^*J*(H5–H6) value (Figure 2) [65].

## Conclusion

In summary, we synthesized the naturally occurring ten-membered lactones stagonolide E and curvulide A, starting from the ex-chiral pool building block (*R*,*R*)-hexa-1,5-diene-3,4-diol. Key elements of the stagonolide E synthesis are the two-directional functionalization of the enantiopure, *C*_2_-symmetrical starting material through cross metathesis and a one-flask ring-closing metathesis/base-induced ring-opening sequence, a Ru–lipase-catalyzed dynamic kinetic resolution to establish the stereochemistry at C6, and a Yamaguchi macrolactonization. The first synthesis of curvulide A was accomplished by Sharpless epoxidation of stagonolide E. While the previously assigned absolute and relative configurations of stagonolide E could be confirmed by the synthesis described herein, we were able to provide additional information concerning missing structural assignments for curvulide A, which is most likely (4*R*,5*R*,6*R*,9*R*)-configured.

## Supporting Information

File 1 Experimental procedures, characterization data and copies of ^1^H and ^13^C NMR spectra.
