# Supplementary material for: Bidirectional cross metathesis and ring-closing metathesis/ring opening of a C2-symmetric building block: a strategy for the synthesis of decanolide natural products
Source: Beilstein J Org Chem. 2013 Nov 18;9:2544–55. doi: 10.3762/bjoc.9.289 (PMC3869341; doi:10.3762/bjoc.9.289)

Supporting Information

for

Bidirectional cross metathesis and ring-closing metathesis/ring opening of a C_2-symmetric building block: a strategy for the synthesis of decanolide natural products

Bernd Schmidt* and Oliver Kunz

Address: Institut für Chemie, Organische Synthesechemie, Universität Potsdam, Karl-Liebknecht-Straße 24-25, 14476 Potsdam-Golm, Germany

Email: Bernd Schmidt - bernd.schmidt@uni-potsdam.de

*Corresponding author

Experimental procedures, characterization data and copies of ^{1}H and ^{13}C NMR spectra

Table of contents

A General remarks S4

B Experimental procedures, analytical data and copies of spectra S5

B1 (5*R*,6*R*,*E*)-6-(*tert*-butyldimethylsilyloxy)-5-hydroxyocta-3,7-dien-2-one (**11**) S5

B2 (5*R*,6*R*,*E*)-6-(*tert*-butyldimethylsilyloxy)-5-(methoxymethoxy)octa-3,7-dien-2-one (**12**) S8

B3 (5*R*,6*R*)-6-(*tert*-butyldimethylsilyloxy)-5-(methoxymethoxy)oct-7-en-2-one (**13**) S11

B4 (3*R*,4*R*)-4-(methoxymethoxy)-7-oxooct-1-en-3-yl but-3-enoate (**14**) S14

B5 (5*R*,6*R*,*E*)-6-hydroxy-5-(methoxymethoxy)octa-3,7-dien-2-one (**15**) S18

B6 (3*R*,4*R*,*E*)-4-(methoxymethoxy)-7-oxoocta-1,5-dien-3-yl but-3-enoate (**16**) S21

B7 (3*R*,4*R*)-7-hydroxy-4-(methoxymethoxy)oct-1-en-3-yl but-3-enoate (**18**) S24

B8 (*R*,2*Z*,4*E*)-9-hydroxy-6-(methoxymethoxy)deca-2,4-dienoic acid (**19**) S27

B9 (*R*,2*Z*,4*E*)-6-(methoxymethoxy)-9-oxodeca-2,4-dienoic acid (**20**) S30

B10 (4*R*,5*R*)-methyl 5-(*tert*-butyldimethylsilyloxy)-4-(methoxymethoxy)hept-6-enoate (**21**) S33

B11 (4*R*,5*R*,*E*)-methyl 5-(*tert*-butyldimethylsilyloxy)-4-hydroxyhepta-2,6-dienoate (**23**) S37

B12 (4*R*,5*R*,*E*)-methyl-5-(*tert*-butyldimethylsilyloxy)-4-(methoxymethoxy)hepta-2,6-dienoate (**24**) S40

B13 (4*R*,5*R*)-methyl 5-(*tert*-butyldimethylsilyloxy)-4-(methoxymethoxy)hept-6-enoate (**25**) S43

B14 (2*R*,5*R*,6*R*)-6-(*tert*-butyldimethylsilyloxy)-5-(methoxymethoxy)oct-7-en-2-yl acetate (**26**) and (2*S*,5*R*,6*R*)-6-(*tert*-butyldimethylsilyloxy)-5-(methoxymethoxy)oct-7-en-2-ol ((S)-**21**) S46

B15 (2*R*,5*R*,6*R*)-6-hydroxy-5-(methoxymethoxy)oct-7-en-2-yl acetate (**27**) S52

B16 (3*R*,4*R*,7*R*)-7-acetoxy-4-(methoxymethoxy)oct-1-en-3-yl but-3-enoate (**28**) S55

B17 (2*Z*,4*E*,6*R*,9*R*)-9-hydroxy-6-(methoxymethoxy)deca-2,4-dienoic acid (**29**) S58

B18 7-(*tert*-butyldimethylsilyloxy)hept-1-en-3-yl but-3-enoate (**31**) S61

B19 (2*Z*,4*E*)-9-(*tert*-butyldimethylsilyloxy)nona-2,4-dienoic acid (**32**) S64

B20 (2*Z*,4*E*)-9-hydroxynona-2,4-dienoic acid (**33**) S67

B21 (3*Z*,5*E*)-7,8,9,10-tetrahydro-2*H*-oxecin-2-one (**34**) S70

B22 (*R*,3*Z*,5*E*)-10-methyl-7,8,9,10-tetrahydro-2*H*-oxecin-2-one (**36**) S73

B23 (2*Z*,4*E*,6*R*,9*R*)-6,9-dihydroxydeca-2,4-dienoic acid (**37**) S76

B24 (3*Z*,5*E*,7*R*,10*R*)-7-(methoxymethoxy)-10-methyl-7,8,9,10-tetrahydro-2*H*-oxecin-2-one (**38**) S79

B25 Stagonolide E S82

B26 Curvulide A S85

A General Remarks

^{1}H NMR spectra were obtained at 300 MHz in $CDCl_3$, acetone-d_6 and MeOD-d_4 with $CHCl_3$ (δ = 7.26 ppm), acetone-d_5 (δ = 2.05 ppm) and MeOD-d_3 (δ = 3.31 ppm) as an internal standard. Coupling constants (*J*) are given in Hz. ^{13}C NMR spectra were recorded at 75 MHz in $CDCl_3$, acetone-d_6 and MeOD-d_4 with $CHCl_3$ (δ = 77.0 ppm), acetone-d_5 (δ = 29.9 ppm) and MeOD-d_3 (δ = 49.2 ppm) as an internal standard. The number of coupled protons was analysed by DEPT or APT experiments and is denoted by a number in parentheses following the chemical shift value. IR spectra were recorded as neat films on NaCl or KBr plates or as KBr discs. Wavenumbers (ν) are given in cm^{-1}. The peak intensities are defined as strong (s), medium (m), or weak (w). Low- and high resolution mass spectra were obtained by ESI/TOF. These compounds were synthesized following literature procedures: **10**[1], **30**[2] and **35**[3].

[1] Quinn, K. J.; Isaacs, A. K.; DeChristopher, B. A.; Szklarz, S. C.; Arvary, R. A. *Org. Lett.* **2005**, *7*, 1243-1245.
[2] Enders, D.; Nguyen, D. *Synthesis* **2000**, 2092-2098.
[3] Schmidt, B.; Kunz, O. *Eur. J. Org. Chem.* **2012**, 1008-1018.

B Experimental procedures and analytical data

*B1 (5R,6R,E)-6-(tert-Butyldimethylsilyloxy)-5-hydroxyocta-3,7-dien-2-one (**11**)*

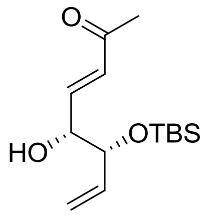

To a solution of **10** (200 mg, 0.88 mmol) in CH_2Cl_2 (1.8 mL) were added but-3-en-2-one (0.58 mL, 7.0 mmol) and Ru catalyst **B** (37 mg, 5 mol %) at 40 °C. The solution was stirred for 1 h at 40 °C, and the solvent was evaporated. The residue was purified by column chromatography on silica (eluent hexanes/MTBE 10:1) to give **11** (220 mg, 93%) as a colourless oil: $[\alpha]^{24}_D$ = +18.9 (*c* 0.49, CH_2Cl_2); ^1H NMR (300 MHz, $CDCl_3$) δ 6.73 (dd, *J* = 16.0, 4.4, 1H), 6.37 (dd, *J* = 16.0, 1.6, 1H), 5.82 (ddd, *J* = 17.2, 10.4, 6.7, 1H), 5.27 (d, *J* = 17.2, 1H), 5.25 (d, *J* = 10.3, 1H), 4.15 (m, 1H), 4.04 (dd, *J* = 6.6, 5.7, 1H), 2.66 (d, *J* = 3.8, 1H), 2.25 (s, 3H), 0.90 (s, 9H), 0.06 (s, 3H), 0.05 (s, 3H); ^{13}C NMR (75 MHz, $CDCl_3$) δ 197.9 (0), 144.9 (1), 137.2 (1), 130.7 (1), 117.9 (2), 77.0 (1), 74.1 (1), 27.5 (3), 25.7 (3), 18.1 (0), −4.2 (3), −4.9 (3); IR (neat) ν 3445 (w), 2929 (w), 2857 (w), 1676 (m), 1631 (w), 1423 (s), 1252 (s); HRMS (ESI) calcd. for $C_{14}H_{27}O_3Si^+$ ($[M+H]^+$) 271.1729, found 271.1729; Anal. calcd for $C_{14}H_{26}O_3Si$: C, 62.2; H, 9.7; Found: C, 62.0; H, 9.9.

Alternatively, **11** (202 mg, 85%) was obtained from **10** (200 mg, 0.88 mmol) using Ru catalyst **A** (5.8 mg, 1 mol %) under otherwise identical conditions.

^{1}H NMR (300 MHz, $CDCl_3$) of **11**

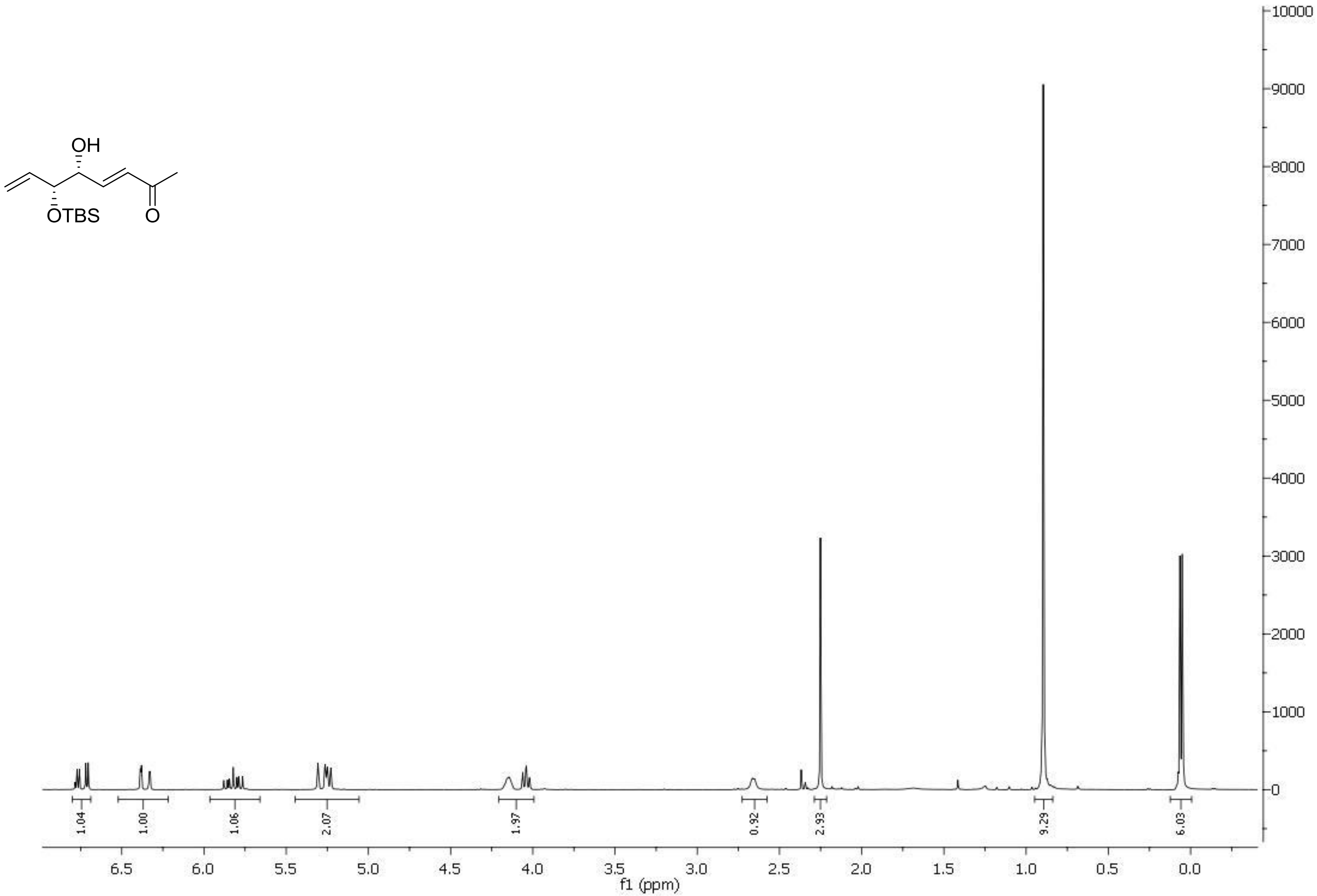

^{13}C NMR-APT (75 MHz, $CDCl_3$) of **11**

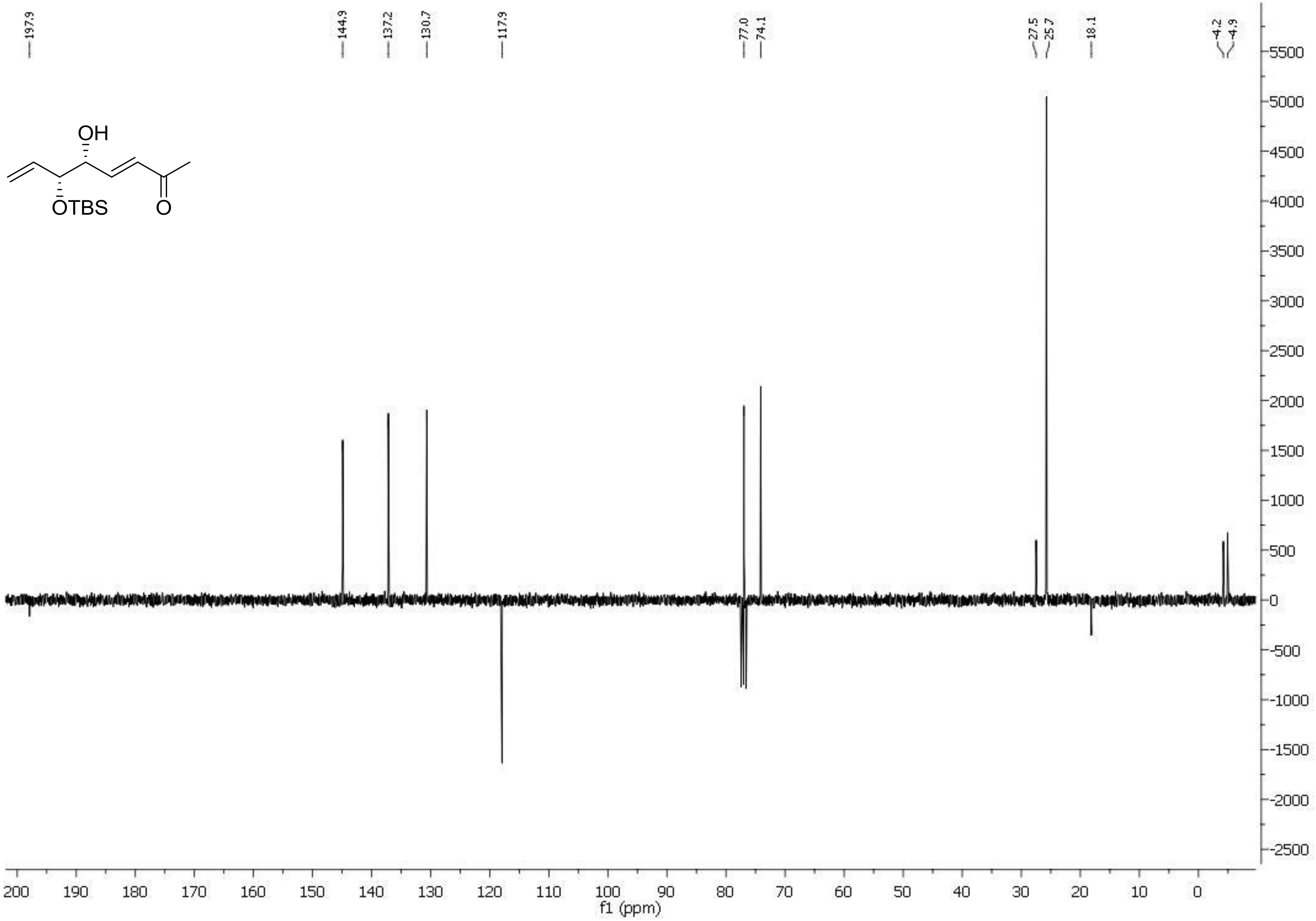

*B2 (5R,6R,E)-6-(tert-Butyldimethylsilyloxy)-5-(methoxymethoxy)octa-3,7-dien-2-one (**12**)*

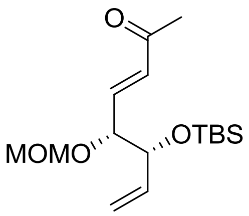

To a solution of **11** (2.0 g, 7.4 mmol) in CH_2Cl_2 (74 mL) were added DIPEA (2.6 mL, 14.8 mmol) and MOM-Br (0.91 mL, 11.1 mol) at 0 °C. The solution was stirred for 12 h at 40 °C. After this time, water (50 mL) was added and the aqueous layer was extracted three times with MTBE. The organic layers were dried with $MgSO_4$, filtered, and evaporated. The residue was purified by column chromatography on silica (eluent: hexanes/MTBE 10:1) to give **12** (2.1 g, 90%) as a colorless oil: $[\alpha]^{24}_D$ = +27.4 (*c* 0.54, CH_2Cl_2); ^{1}H NMR (300 MHz, $CDCl_3$) δ 6.70 (dd, *J* = 16.2, 5.3, 1H), 6.24 (dd, *J* = 16.2, 1.3, 1H), 5.82 (ddd, *J* = 17.1, 10.5, 5.6, 1H), 5.25 (dd, *J* = 17.1, 1.5, 1H), 5.17 (dd, *J* = 10.5, 1.2, 1H), 4.68 (d, *J* = 6.8, 1H), 4.64 (d, *J* = 6.8, 1H), 4.27 (dd, *J* = 5.5, 5.5, 1H), 4.19 (ddd, *J* = 5.4, 5.4, 1.3, 1H), 3.36 (s, 3H), 2.24 (s, 3H), 0.89 (s, 9H), 0.07 (s, 3H), 0.05 (s, 3H); ^{13}C NMR (75 MHz, $CDCl_3$) δ 143.6 (1), 136.7 (1), 131.9 (1), 116.6 (2), 95.7 (2), 79.0 (1), 75.0 (1), 55.7 (3), 27.0 (3), 25.8 (3), 18.2 (0), −4.7 (3), −5.0 (3); IR (neat) *ν* 2954 (w), 2950 (w), 2857 (w), 1681 (s), 1633 (w), 1360 (m), 1252 (s); MS (ESI) *m/z* 183 (12), 253 (15), 337 ($[M+Na]^+$, 100); HRMS (ESI) calcd for $C_{16}H_{30}O_4SiNa^+$ ($[M+Na]^+$) 337.1811, found 337.1790; Anal. Calcd for $C_{16}H_{30}O_4Si$: C, 61.1; H, 9.6; Found: C, 61.4; H, 9.6.

^{1}H NMR (300 MHz, $CDCl_3$) of **12**

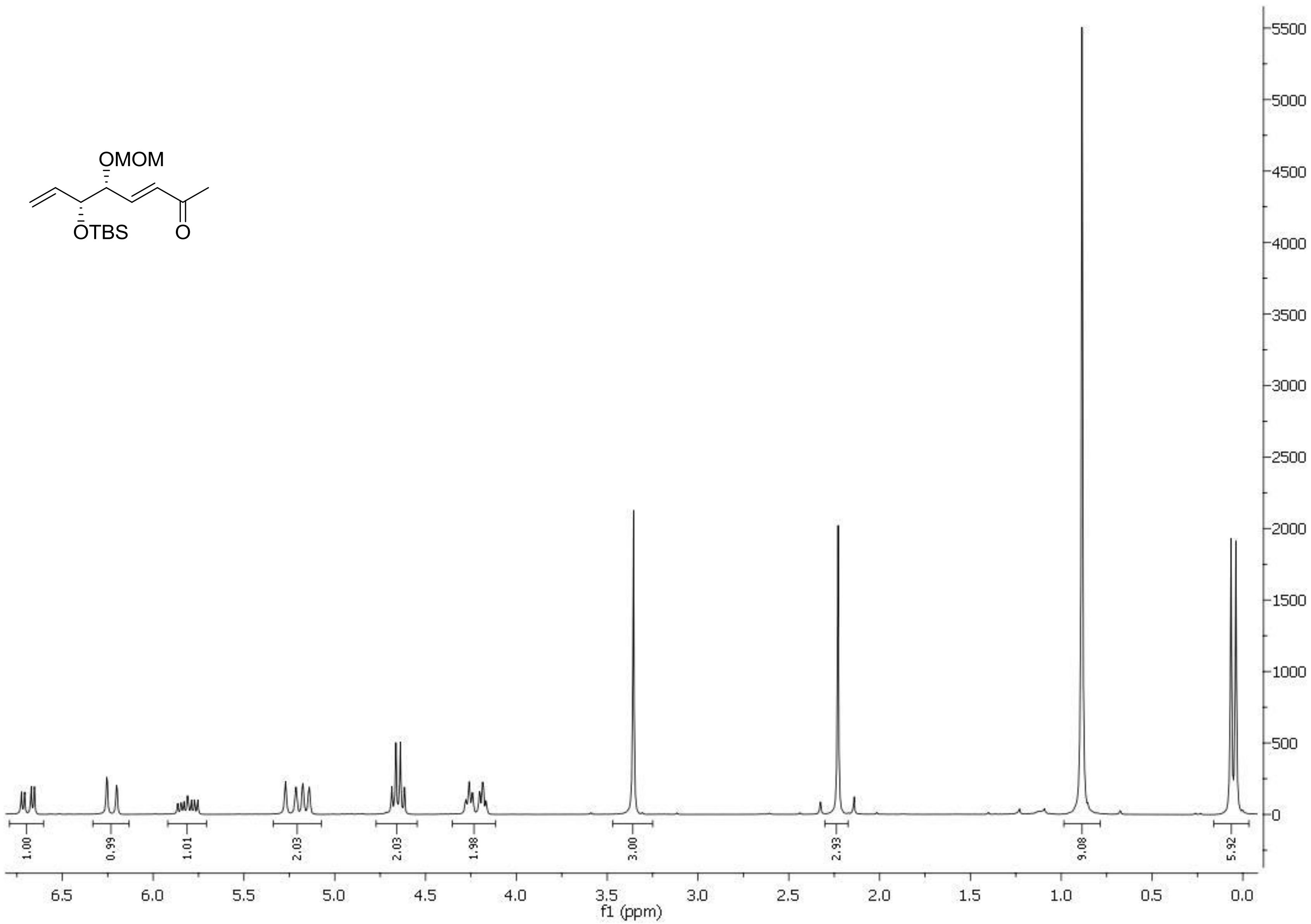

^{13}C NMR-APT (75 MHz, $CDCl_3$) of **12**

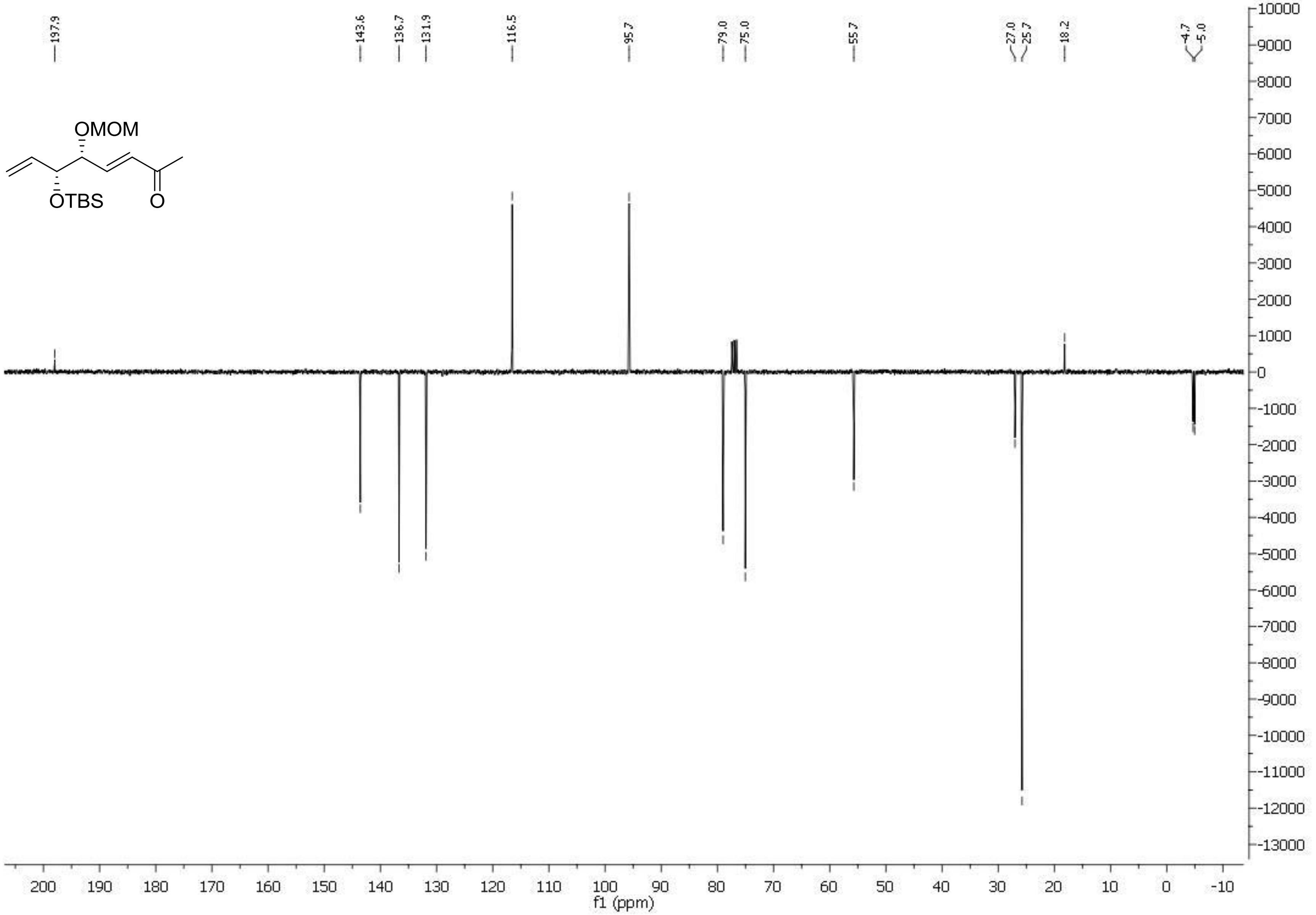

*B3 (5R,6R)-6-(tert-Butyldimethylsilyloxy)-5-(methoxymethoxy)oct-7-en-2-one (**13**)*

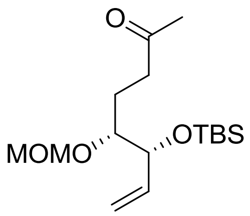

$Cu(OAc)_2 \cdot H_2O$ (10 mg, 5 mol %) and BDP (4.5 mg, 1 mol %) were dissolved in dry, degassed toluene (2.0 mL) and *tert*-butanol (1.5 mL). The mixture was stirred for 10 min at ambient temperature, and PMHS (134 µL, 2.0 mmol) was added. Stirring was continued for 0.5 h, after which time the colour changed from blue to green. A solution of **12** (314 mg, 1.0 mmol) in toluene (1.0 mL) was added and the solution was stirred at ambient temperature for 1 h. The reaction mixture was diluted with MTBE, washed with a saturated aqueous $NaHCO_3$ solution, followed by aqueous HCl (1 M). The aqueous layer was extracted twice with MTBE. The organic layers were dried with $MgSO_4$, filtered, and evaporated. The residue was purified by column chromatography on silica (eluent: hexanes/MTBE 10:1) to give **13** (233 mg, 74%) as a colourless oil: $[\alpha]^{24}_D = +28.2$ (*c* 0.55, CH_2Cl_2); ^1H NMR (300 MHz, $CDCl_3$) δ 5.86 (ddd, *J* = 17.2, 10.5, 5.4, 1H), 5.25 (dd, *J* = 17.2, 1.5, 1H), 5.15 (d, *J* = 10.5, 1H), 4.69 (d, *J* = 6.8, 1H), 4.60 (d, *J* = 6.8, 1H), 4.20 (dd, *J* = 5.4, 5.4, 1H), 3.41 (ddd, *J* = 9.0, 5.2, 3.7, 1H), 3.36 (s, 3H), 2.25 (td, *J* = 7.7, 2.5, 2H), 2.10 (s, 3H), 1.87 (m, 1H), 1.55 (m, 1H), 0.88 (s, 9H), 0.05 (s, 3H), 0.03 (s, 3H); ^{13}C NMR (75 MHz, $CDCl_3$) δ 208.5 (0), 137.0 (1), 116.0 (2), 97.4 (2), 81.0 (1), 75.1 (1), 55.7 (3), 39.9 (2), 29.6(3), 25.8 (3), 24.2 (2), 18.1 (0), −4.7 (3), −5.0 (3); IR (neat) *ν* 2930 (w), 2857 (w), 1717 (s), 1361 (m), 1252 (s), 1030 (s); MS (ESI) *m/z* 183 (12), 253 (15), 337 ([M+Na]$^+$, 100); HRMS (ESI) calcd for $C_{16}H_{30}O_4SiNa^+$ ([M+Na]$^+$) 337.1811, found 337.1790; Anal. Calcd for $C_{16}H_{32}O_4Si$: C, 60.7; H, 10.2; Found: C, 60.5; H, 10.5.

^{1}H NMR (300 MHz, $CDCl_3$) of **13**

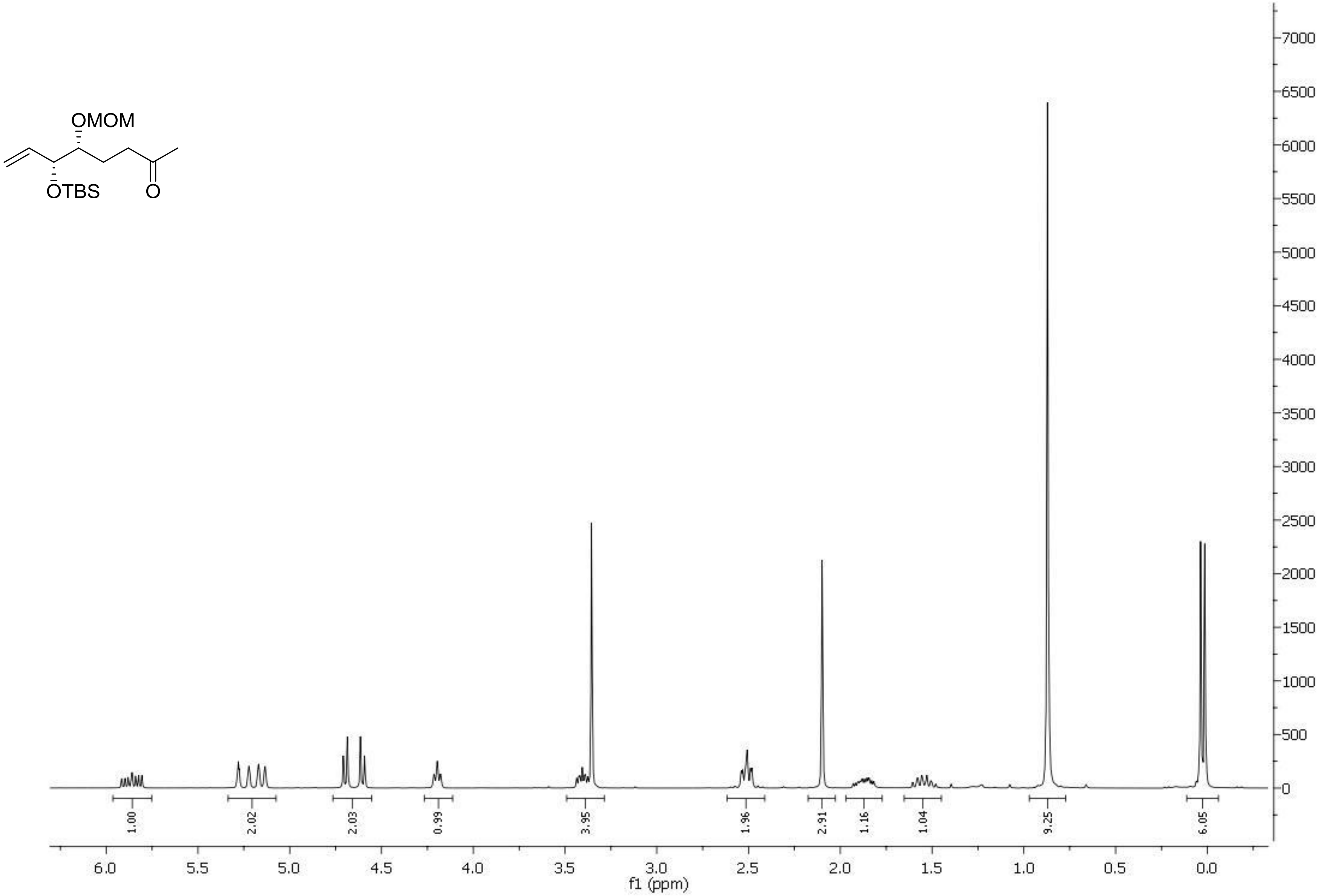

^{13}C NMR-APT (75 MHz, $CDCl_3$) of **13**

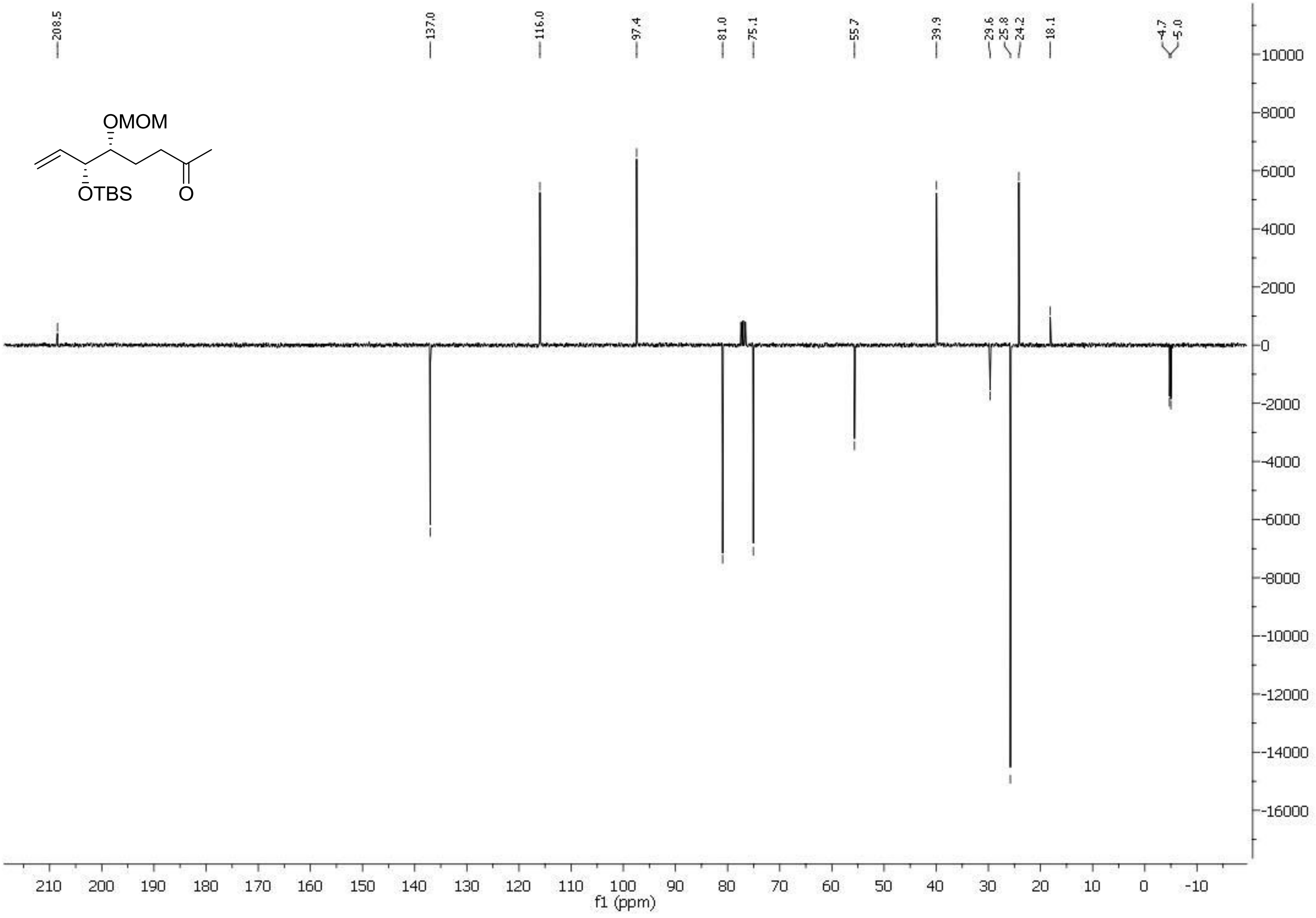

*B4 (3R,4R)-4-(Methoxymethoxy)-7-oxooct-1-en-3-yl but-3-enoate (**14**)*

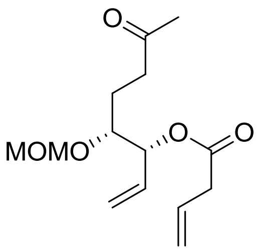

From ***13****:* To a solution of **13** (630 mg, 1.99 mmol) in THF (20 mL) was added tetrabutylammonium fluoride (0.75 g, 2.38 mmol) and the mixture was stirred for 3 h at 60 °C. The reaction was quenched by the addition of water and the aqueous layer was extracted three times with MTBE. The combined organic layers were dried with $MgSO_4$, filtered and evaporated. The residue was purified by column chromatography on silica (eluent: hexanes/MTBE 1:1). To a solution of the crude product (220 mg, 1.09 mmol) in dichloromethane (20 mL) was added vinylacetic acid (0.11 mL, 1.20 mmol), dicyclohexylcarbodiimide (247 mg, 1.20 mmol) and 4-*N*,*N*-dimethylaminopyridine (13 mg, 10 mol %) at 0 °C. The mixture was allowed to warm to room temperature and stirred for 16 h. The solution was filtered and washed three times with dichloromethane. The combined organic layers were washed with 1 M HCl (aq) solution and $NaHCO_3$ (aq) solution, dried with $MgSO_4$, filtered and evaporated. The residue was purified by column chromatography on silica (eluent: hexanes/MTBE 2:1) to give **14** (225 mg, 71% over two steps) as a colourless oil.

From ***16****:* $Cu(OAc)_2 \cdot H_2O$ (10 mg, 5.0 mol %) and BDP (4.5 mg, 1 mol %) were dissolved in dry, degassed toluene (3 mL). The mixture was stirred for 15 min at ambient temperature, and PMHS (0.14 mL, 2.0 mmol) was added. Stirring was continued for 0.5 h, after which time the colour changed from blue to green. A solution of **16** (268 mg, 1 mmol) in toluene (2.0 mL) was added and the solution was stirred at ambient temperature for 3 h. The reaction mixture was diluted with MTBE, tetrabutylammonium fluoride (631 mg, 2 mmol) was added and the mixture was stirred for 1 h. The solution was washed with a saturated aqueous $NaHCO_3$ solution, followed by aqueous HCl

(1 M). The aqueous layer was extracted twice with MTBE. The organic layers were dried with $MgSO_4$, filtered, and evaporated. The residue was purified by column chromatography on silica (eluent: hexanes/MTBE 2:1) to give **14** (236 mg, 87%) as a colourless oil: $[\alpha]^{26}_D$ = +51.6 (*c* 0.44, CH_2Cl_2); ^1H NMR (300 MHz, $CDCl_3$) δ 5.92 (ddt, *J* = 17.1, 9.8, 7.0, 1H), 5.82 (ddd, *J* = 17.0, 10.6, 6.3, 1H), 5.37-5.12 (5H), 4.68 (d, *J* = 6.8, 1H), 4.62 (d, *J* = 6.9, 1H), 3.63 (ddd, *J* = 8.2, 5.5, 4.3, 1H), 3.37 (s, 3H), 3.12 (dd, *J* = 7.0, 1.4, 2H), 2.55 (t, *J* = 7.0, 2H), 2.13 (s, 3H), 1.87 (m, 1H), 1.66 (m, 1H); ^{13}C NMR (75 MHz, $CDCl_3$) δ 207.9 (0), 170.4 (0), 132.5 (1), 130.0 (1), 118.7 (2), 118.7 (2), 97.0 (2), 77.9 (1), 75.6 (1), 55.9 (3), 39.2, 39.0 (2), 29.9 (3), 24.5 (2); IR (neat) *v* 2935 (w), 1737 (s), 1716 (s), 1414 (w), 1360 (m), 1167 (s), 1149 (s); MS (ESI) *m/z* 153 (10), 239 (8), 293 ($[M+Na]^+$, 100); HRMS (ESI) calcd. for $C_{14}H_{22}O_5Na^+$ ($[M+Na]^+$) 293.1365, found 293.1377; Anal. Calcd for $C_{14}H_{22}O_5$: C, 62.2; H, 8.2; Found: C, 62.2; H, 8.4.

^{1}H NMR (300 MHz, $CDCl_3$) of **14**

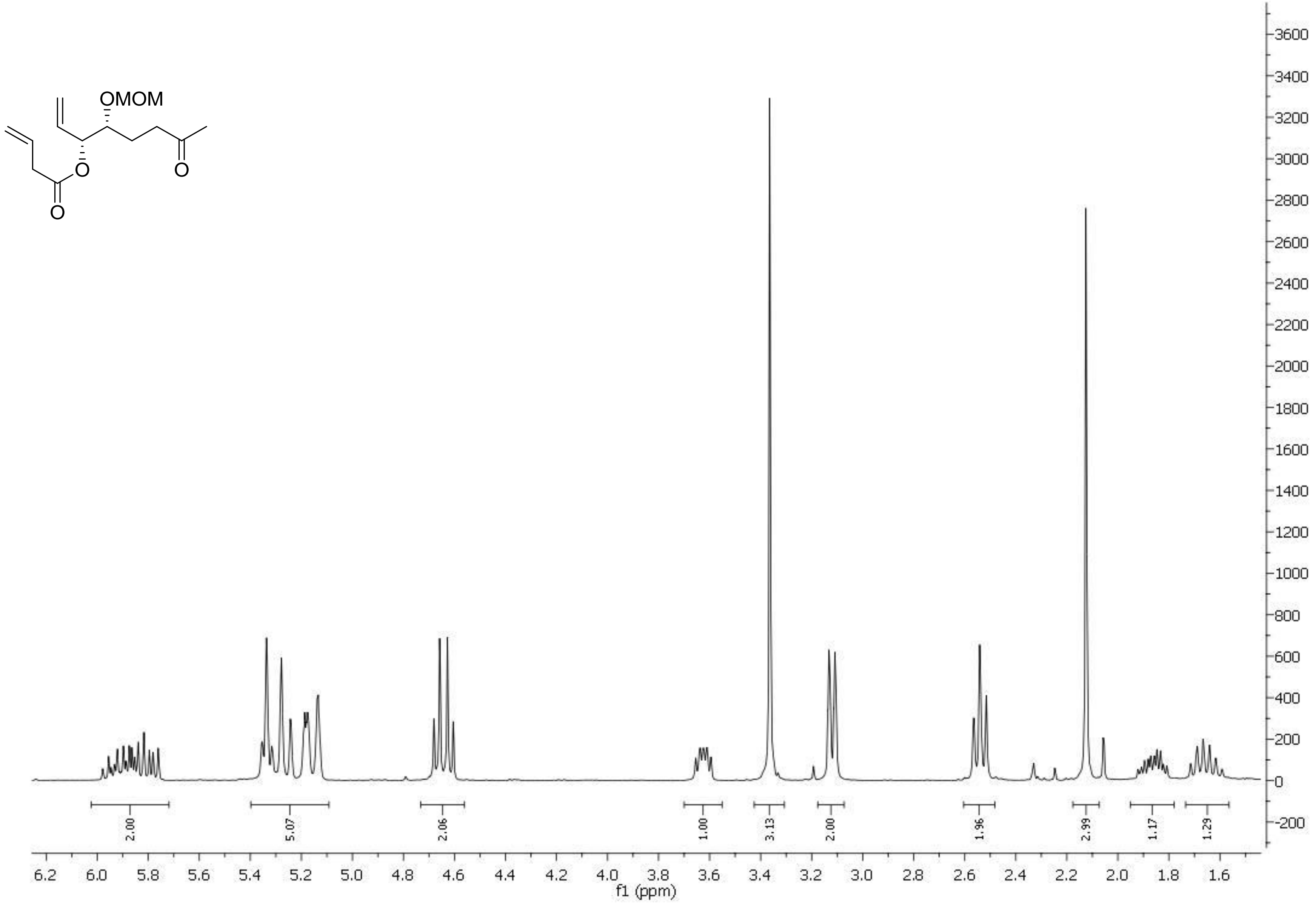

^{13}C NMR-APT (75 MHz, $CDCl_3$) of **14**

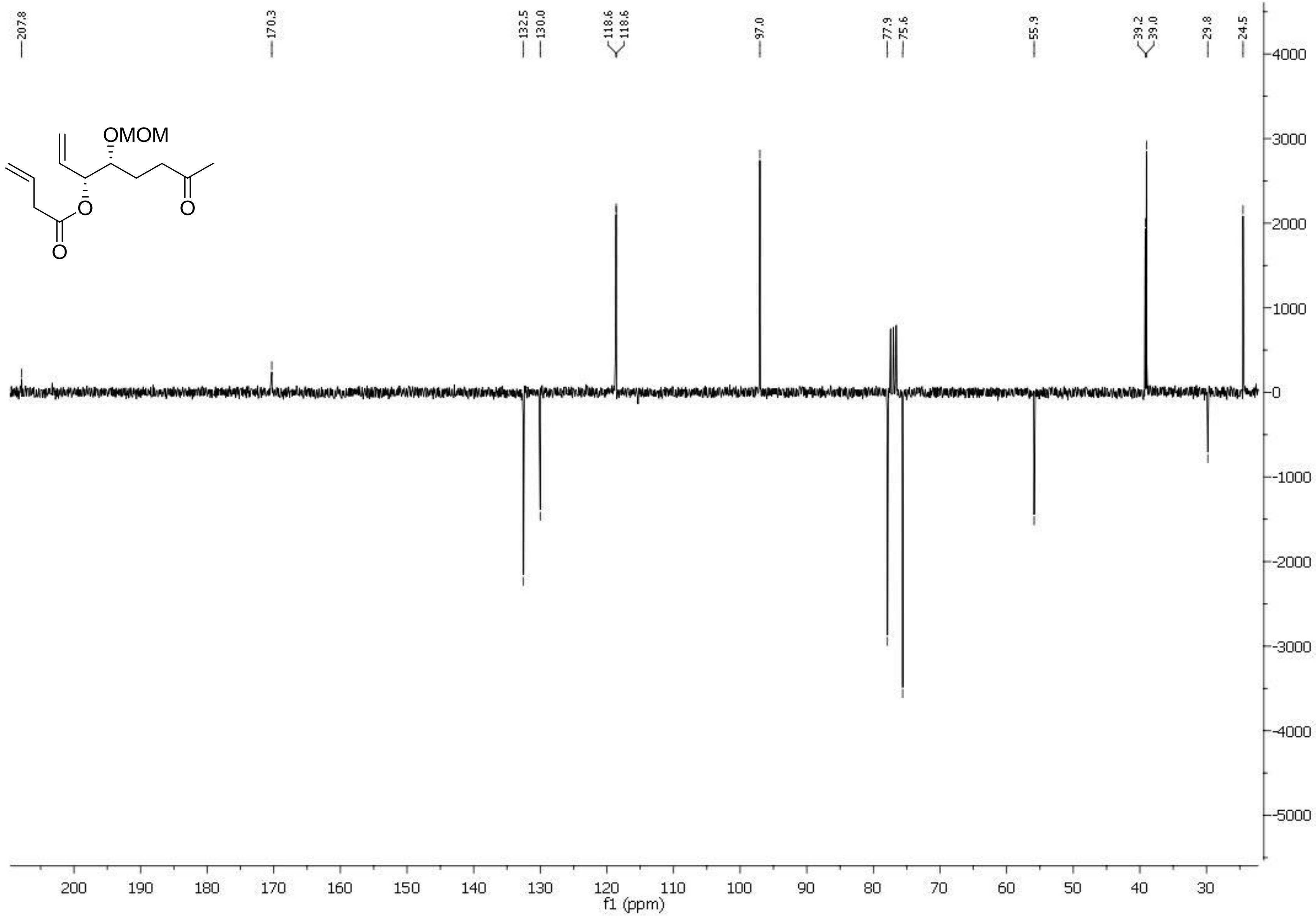

*B5 (5R,6R,E)-6-Hydroxy-5-(methoxymethoxy)octa-3,7-dien-2-one (**15**)*

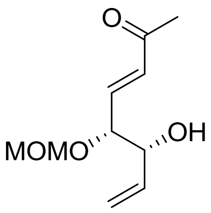

To a solution of **12** (160 mg, 0.51 mmol) in THF (30 mL) was added tetrabutylammonium fluoride (181 mg, 0.57 mmol) and the mixture was stirred for 15 min at room temperature. The reaction was quenched by the addition of water and the aqueous layer was extracted three times with MTBE. The combined organic layers were dried with $MgSO_4$, filtered and evaporated. The residue was purified by column chromatography on silica (eluent: hexanes/MTBE 3:1) to give **15** (99 mg, 97%) as a colourless oil: $[\alpha]^{26}_D = -45.9$ (*c* 0.40, CH_2Cl_2); ^1H NMR (300 MHz, $CDCl_3$) δ 6.65 (dd, J = 16.1, 6.1, 1H), 6.25 (d, J = 16.1, 1H), 5.84 (ddd, J = 17.1, 10.5, 5.7, 1H), 5.36 (d, J = 17.2, 1H), 5.25 (d, J = 10.5, 1H), 4.66 (s, 2H, 4.17-4.12 (2H), 3.39 (s, 3H), 2.78 (bs, 1H), 2.26 (s, 3H); ^{13}C NMR (75 MHz, $CDCl_3$) δ 197.6 (0), 142.5 (1), 136.0 (1), 132.6 (1), 117.6 (2), 95.5 (2), 79.4 (1), 74.5 (1), 56.0 (3), 27.3 (3); IR (neat) ν 3442 (w), 2894 (w), 1674 (s), 1632 (m), 1361 (m), 1257 (s); MS (ESI) *m/z* 122 (20), 139 (10), 223 ($[M+Na]^+$, 100); HRMS (ESI) calcd for $C_{10}H_{16}O_4Na^+$ ($[M+Na]^+$) 223.0946, found 223.0938; Anal. Calcd for $C_{10}H_{16}O_4$: C, 60.0; H, 8.1; Found: C, 59.9; H, 8.1.

^{1}H NMR (300 MHz, $CDCl_3$) of **15**

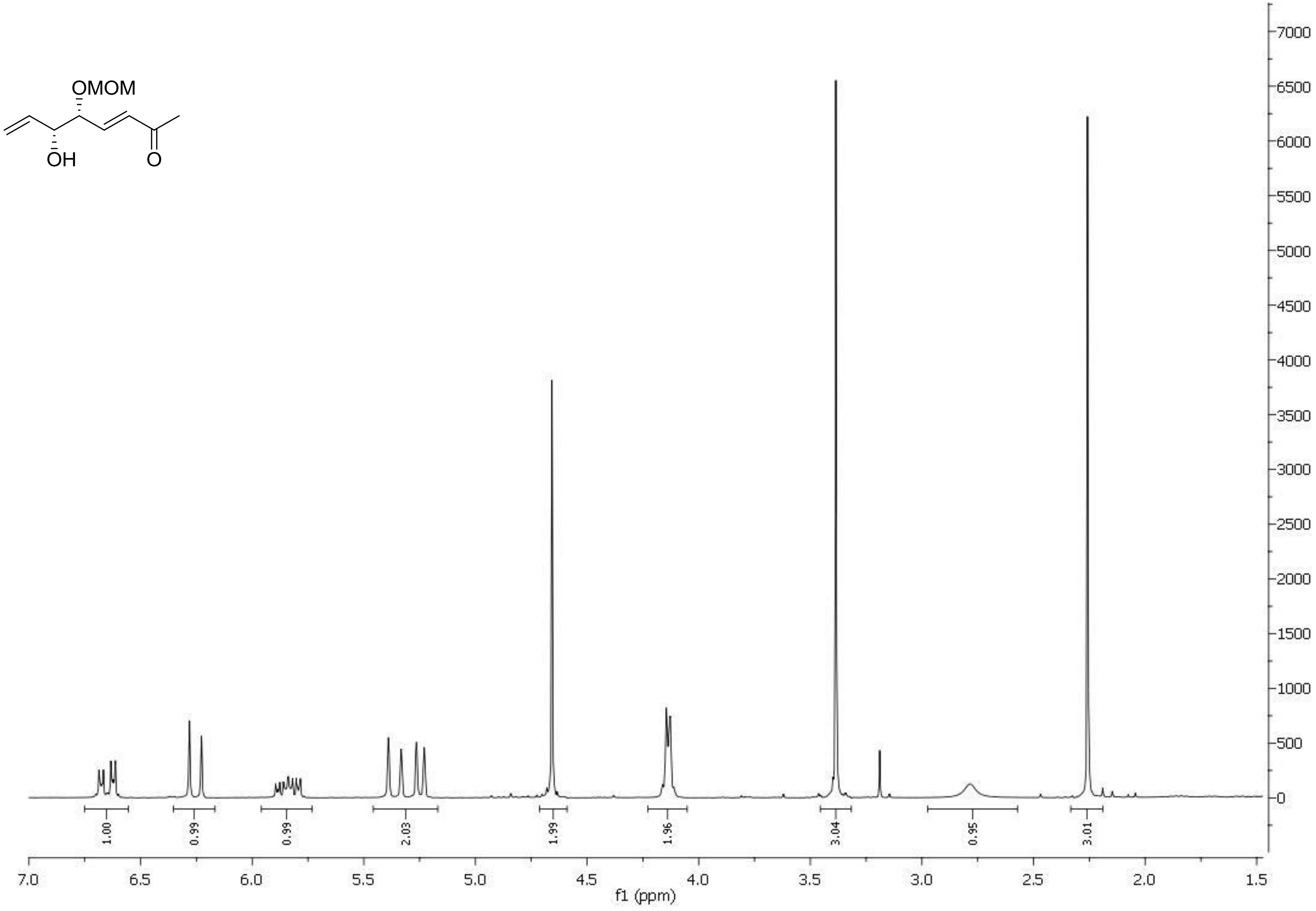

^{13}C NMR-APT (75 MHz, $CDCl_3$) of **15**

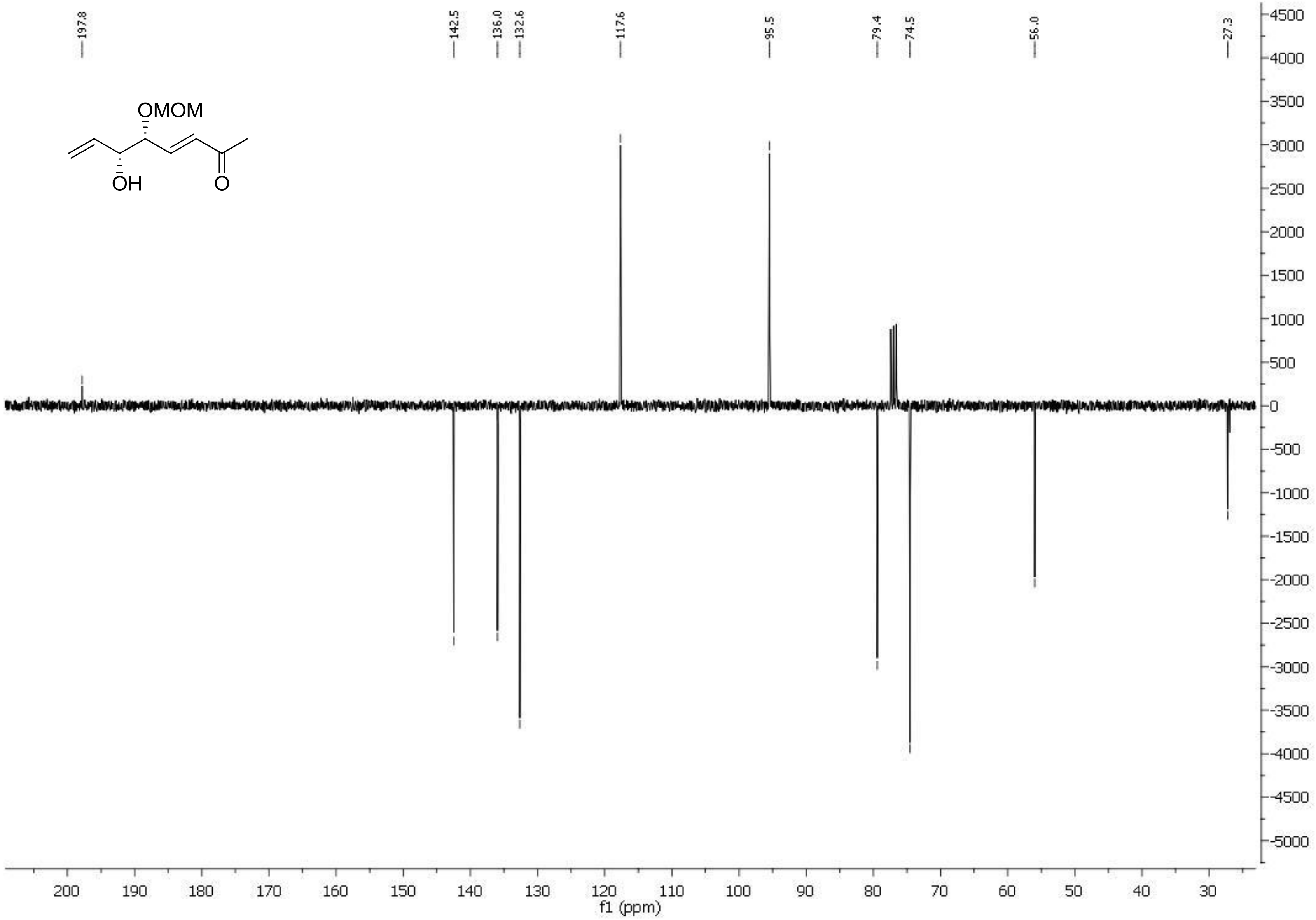

B6 *(3R,4R,E)-4-(Methoxymethoxy)-7-oxoocta-1,5-dien-3-yl but-3-enoate (**16**)*

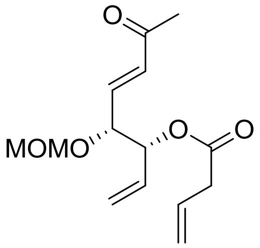

To a solution of allylic alcohol **15** (1.9 g, 9.5 mmol) in dichloromethane (95 mL) was added vinylacetic acid (0.89 mL, 10.5 mmol), dicyclohexylcarbodiimide (2.15 g, 10.5 mmol) and 4-*N*,*N*-dimethylaminopyridine (128 mg, 10 mol %) at 0 °C. The mixture was allowed to warm to room temperature and stirred for 1 h. The solution was filtered and washed three times with dichloromethane. The combined organic layers were washed with 1 M HCl (aq) solution and $NaHCO_3$ (aq) solution, dried with $MgSO_4$, filtered and evaporated. The residue was purified by column chromatography on silica (eluent: hexanes/MTBE 2:1) to give **16** (2.28 g, 90%) as a colourless oil: $[\alpha]^{26}_{D}$ = –3.3 (*c* 0.55, CH_2Cl_2); ^{1}H NMR (300 MHz, $CDCl_3$) δ 6.57 (dd, *J* = 16.1, 5.9, 1H), 6.25 (d, *J* = 16.1, 1H), 5.87 (ddt, *J* = 17.2, 10.0, 6.9, 1H), 5.78 (ddd, *J* = 17.0, 10.5, 6.2, 1H), 5.41 (dd, *J* = 6.0, 5.7, 1H), 5.35-5.10 (4H), 4.64 (d, *J* = 6.9, 1H), 4.61 (d, *J* = 6.9, 1H), 4.35 (dd, *J* = 5.7, 5.6, 1H), 3.35 (s, 3H), 3.10 (d, *J* = 7.0, 2H), 2.23 (s, 3H); ^{13}C NMR (75 MHz, $CDCl_3$) δ 197.5 (0), 170.1 (0), 141.5 (1), 132.6 (1), 131.9 (1), 129.7 (1), 119.2 (2), 118.8 (2), 95.2 (2), 76.0 (1), 74.9 (1), 55.8 (3), 39.0 (2), 27.3 (3); IR (neat) *ν* 3084 (w), 2937 (w), 1739 (s), 1678 (s), 1423 (w), 1359 (m), 1250 (s), 1156 (s); MS (ESI) *m/z* 291 ($[M+Na]^+$, 100); HRMS (ESI) calcd for $C_{14}H_{20}O_5Na^+$ ($[M+Na]^+$) 291.1208, found 291.1220; Anal. Calcd for $C_{14}H_{20}O_5$: C, 62.7; H, 7.5; Found: C, 62.5; H, 7.5.

^{1}H NMR (300 MHz, $CDCl_3$) of **16**

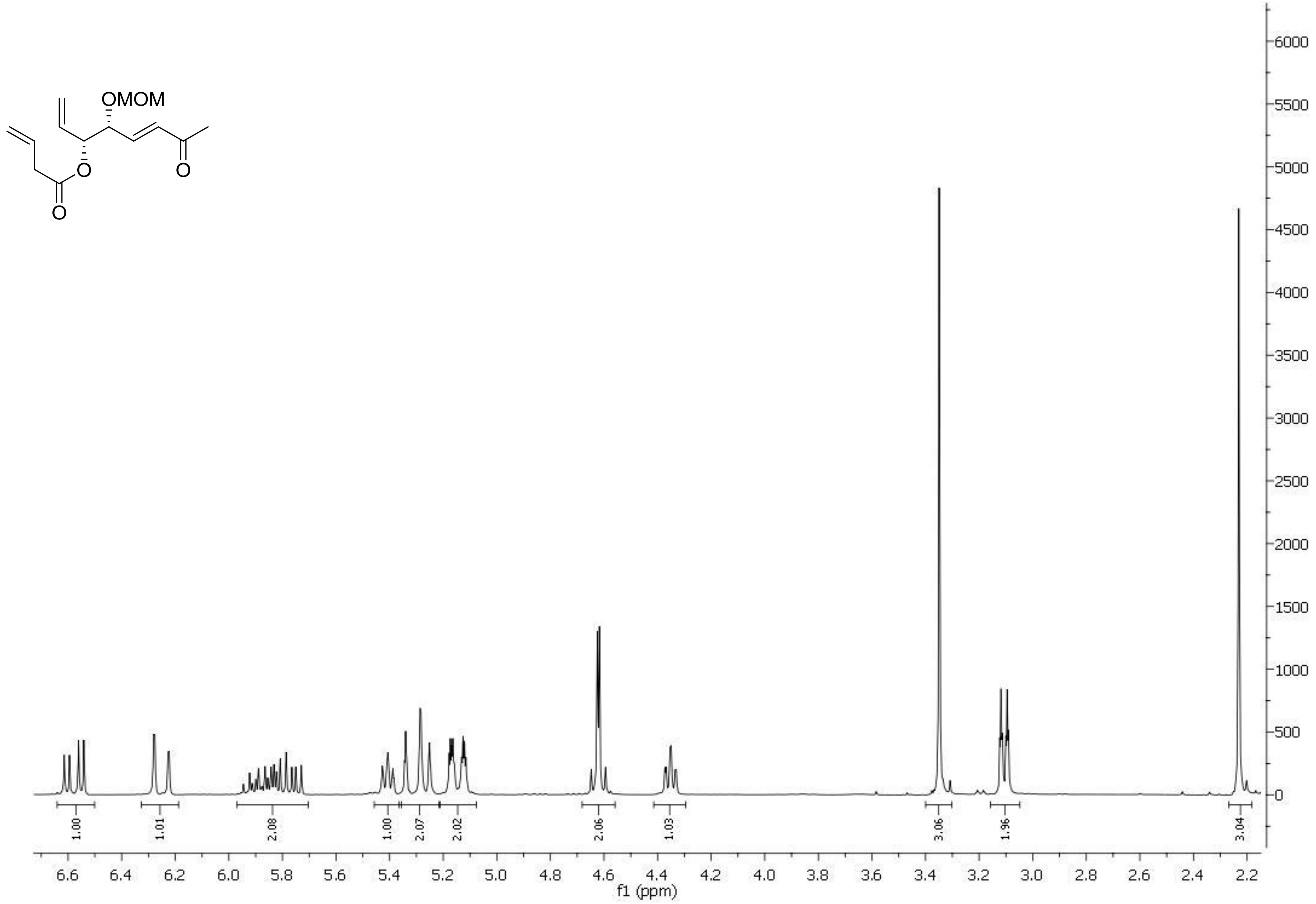

^{13}C NMR-APT (75 MHz, $CDCl_3$) of **16**

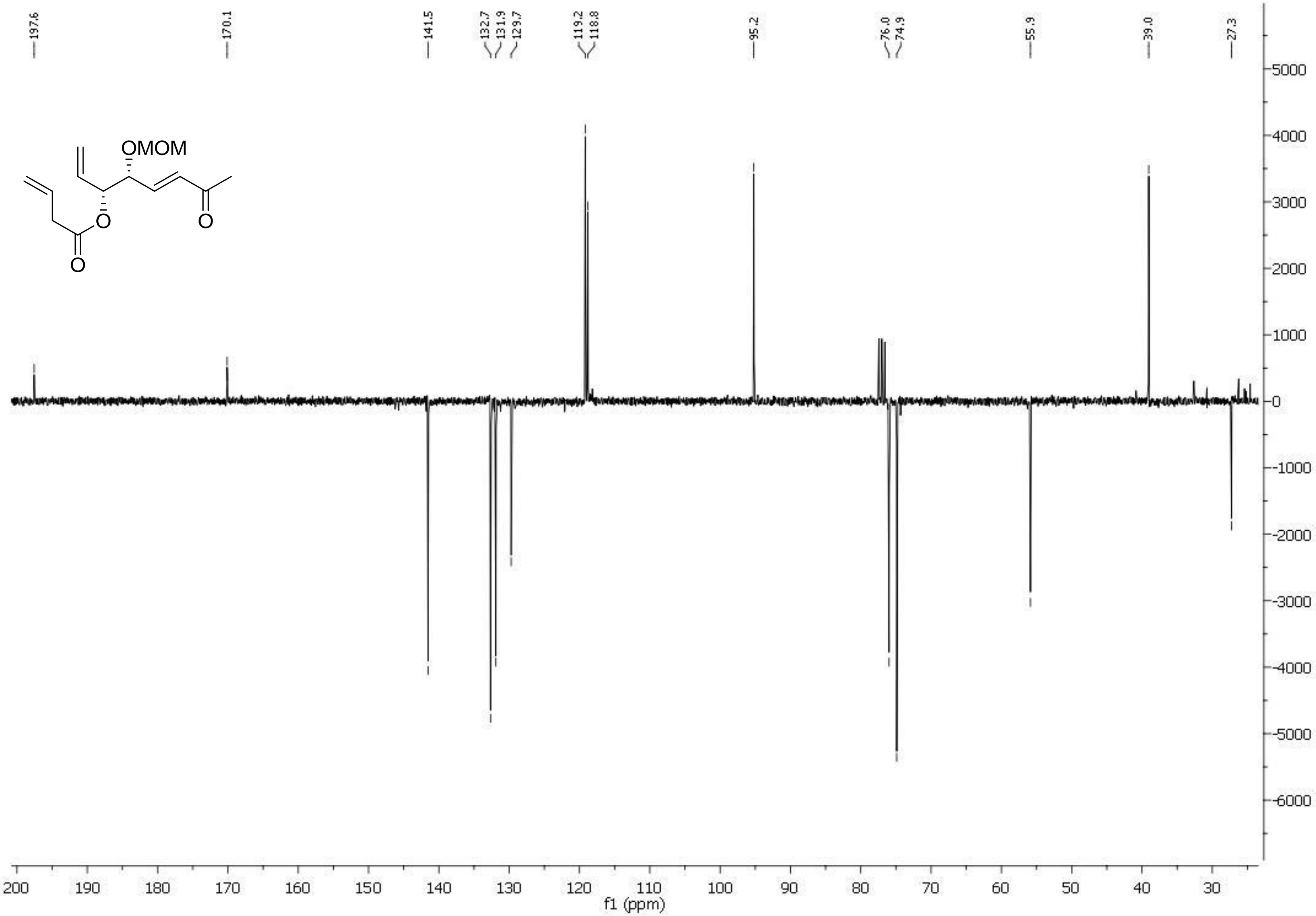

*B7 (3R,4R)-7-Hydroxy-4-(methoxymethoxy)oct-1-en-3-yl but-3-enoate (**18**)*

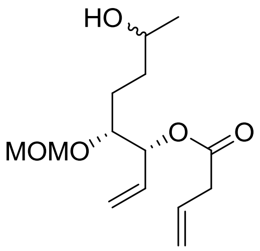

To a solution of **14** (590 mg, 2.20 mmol) in methanol (10 mL) was added sodium borohydride (81 mg, 2.20 mmol) at ambient temperature. The mixture was stirred for 30 min at ambient temperature, and water was added. The reaction mixture was diluted with MTBE, and the aqueous layer was extracted twice with MTBE. The organic layers were dried with $MgSO_4$, filtered, and evaporated. The residue was purified by column chromatography on silica (eluent: hexanes/MTBE 1:1) to give **18** (452 mg, 76%) as a colourless oil and as an inseparable mixture of two diastereomers: ^{1}H NMR (300 MHz, $CDCl_3$) δ 6.00-5.75 (2H), 5.40-5.12 (5H), 4.75-4.65 (2H), 3.78 (m, 1H), 3.67 (m, 1H), 3.40-3.37 (3H), 3.14 (d, J = 6.9, 2H), 1.72-1.47 (5H), 1.17 (d, J = 6.2, 3H); ^{13}C NMR (75 MHz, $CDCl_3$) δ 170.4 (0), 132.8 (1), 130.1 (1), 118.6 (2), 118.4 (2), 118.4 (2), 97.0 (2), 96.9 (2), 78.7 (1), 78.5 (1), 75.7 (1), 75.6 (1), 68.0 (1), 67.8 (1), 55.9 (3), 39.3 (2), 27.1 (2), 26.7 (2), 23.7 (3), 23.5 (3).

^{1}H NMR (300 MHz, $CDCl_3$) of **18**

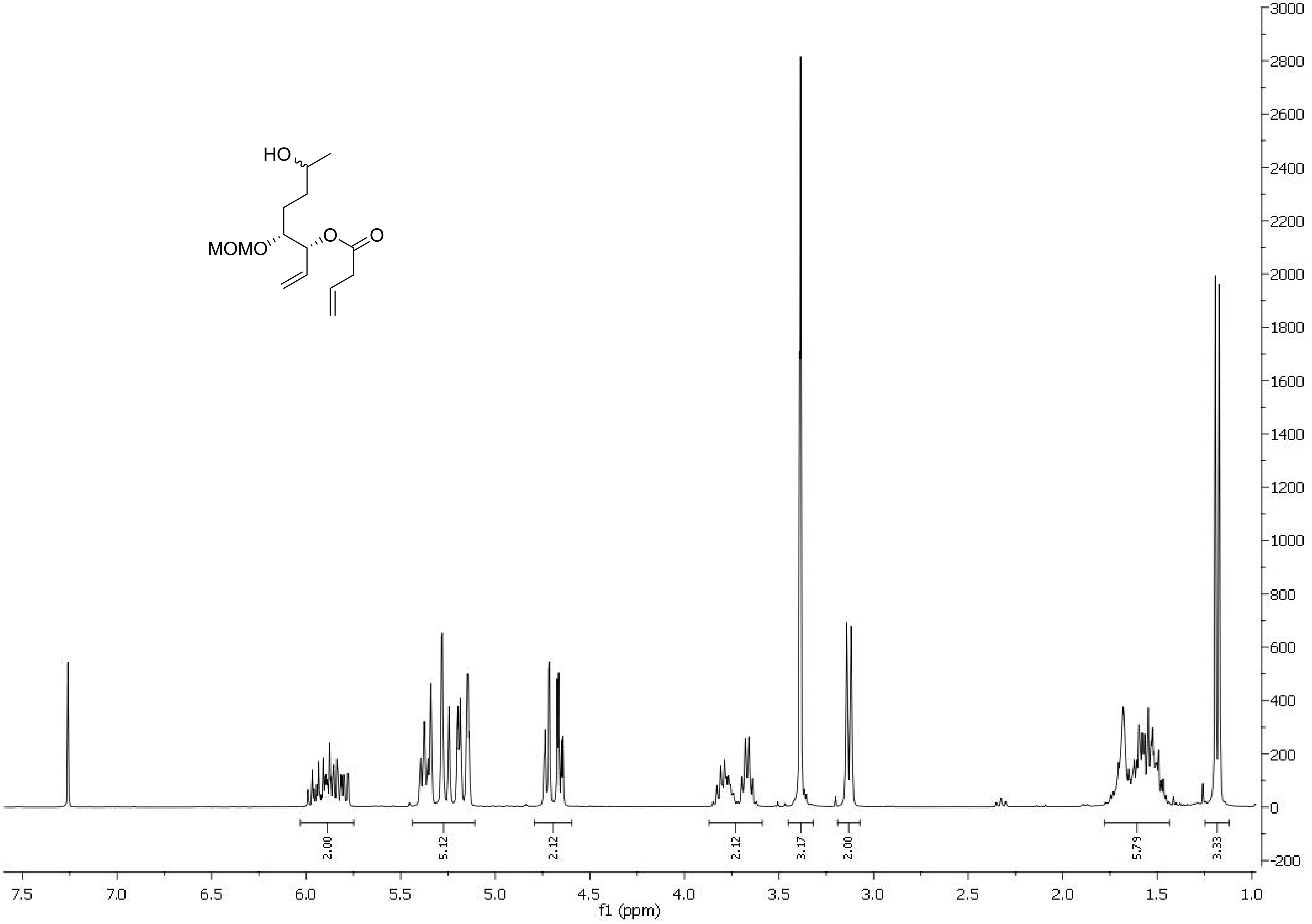

^{13}C NMR-APT (75 MHz, $CDCl_3$) of **18**

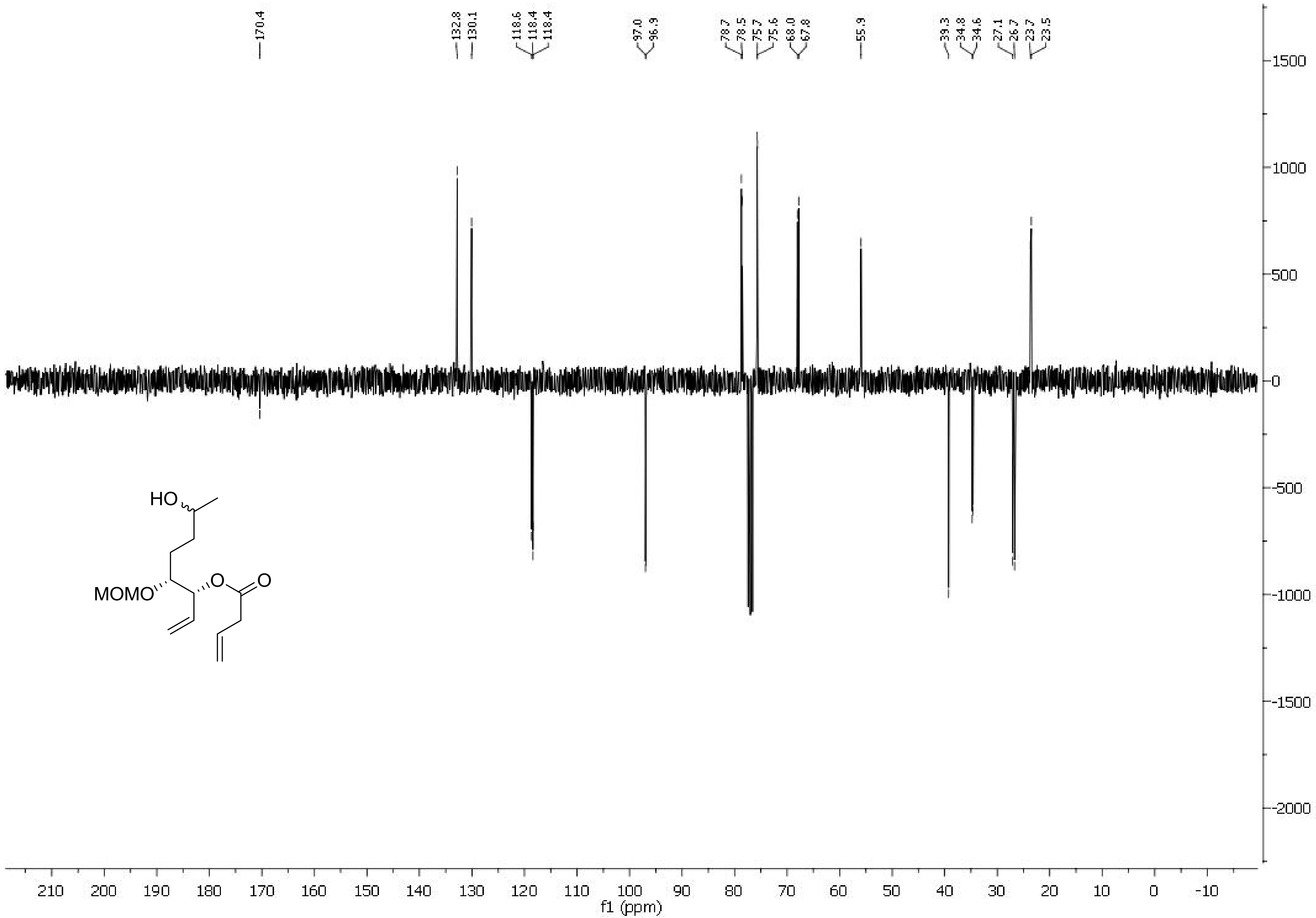

*B8 (R,2Z,4E)-9-Hydroxy-6-(methoxymethoxy)deca-2,4-dienoic acid (**19**)*

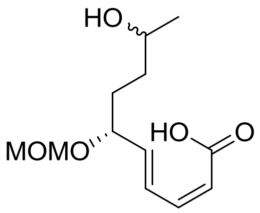

To a solution of **20** (100 mg, 0.41 mmol) in THF (4 mL) was added DIBAL-H (1.1 M solution in cyclohexane, 0.79 mL, 0.86 mmol) at 0 °C. The solution was stirred for 30 minutes at this temperature and afterwards allowed to warm to room temperature. After this time, tartaric acid (aq) solution was added and the mixture was stirred for 1 hour. The aqueous layer was extracted three times with MTBE and the combined organic layers were dried with $MgSO_4$, filtered and evaporated. The residue was purified by column chromatography on silica (eluent: hexanes/MTBE 1:1) to give **20** (51 mg, 51%) as a colourless oil and as an inseparable mixture of two diastereomers. ^{1}H NMR (300 MHz, $CDCl_3$) δ 7.46 (dd, J = 15.3, 11.6, 1H), 6.64 (dd, J = 11.5, 11.5, 1H), 5.94 (dd, J = 15.4, 6.9, 1H), 5.68 (d, J = 11.4, 1H), 4.70-4.55 (2H), 4.21 (m, 1H), 3.82 (m, 1H), 3.38 (s, 3H), 1.75-1.45 (4H), 1.19 (d, J = 6.2, 3H); ^{13}C NMR (75 MHz, $CDCl_3$) δ 170.3 (0), 145.5 (1), 144.0 (1), 128.0 (1), 117.5 (1), 94.5 (2), 94.5 (2), 76.5 (1), 76.4 (1), 68.0 (1), 67.9 (1), 55.6 (3), 34.8 (2), 34.7 (2), 31.6 (2), 31.5 (2), 23.5 (3), 23.4 (3); IR (neat) ν 2934 (w), 1734 (s), 1646 (w), 1447 (w), 1372 (m), 1243 (s), 1034 (s); HRMS (ESI) calcd. for $C_{12}H_{21}O_5^+$ ($[M+H]^+$) 245.1389, found 245.1382; Anal. calcd. for $C_{12}H_{20}O_5$: C, 59.0; H, 8.3; Found: C, 59.0; H, 8.4.

^{1}H NMR (300 MHz, $CDCl_3$) of **19**

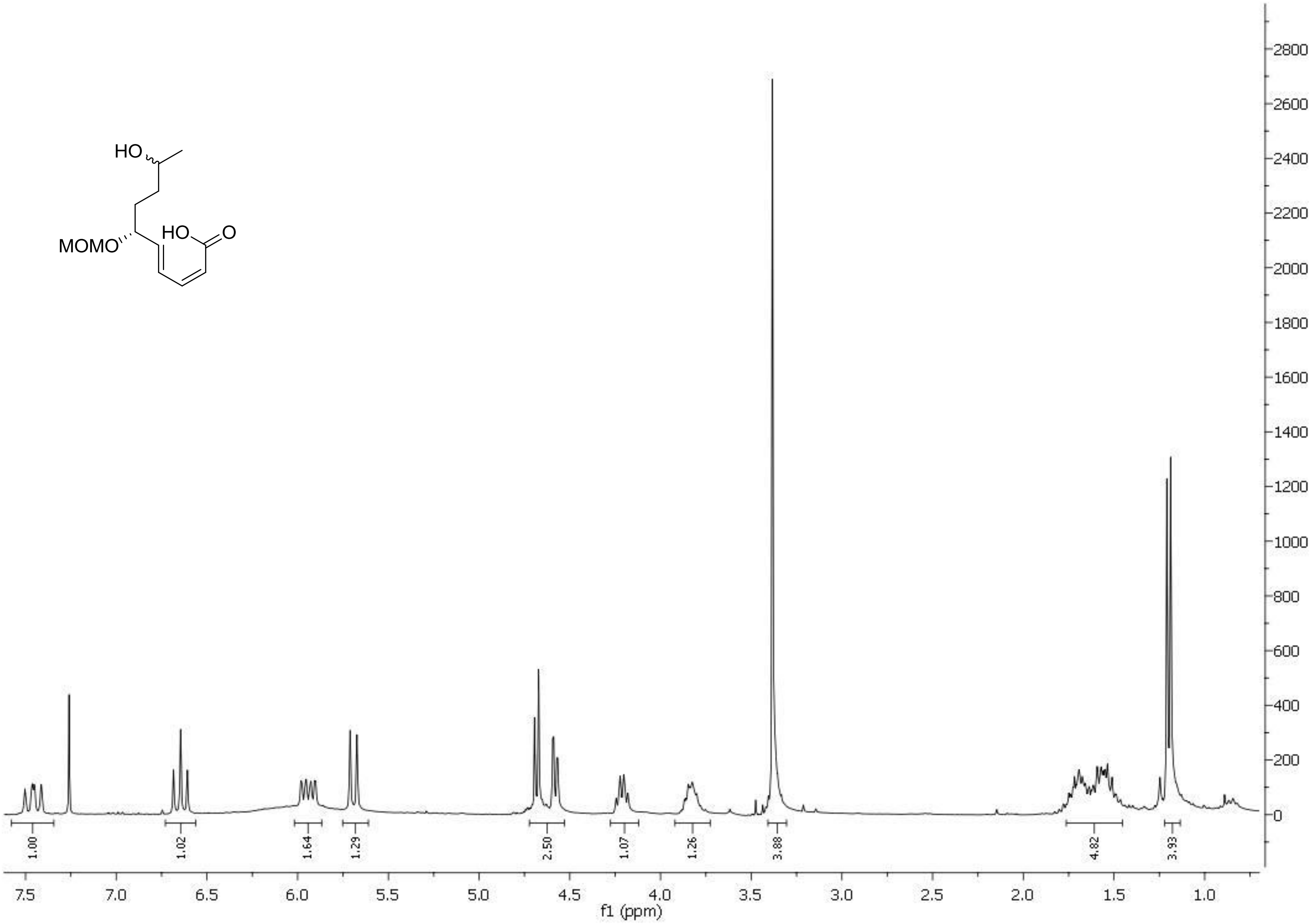

^{13}C NMR-APT (75 MHz, $CDCl_3$) of **19**

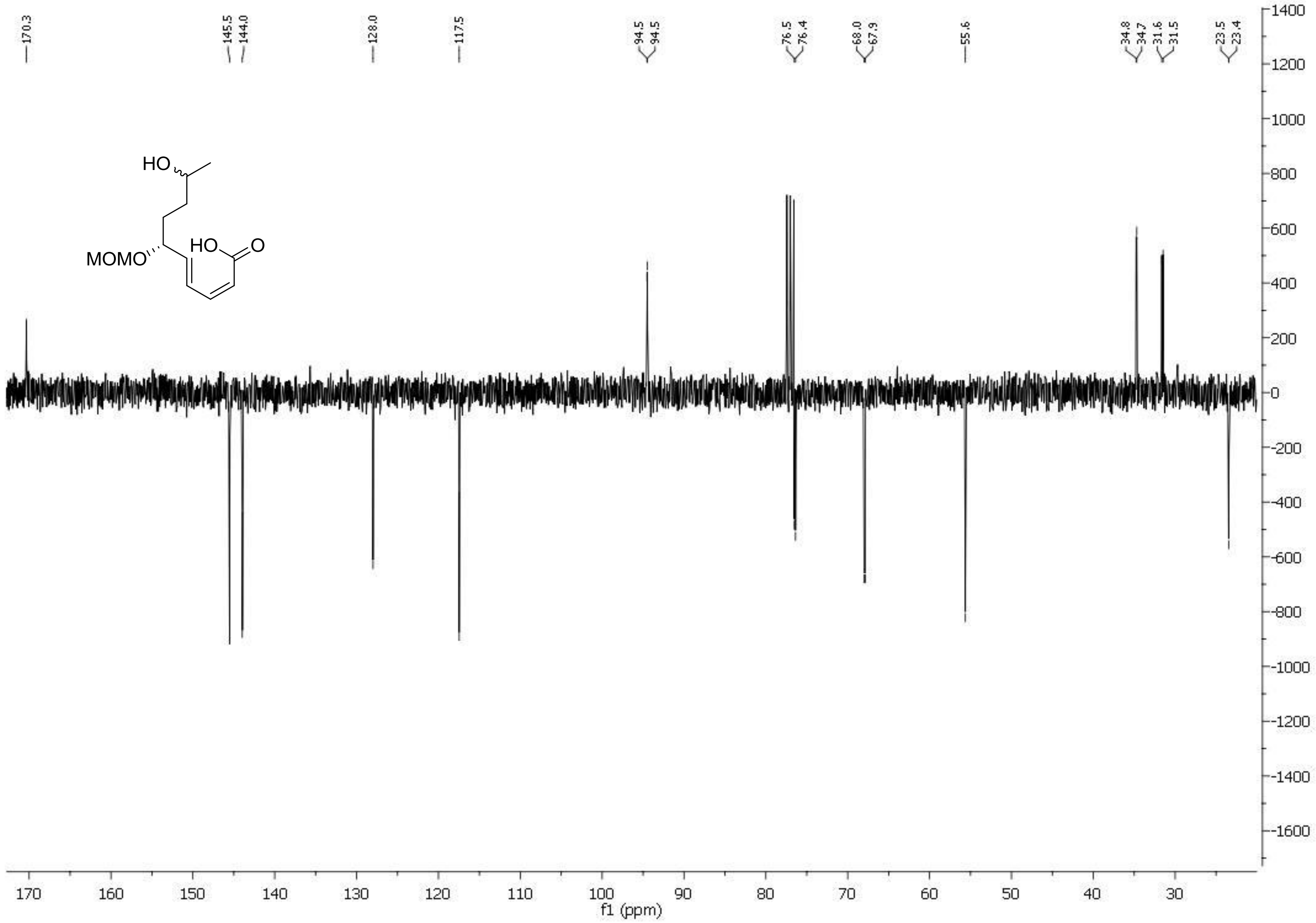

*B9 (R,2Z,4E)-6-(Methoxymethoxy)-9-oxodeca-2,4-dienoic acid (**20**)*

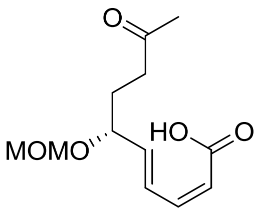

To a solution of **14** (400 mg, 1.48 mmol) in toluene (15 mL) was added Ru catalyst **B** (25 mg, 2 mol %) at 80 °C. The mixture was stirred for 30 min and sodium hydride (60 wt % in mineral oil, 89 mg, 2.22 mmol) was added. The mixture was stirred at 80 °C for 1 h and was cooled to ambient temperature. The reaction was quenched by the addition of water and acidified with 1 M HCl. The aqueous layer was extracted three times with methyl *tert*-butyl ether (MTBE) and the combined organic layers were dried with $MgSO_4$, filtered and evaporated. The residue was purified by column chromatography on silica (eluent: hexanes/MTBE 1:1) to give **20** (230 mg, 64%) as a colourless oil: ^{1}H NMR (300 MHz, $CDCl_3$) δ 7.45 (dd, J = 15.5, 11.4, 1H), 6.62 (dd, J = 11.4, 11.4, 1H), 5.89 (dd, J = 15.5, 7.3, 1H), 5.63 (d, J = 11.4, 1H), 4.63 (d, J = 6.7, 1H), 4.54 (d, J = 6.8, 1H), 4.17 (dt, J = 6.7, 6.5, 1H), 3.35 (s, 3H), 2.52 (dt, J = 7.2, 1.7, 2H), 2.13 (s, 3H), 1.85 (dt, J = 7.2, 6.7, 2H); ^{13}C NMR (75 MHz, $CDCl_3$) δ 208.0 (0), 170.7 (0), 145.3 (1), 143.5 (1), 128.0 (1), 117.6 (1), 94.5 (2), 75.5 (1), 55.5 (3), 39.0 (2), 29.8 (3), 29.0 (2); IR (neat) v 3053 (w), 2934 (w), 1709 (s), 1640 (m), 1601 (m), 1359 (m), 1148 (s), 1026 (s); MS (ESI) *m/z* 122 (30), 181 (20), 265 ([M+Na]$^+$, 100); HRMS (ESI) calcd. for $C_{12}H_{18}O_5Na^+$ ([M+Na]$^+$) 265.1052, found 265.1052; Anal. calcd. for $C_{12}H_{18}O_5$: C, 59.5; H, 7.5; Found C, 59.6; H, 7.7.

^{1}H NMR (300 MHz, $CDCl_3$) of **20**

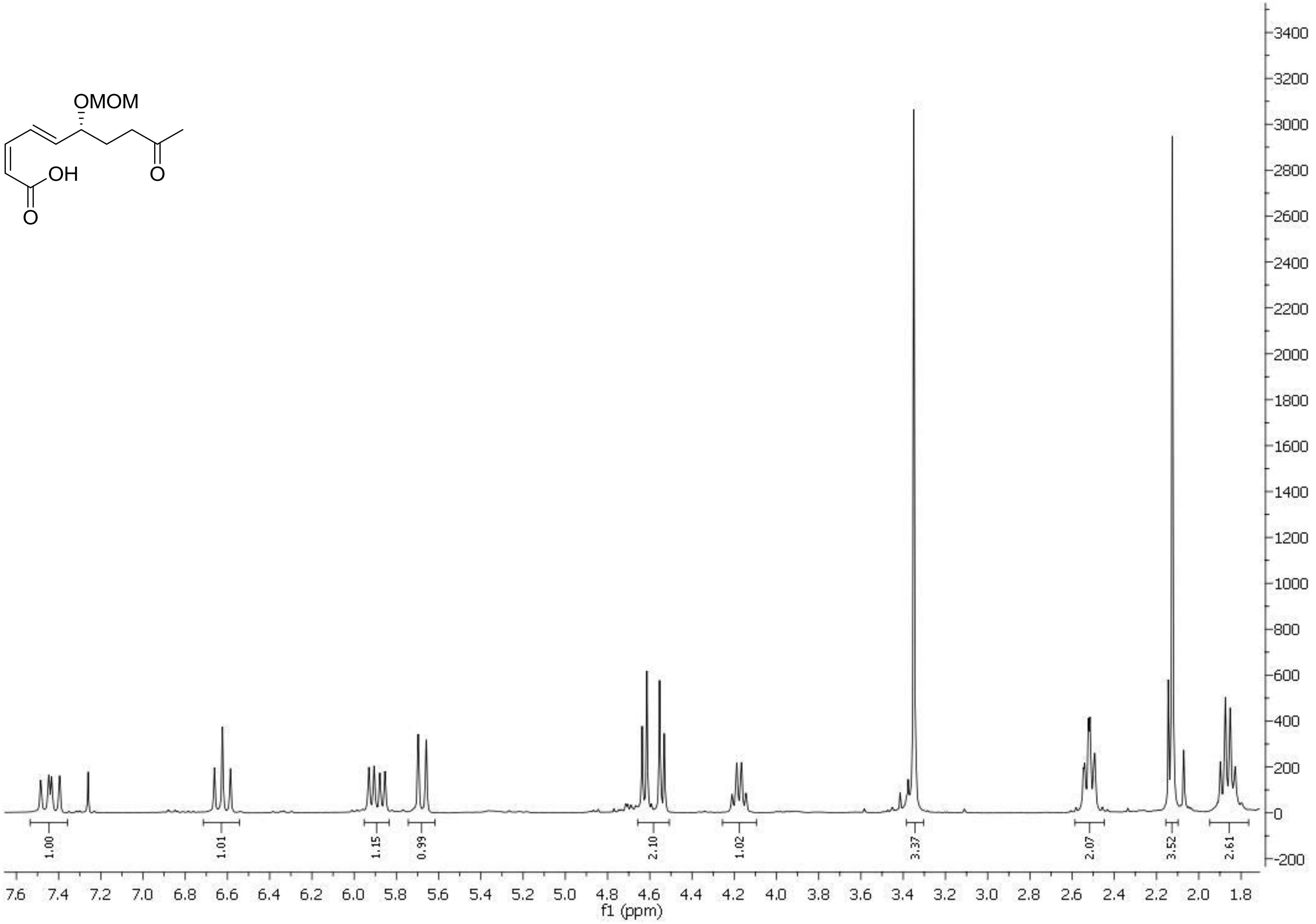

^{13}C NMR-APT (75 MHz, $CDCl_3$) of **20**

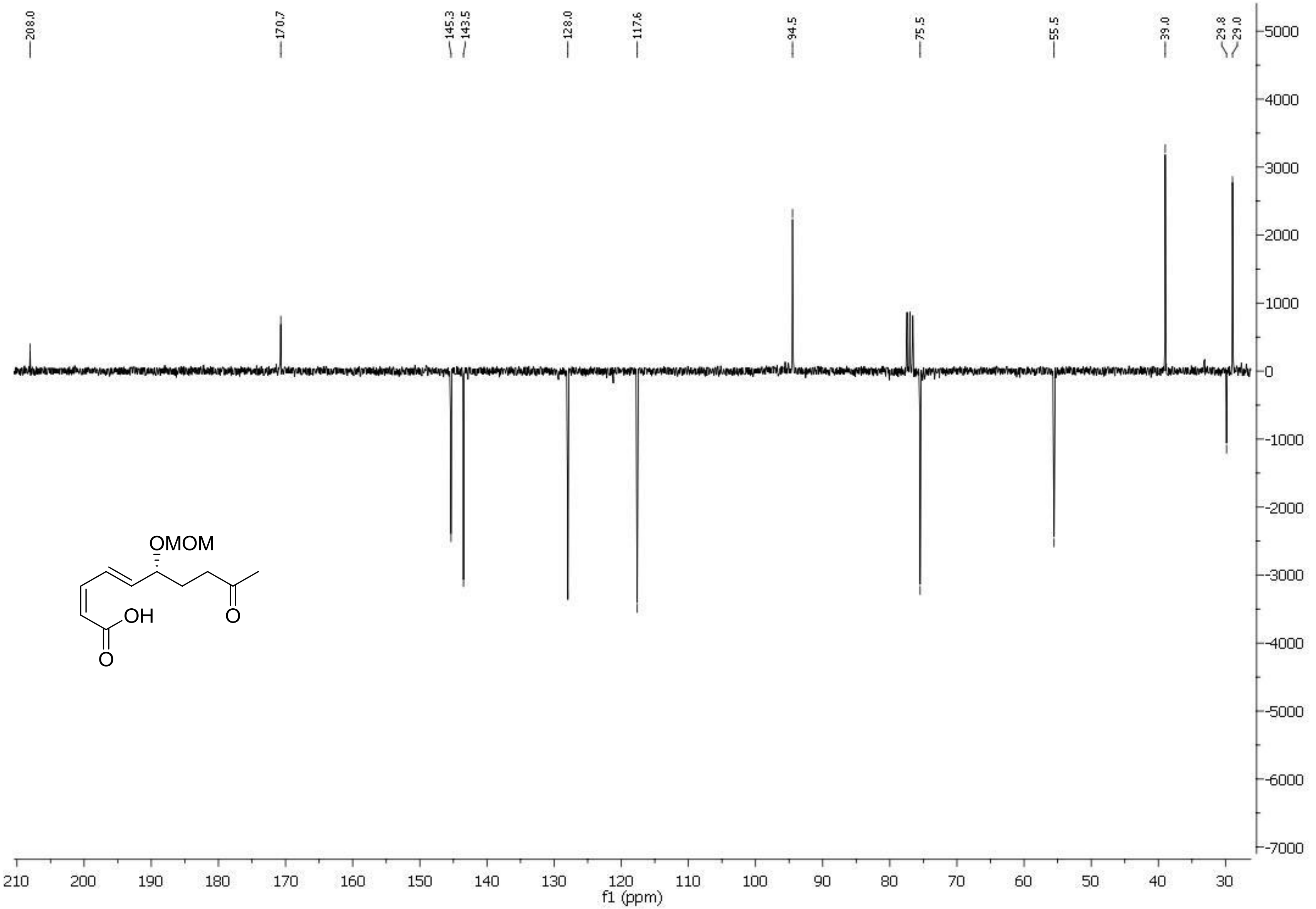

*B10 (5R,6R)-6-(tert-Butyldimethylsilyloxy)-5-(methoxymethoxy)oct-7-en-2-ol(**21**)*

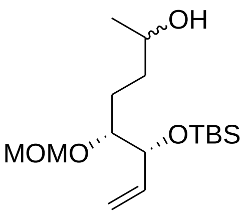

*From ketone **13***: To a solution of **13** (120 mg, 0.38 mmol) in methanol (10 mL) was added sodium borohydride (14 mg, 0.38 mmol) at ambient temperature. The mixture was stirred for 30 min at this temperature, and water was added. The reaction mixture was diluted with MTBE, and the aqueous layer was extracted twice with MTBE. The organic layers were dried with $MgSO_4$, filtered, and evaporated. The residue was purified by column chromatography on silica (eluent: hexanes/MTBE 3:1) to give **21** (110 mg, 92%) as a colourless oil and as an inseparable mixture of two diastereomers:

*From ester **25***: To a solution of **25** (1.0 g, 3.0 mmol) in dichloromethane (30 mL) was added DIBAL-H (1.1 M solution in cyclohexane, 2.9 mL, 3.2 mmol) at −78 °C. The solution was stirred for 20 minutes at this temperature and methylmagnesium chloride (3.0 M solution in THF, 3.0 mL, 9.0 mmol) was added. Stirring at −78 °C was continued for another 20 min and the mixture was allowed to warm to room temperature. After this time, tartaric acid (aq) solution was added and the mixture was stirred for 1 hour. The aqueous layer was extracted three times with MTBE and the combined organic layers were dried with $MgSO_4$, filtered and evaporated. The residue was purified by column chromatography on silica (eluent: hexanes/MTBE 5:1) to give **25** (903 mg, 95%) as a colourless oil and as an inseparable mixture of two diastereomers. ^{1}H NMR (300 MHz, $CDCl_3$) δ 5.86 (m, 1H), 5.24 (dd, J = 17.2, 1.7, 1H), 5.14 (dd, J = 10.5, 1.7, 1H), 4.75 (m, 1H), 4.63 (m, 1H), 4.22 (ddm, J = 5.5, 5.5, 1H), 3.75 (m, 1H), 3.44 (m, 1H), 3.37 (s, 3H), 2.01 (bs, 1H), 1.75- 1.30 (4H), 1.15 (d, J = 6.2, 3H), 0.87 (s, 9H), 0.04 (s, 3H), 0.02 (s, 3H); ^{13}C NMR (75 MHz, $CDCl_3$) δ 137.3 (1), 115.8 (2), 115.8 (2), 97.4 (2), 97.3 (2), 81.9 (1), 81.8 (1), 75.0 (1), 74.9 (1), 68.1 (1), 67.9 (1), 55.7 (3), 35.5 (2), 35.4 (2), 26.3 (2), 26.0 (2), 25.8 (3), 23.4 (3), 18.1 (0), −4.7 (3), −4.9 (3); IR

(neat) *v* 3417 (w), 2955 (w), 2930 (w), 2857 (w), 1466 (w), 1252 (w), 1032 (s); MS (ESI) *m/z* 319 ($[M+H]^+$, 5); HRMS (ESI) calcd for $C_{16}H_{35}O_4Si^+$ ($[M+H]^+$) 319.2305, found 319.2324; Anal. Calcd for $C_{16}H_{34}O_4Si$: C, 60.3; H, 10.8; Found: C, 59.9; H, 10.9.

^{1}H NMR (300 MHz, $CDCl_3$) of **21**

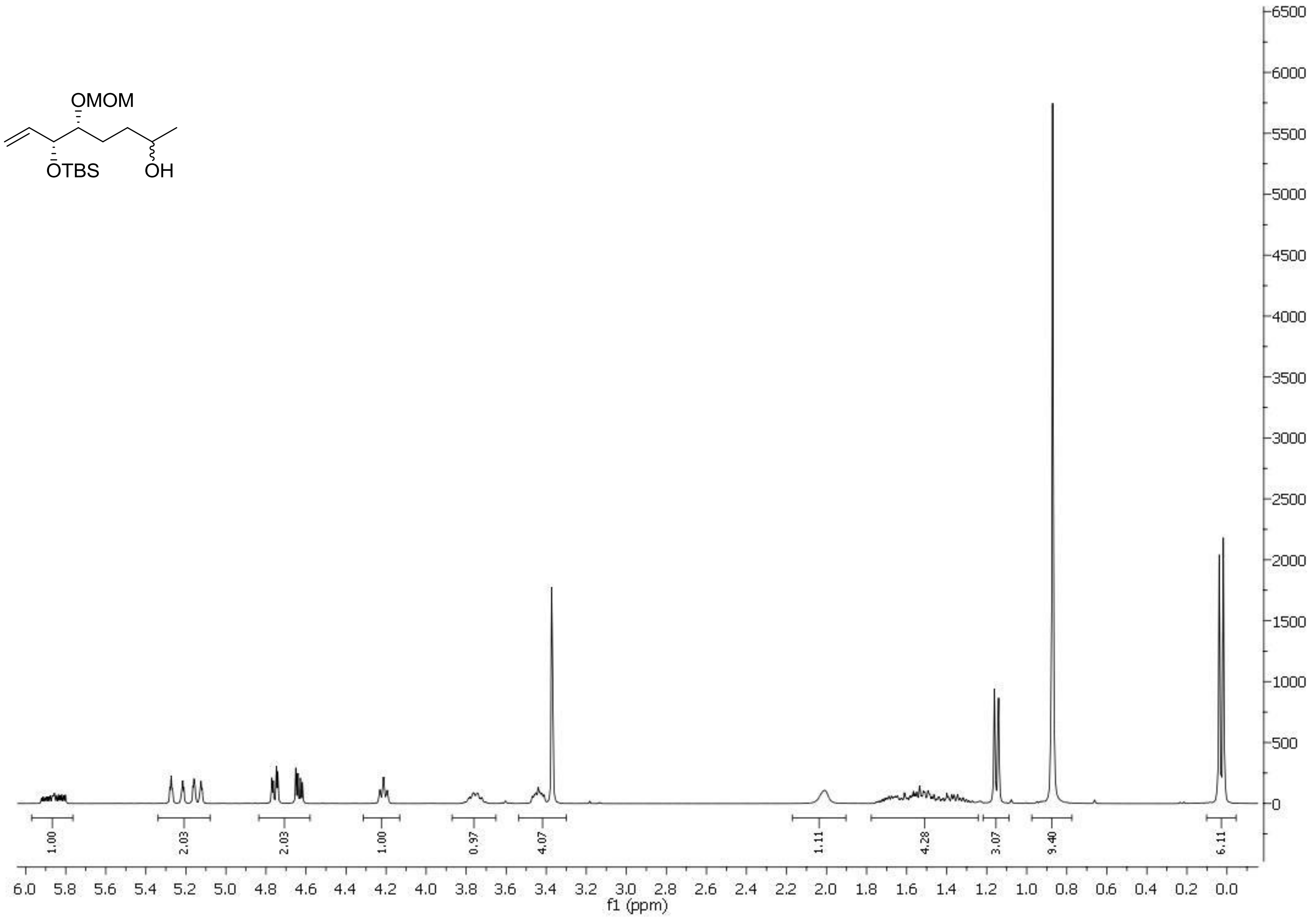

^{13}C NMR-APT (75 MHz, $CDCl_3$) of **21**

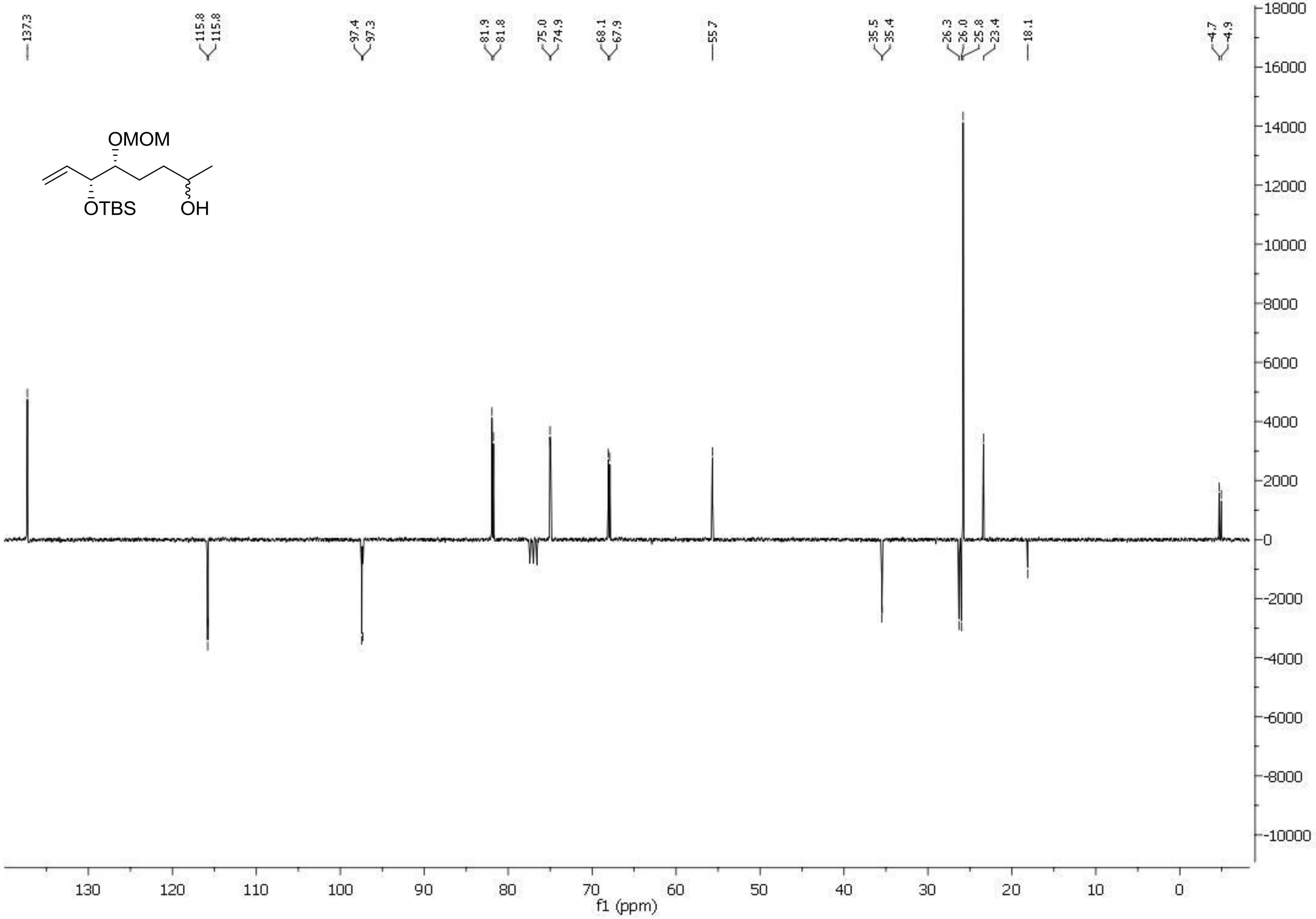

*B11 (4R,5R,E)-Methyl 5-(tert-butyldimethylsilyloxy)-4-hydroxyhepta-2,6-dienoate (**23**)*

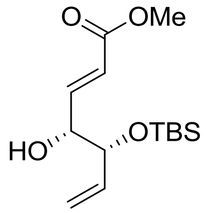

To a solution of **10** (1.10 g, 4.8 mmol) in CH_2Cl_2 (4.8 mL) were added methyl acrylate (4.3 mL, 48 mmol) and Ru catalyst **A** (31 mg, 1 mol %) at room temperature. The solution was stirred for 1 h at room temperature, and the solvent was evaporated. The residue was purified by column chromatography on silica (eluent: hexanes/MTBE 5:1) to give **23** (1.23 g, 90%) as a colourless oil: $[\alpha]^{24}_D$ = +13.6 (*c* 0.61, CH_2Cl_2); ^{1}H NMR (300 MHz, $CDCl_3$) δ 6.92 (dd, *J* = 15.7, 4.2, 1H), 6.12 (dd, *J* = 15.7, 1.9, 1H), 5.82 (ddd, *J* = 17.1, 10.3, 6.7, 1H), 5.27 (dd, *J* = 17.0, 1.3, 1H), 5.23 (dd, *J* = 10.4, 1.4, 1H), 4.13 (ddd, *J* = 5.9, 4.2, 1.8, 1H), 4.02 (ddm, *J* = 6.6, 5.7, 1H), 3.73 (s, 3H), 2.56 (bs, 1H), 0.89 (s, 9H), 0.06 (s, 3H), 0.04 (s, 3H); ^{13}C NMR (75 MHz, $CDCl_3$) δ 166.7 (0), 146.6 (1), 137.1 (1), 121.5 (1), 117.9 (2), 76.9 (1), 74.0 (1), 51.5 (3), 25.7 (3), 18.1 (0), −4.3 (3), −5.0 (3); IR (neat) *ν* 3480 (w), 2954 (w), 2857 (w), 1724 (s), 1658 (w), 1437 (m), 1255 (s); MS (ESI) *m/z* 122 (40), 269 (30), 309 ($[M+Na]^+$, 100); HRMS (ESI) calcd for $C_{14}H_{26}O_4NaSi^+$ ($[M+Na]^+$) 309.1498, found 309.1473; Anal. Calcd for $C_{14}H_{26}O_4Si$: C, 58.7; H, 9.2; Found: C, 58.5; H, 9.4.

^{1}H NMR (300 MHz, $CDCl_3$) of **23**

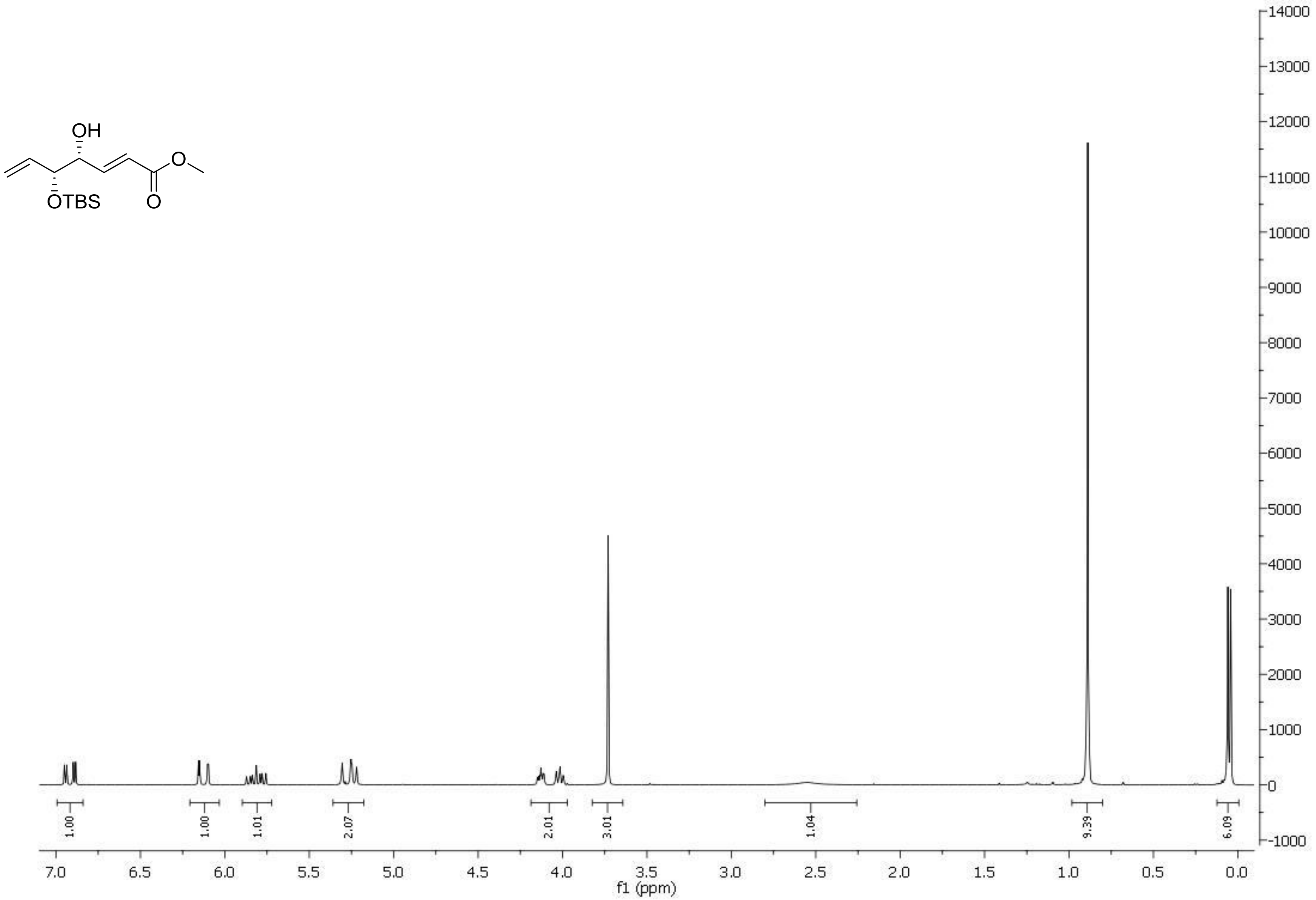

^{13}C NMR-APT (75 MHz, $CDCl_3$) of **23**

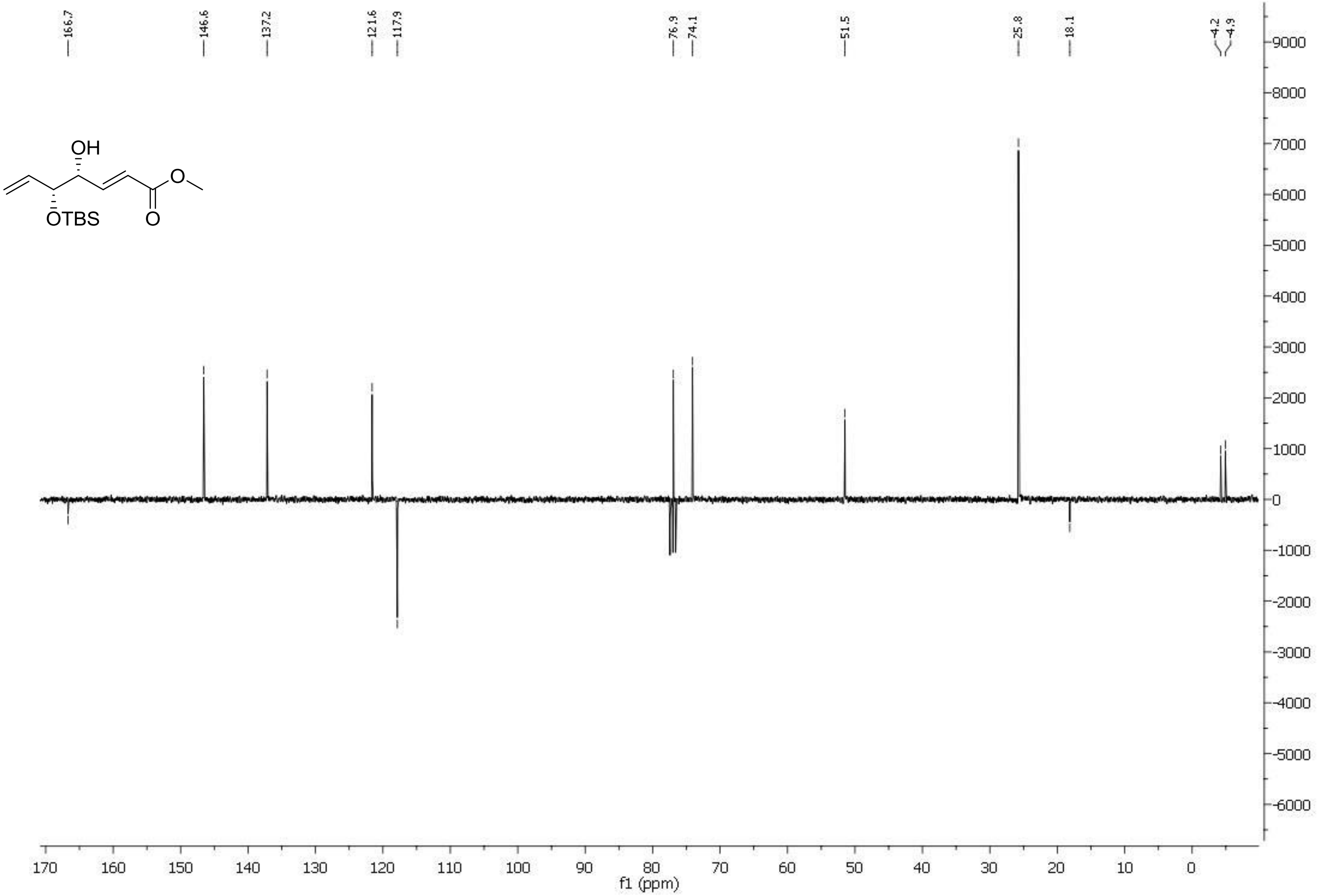

*B12 (4R,5R,E)-Methyl 5-(tert-butyldimethylsilyloxy)-4-(methoxymethoxy)hepta-2,6-dienoate (**24**)*

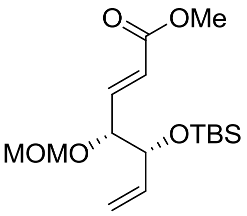

To a solution of **23** (2.0 g, 7.0 mmol) in CH_2Cl_2 (50 mL) were added DIPEA (2.4 mL, 14.0 mmol) and MOM-Br (0.96 mL, 10.5 mmol) at 0 °C. The solution was stirred for 12 h at 40 °C. After this time, water (50 mL) was added and the aqueous layer was extracted three times with MTBE. The organic layers were dried with $MgSO_4$, filtered, and evaporated. The residue was purified by column chromatography on silica (eluent: hexanes/MTBE 10:1) to give **24** (2.2 g, 93%) as a colourless oil: $[\alpha]^{23}_D$ = +20.3 (*c* 0.42, CH_2Cl_2); ^{1}H NMR (300 MHz, $CDCl_3$) δ 6.88 (dd, *J* = 15.8, 5.2, 1H), 6.04 (dd, *J* = 15.8, 1.5, 1H), 5.81 (ddd, *J* = 17.2, 10.5, 5.6, 1H), 5.26 (dd, *J* = 17.2, 1.5, 1H), 5.17 (dd, *J* = 10.6, 1.6, 1H), 4.68 (d, *J* = 6.8, 1H), 4.64 (d, *J* = 6.7, 1H), 4.24 (ddd, *J* = 5.6, 5.6, 1.2, 1H), 4.17 (ddd, *J* = 5.5, 5.3, 1.5 1H), 3.73 (s, 3H), 3.37 (s, 3H), 0.90 (s, 9H), 0.07 (s, 3H), 0.05 (s, 3H); ^{13}C NMR (75 MHz, $CDCl_3$) δ 166.4 (0), 144.9 (1), 136.6 (1), 122.4 (1), 116.6 (2), 95.7 (2), 78.9 (1), 75.1 (1), 55.6 (3), 51.4 (3), 25.7 (3), 18.1 (0), −4.8 (3), −5.0 (3); IR (neat) *ν* 2953 (w), 2932 (w), 2857 (w), 1727 (s), 1661 (w), 1467 (w), 1255 (s); MS (ESI) *m/z* 269 (40), 353 ($[M+Na]^+$, 100); HRMS (ESI) calcd for $C_{16}H_{30}O_5NaSi^+$ ($[M+Na]^+$) 353.1760, found 353.1735; Anal. Calcd for $C_{16}H_{30}O_5Si$: C, 58.2; H, 9.2; Found: C, 58.0; H, 9.3.

^{1}H NMR (300 MHz, $CDCl_3$) of **24**

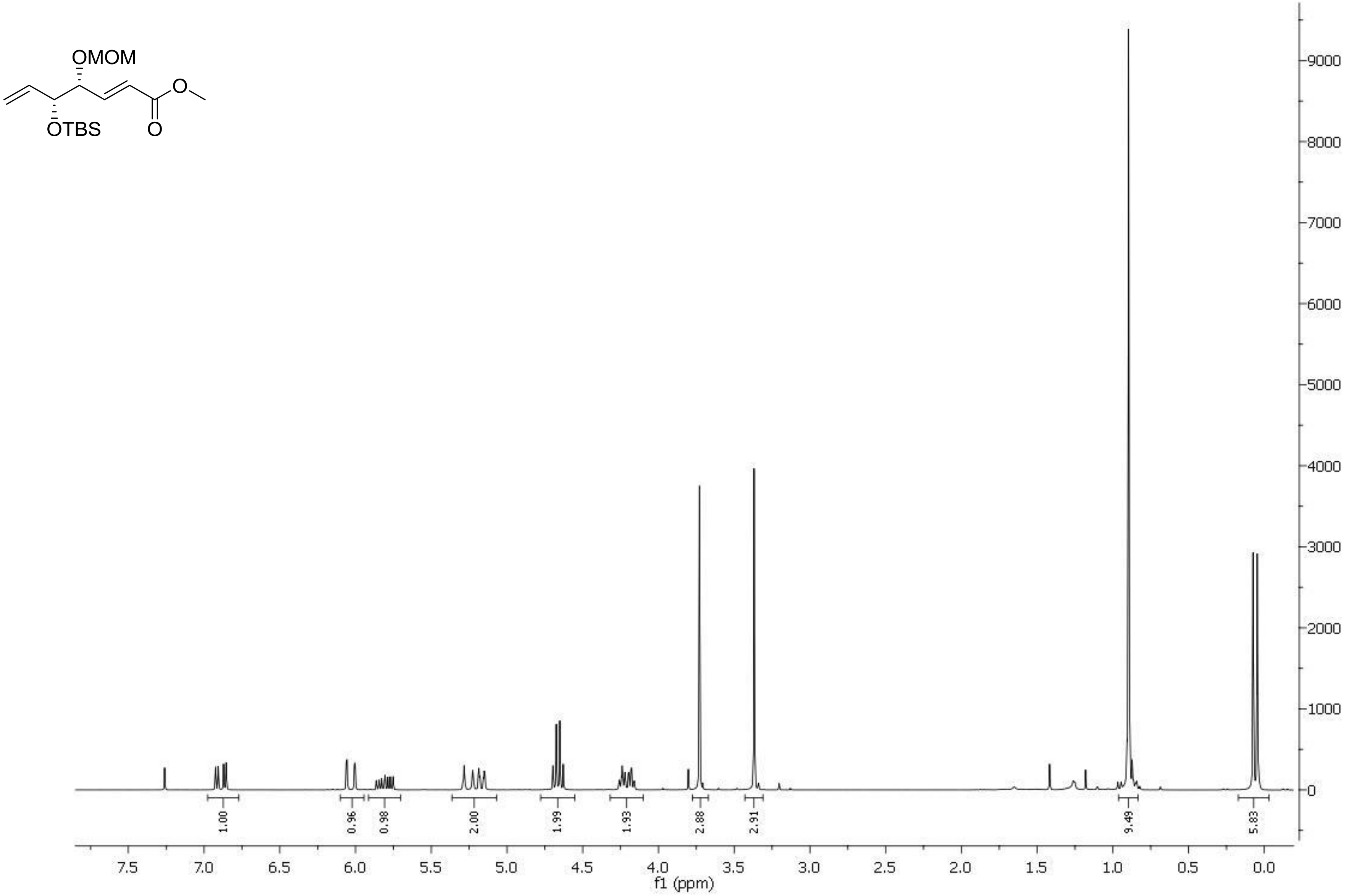

^{13}C NMR-APT (75 MHz, $CDCl_3$) of **24**

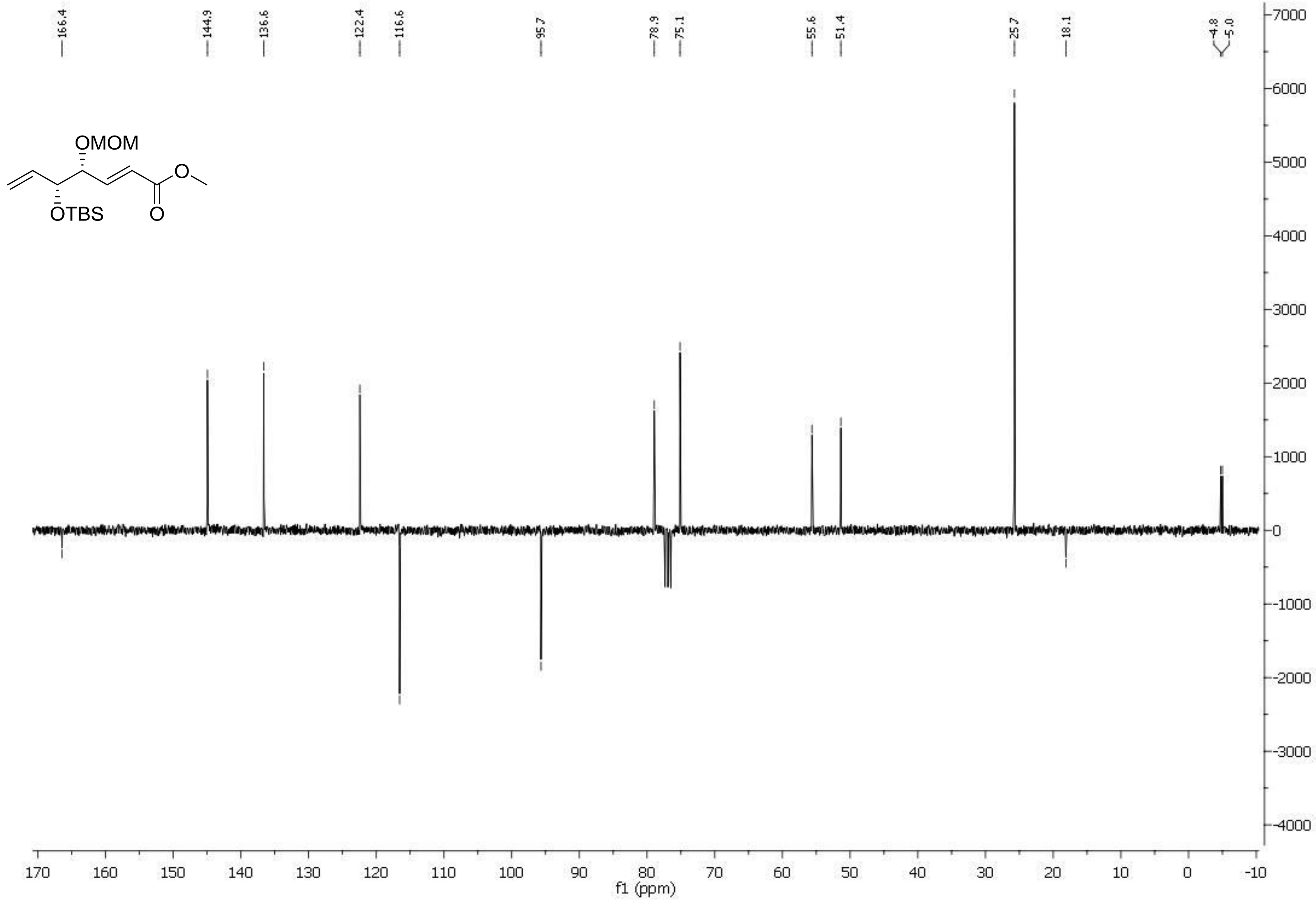

*B13 (4R,5R)-Methyl 5-(tert-butyldimethylsilyloxy)-4-(methoxymethoxy)hept-6-enoate (**25**)*

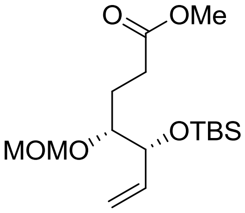

$Cu(OAc)_2 \cdot H_2O$ (30 mg, 2.5 mol %) and BDP (13.5 mg, 0.5 mol %) were dissolved in dry, degassed toluene (10 mL) and *tert*-butanol (5 mL). The mixture was stirred for 10 min at ambient temperature, and PMHS (0.84 mL, 12.0 mmol) was added. Stirring was continued for 0.5 h, after which time the colour changed from blue to green. A solution of **24** (2.00 g, 6.0 mmol) in toluene (2.0 mL) was added and the solution was stirred at ambient temperature for 12 h. The reaction mixture was diluted with MTBE, and washed with a saturated aqueous $NaHCO_3$ solution, followed by aqueous HCl (1 M). The aqueous layer was extracted twice with MTBE. The organic layers were dried with $MgSO_4$, filtered, and evaporated. The residue was purified by column chromatography on silica (eluent: hexanes/MTBE 10:1) to give **25** (1.93 g, 97%) as a colourless oil: $[\alpha]^{23}_{D}$ = +55.6 (*c* 0.75, CH_2Cl_2); ^1H NMR (300 MHz, $CDCl_3$) δ 5.87 (ddd, *J* = 17.2, 10.5, 5.3, 1H), 5.26 (dd, *J* = 17.1, 1.7, 1H), 5.16 (dd, *J* = 10.6, 1.7, 1H), 4.72 (d, *J* = 6.9, 1H), 4.62 (d, *J* = 6.9, 1H), 4.23 (ddd, *J* = 5.4, 5.4, 1.4, 1H), 3.64 (s, 3H), 3.46 (ddd, *J* = 9.0, 5.4, 3.6, 1H), 3.37 (s, 3H), 2.50-2.35 (2H), 1.92 (m, 1H), 1.61 (m, 1H), 0.88 (s, 9H), 0.05 (s, 3H), 0.03 (s, 3H); ^{13}C NMR (75 MHz, $CDCl_3$) δ 173.9 (0), 137.0 (1), 116.0 (2), 97.4 (2), 80.7 (1), 74.9 (1), 55.7 (3), 51.3 (3), 30.4 (2), 25.8 (3), 25.3 (2), 18.1 (0), −4.7 (3), −5.0 (3); IR (neat) *v* 2952 (w), 2933 (w), 2856 (w), 1740 (s), 1439 (w), 1253 (s); MS (ESI) *m/z* 333 ($[M+H]^+$, 100); HRMS (ESI) calcd for $C_{16}H_{33}O_5Si^+$ ($[M+H]^+$) 333.2097, found 333.2084; Anal. Calcd for $C_{16}H_{32}O_5Si$: C, 57.8; H, 9.7; Found: C, 57.8; H, 9.9.

^{1}H NMR (300 MHz, $CDCl_3$) of **25**

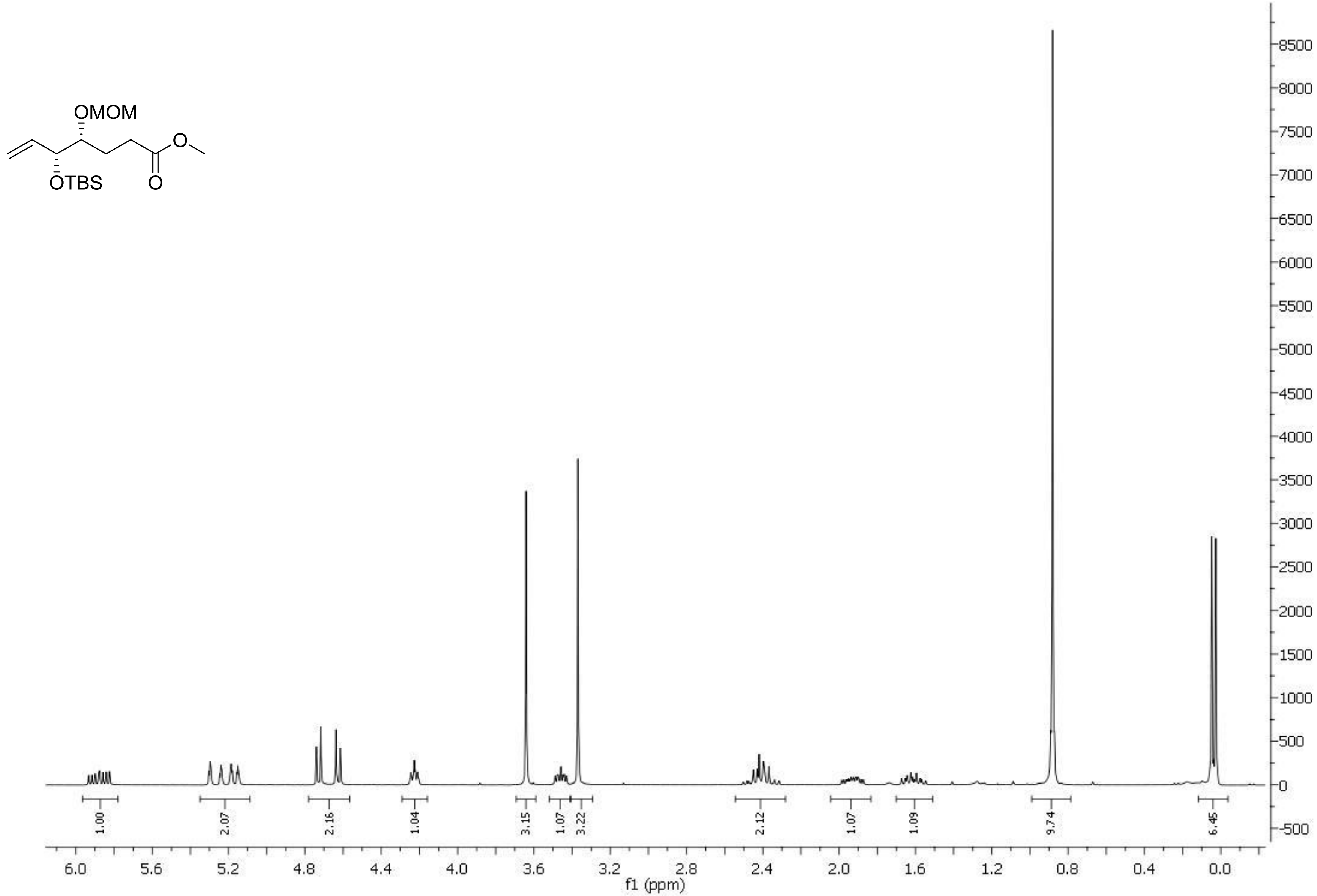

^{13}C NMR-APT (75 MHz, $CDCl_3$) of **25**

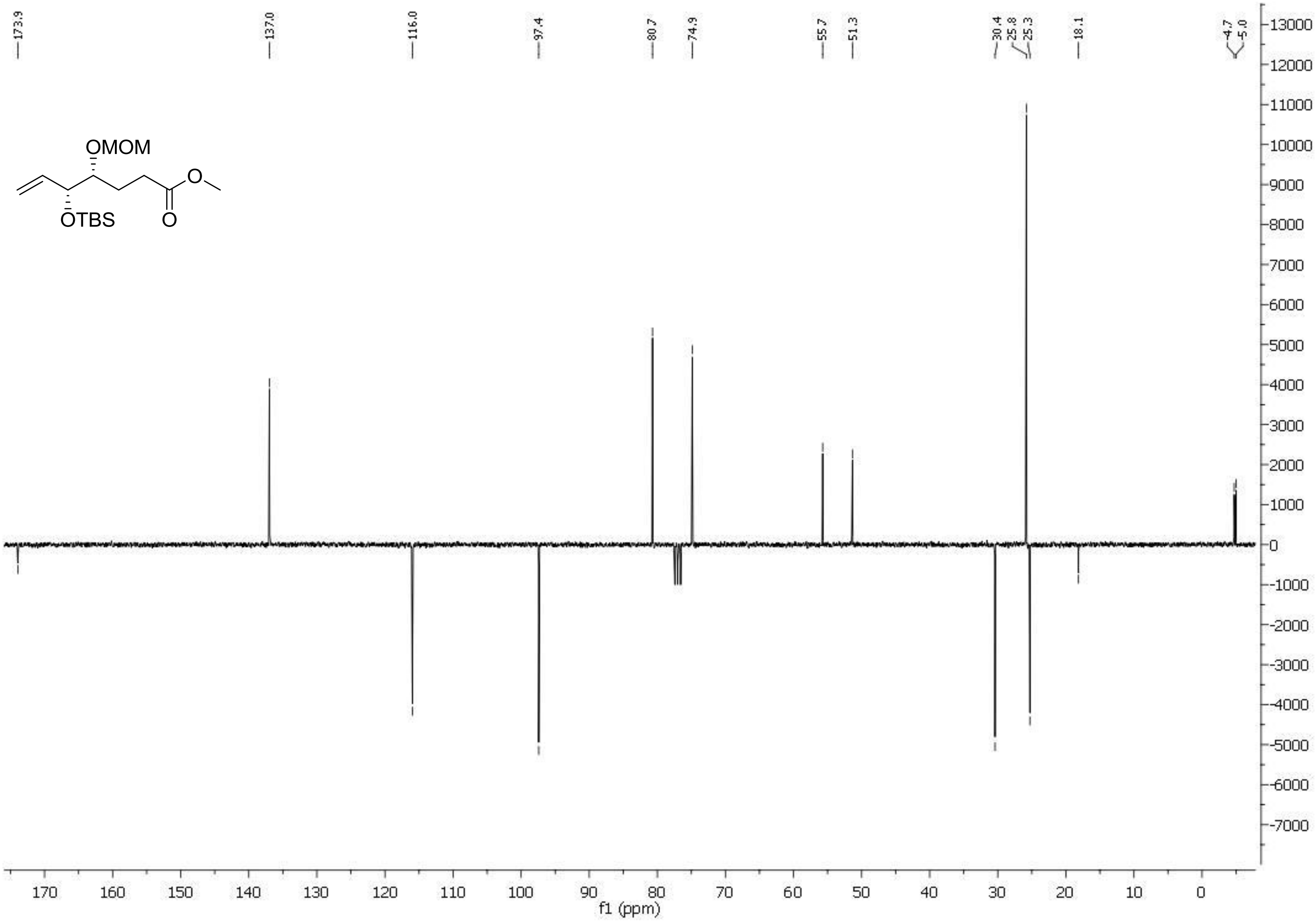

*B14 (2R,5R,6R)-6-(tert-Butyldimethylsilyloxy)-5-(methoxymethoxy)oct-7-en-2-yl acetate (**26**)*

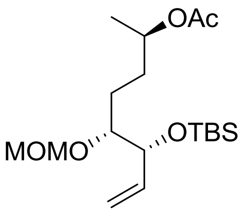

*From **21** (Ru–lipase-catalyzed DKR, Table 4, entry 6):* To a solution of potassium *tert*-butoxide (2.8 mg, 3 mol %) in toluene (3 mL) was added catalyst **D** (12.5 mg, 2 mol %) and the reaction mixture was stirred for 10 min at ambient temperature. After this time, novozyme 435 (10 mg), sodium carbonate (106 mg, 1.0 mmol), alcohol **21** (318 mg, 1.0 mmol) and isopropenyl acetate (0.33 mL, 3 mmol) was added and the reaction mixture was stirred for 7 d at 30 °C. After this time, the mixture was filtered through a pad of celite, washed three times with MTBE and the solvent was evaporated. The residue was purified by column chromatography on silica (eluent: hexanes/MTBE 5:1) to give **26** (242 mg, 67%) and (2*S*)-**21** (100 mg, 31%) as colourless oils.

*From **21** (lipase-catalyzed kinetic resolution, Table 4, entry 1):* To a solution of **21** (3.85 g, 12.1 mmol) in toluene (20 mL) was added novozyme 435 (120 mg), isopropenyl acetate (1.23 mL, 12.1 mmol) and the reaction mixture was stirred for 24 h at ambient temperature. After this time, the mixture was filtered through a pad of celite, washed three times with MTBE and the solvent was evaporated. The residue was purified by column chromatography on silica (eluent: hexanes/MTBE 5:1) to give **26** (2.16 g, 49%) and (2*S*)-**21** (1.71 g, 44%) as colourless oils.

From (2*S*)-**21***:* To a solution of diisopropyl azodicarboxylate (0.42 mL, 2 mmol) in THF (6 mL) was added triphenylphosphine (524 mg, 2 mmol) at ambient temperature and the mixture was stirred for 30 min. A solution of (2*S*)-**21** (318 mg, 1 mmol) in THF (4 mL) and acetic acid (0.12 mL, 2 mmol) in THF (4 mL) were added successively and the reaction mixture was stirred for 20 min. at ambient temperature. The reaction was quenched by addition of saturated NH_4Cl (aq.) solution and the organic layer was separated. The aqueous layer was extracted three times with methyl *tert*-butyl ether and the combined organic layers were dried with $MgSO_4$, filtered and

evaporated. The residue was purified by column chromatography on silica (eluent: hexanes/MTBE 10:1) to give **26** (297 mg, 82%) as a colourless oil. *Analytical data for (2R,5R,6R)-6-(tert-butyldimethylsilyloxy)-5-(methoxymethoxy)oct-7-en-2-yl acetate (**26**):* $[\alpha]^{23}_{D}$ = +50.7 (*c* 0.51, CH_2Cl_2); ^1H NMR (300 MHz, $CDCl_3$) δ 5.87 (ddd, *J* = 17.2, 10.5, 5.5, 1H), 5.26 (dd, *J* = 17.2, 1.7, 1H), 5.16 (dd, *J* = 10.5, 1.5, 1H), 4.86 (m, 1H), 4.75 (d, *J* = 6.9, 1H), 4.64 (d, *J* = 6.9, 1H), 4.23 (ddd, *J* = 5.5, 5.4, 1.5, 1H), 3.43 (m, 1H), 3.38 (s, 3H), 2.00 (s, 3H), 1.70-1.55 (3H), 1.30 (m, 1H), 1.18 (d, *J* = 6.3, 3H), 0.89 (s, 9H), 0.05 (s, 3H), 0.03 (s, 3H); ^{13}C NMR (75 MHz, $CDCl_3$) δ 170.7 (0), 137.1 (1), 115.9 (2), 97.3 (2), 81.6 (1), 74.7 (1), 71.1 (1), 55.7 (3), 32.2 (2), 25.8 (3), 25.7(2), 21.3 (3), 19.9 (3), 18.1 (0), −4.7 (3), −5.0 (3); IR (neat) $\tilde{\nu}$ 2932 (w), 2889 (w), 2858 (w), 1737 (s), 1467 (w), 1243 (s), 1034 (s); HRMS (ESI) calcd. for $C_{18}H_{36}O_5NaSi^+$ ($[M+Na]^+$) 383.2230, found 383.2209; Anal. Calcd. for $C_{18}H_{36}O_4Si$: C, 60.0; H, 10.1; found: C, 59.6; H, 10.2. *Analytical data for (2S,5R,6R)-6-(tert-butyldimethylsilyloxy)-5-(methoxymethoxy)oct-7-en-2-ol (2S)-**21**):* $[\alpha]^{23}_{D}$ = +62.6 (*c* 0.32, CH_2Cl_2); ^1H NMR (300 MHz, $CDCl_3$) δ 5.87 (ddd, *J* = 17.2, 10.6, 5.5, 1H), 5.26 (dd, *J* = 17.2, 1.7, 1H), 5.16 (dd, *J* = 10.5, 1.7, 1H), 4.77 (d, *J* = 6.9, 1H), 4.65 (d, *J* = 6.9, 1H), 4.24 (ddd, *J* = 5.5, 5.5, 1.6, 1H), 3.79 (m, 1H), 3.47 (ddd, *J* = 8.4, 5.5, 3.1, 1H), 3.39 (s, 3H), 2.01 (bs, 1H), 1.70-1.35 (4H), 1.15 (d, *J* = 6.2, 3H), 0.89 (s, 9H), 0.06 (s, 3H), 0.04 (s, 3H); ^{13}C NMR (75 MHz, $CDCl_3$) δ 137.3 (1), 115.8 (2), 97.4 (2), 81.8 (1), 75.0 (1), 68.0 (1), 55.7 (3), 35.5 (2), 26.0 (2), 25.8 (3), 23.4 (3), 18.2 (0), −4.6 (3), −4.9 (3).

^{1}H NMR (300 MHz, $CDCl_3$) of (2*S*)-**21**

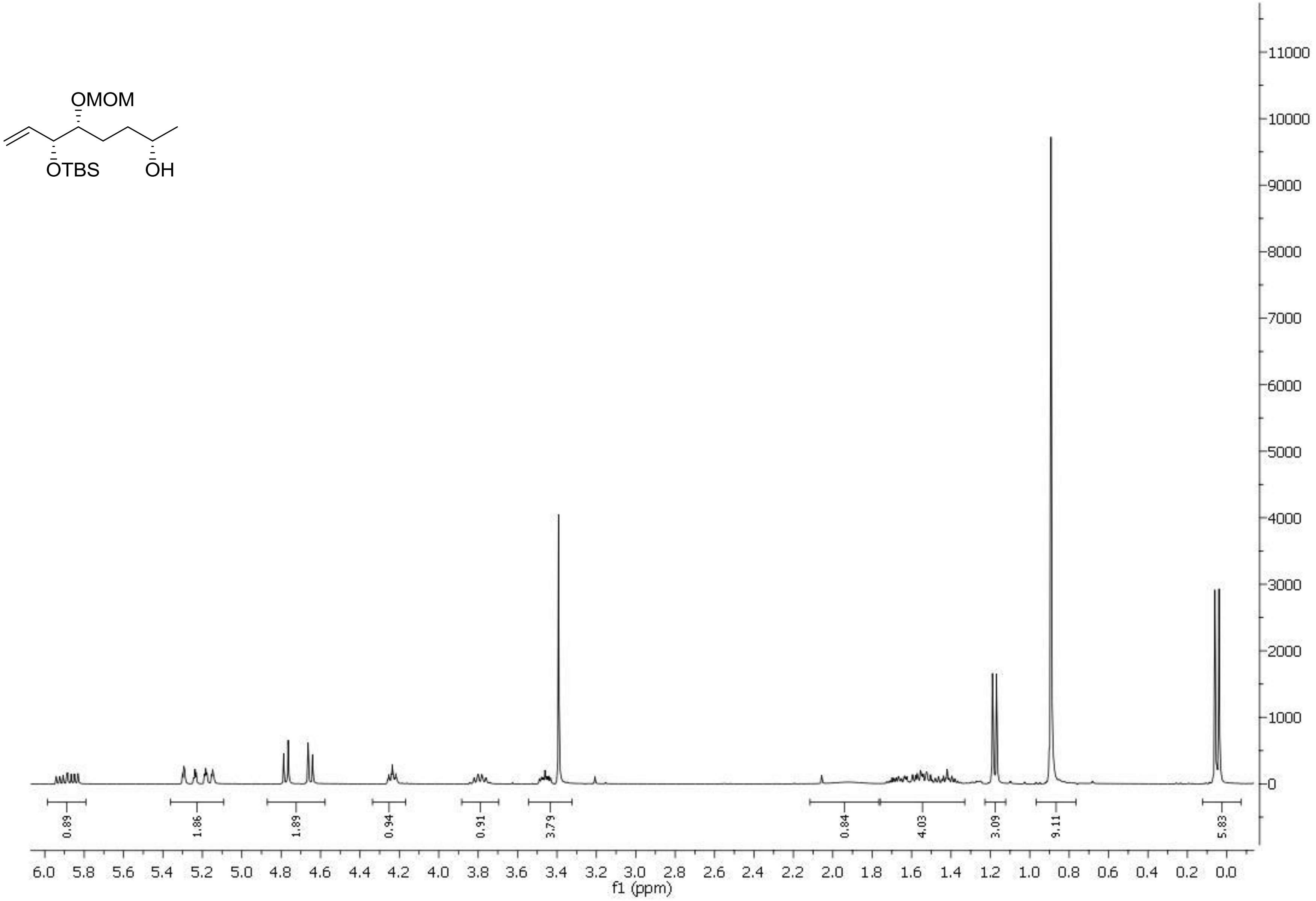

^{13}C NMR-APT (75 MHz, $CDCl_3$) of (2*S*)-**21**

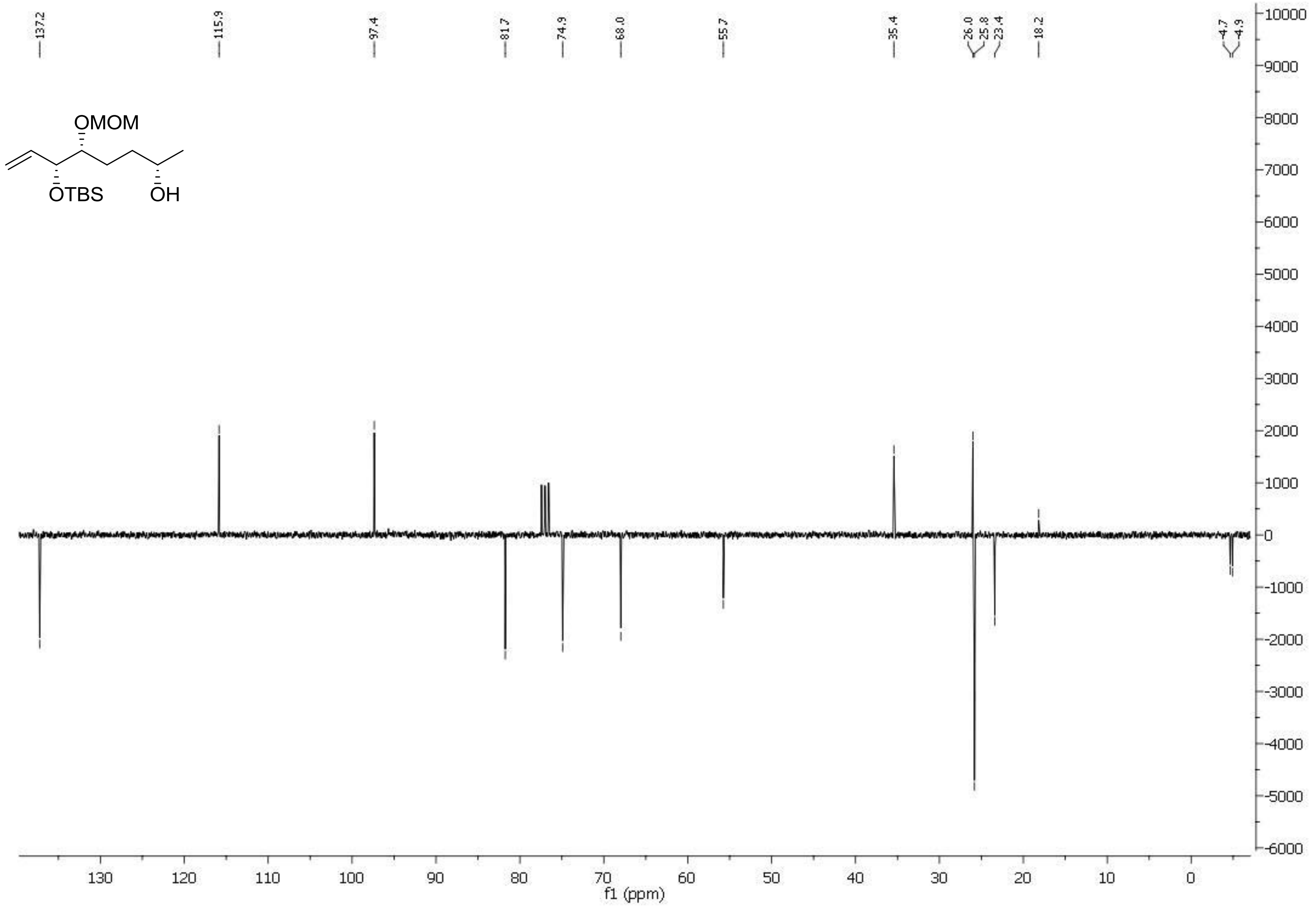

^{1}H NMR (300 MHz, $CDCl_3$) of **26**

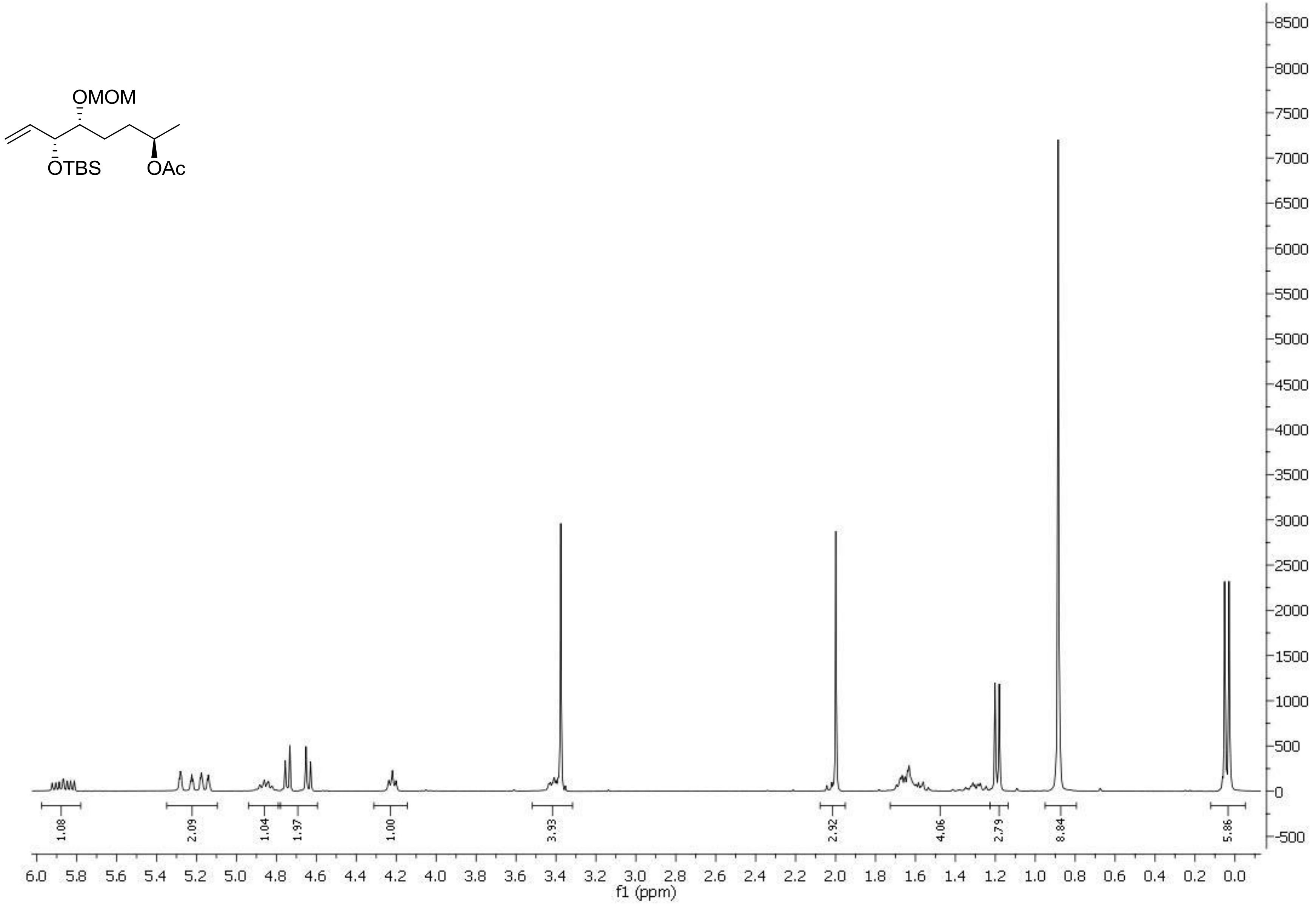

^{13}C NMR-APT (75 MHz, $CDCl_3$) of **26**

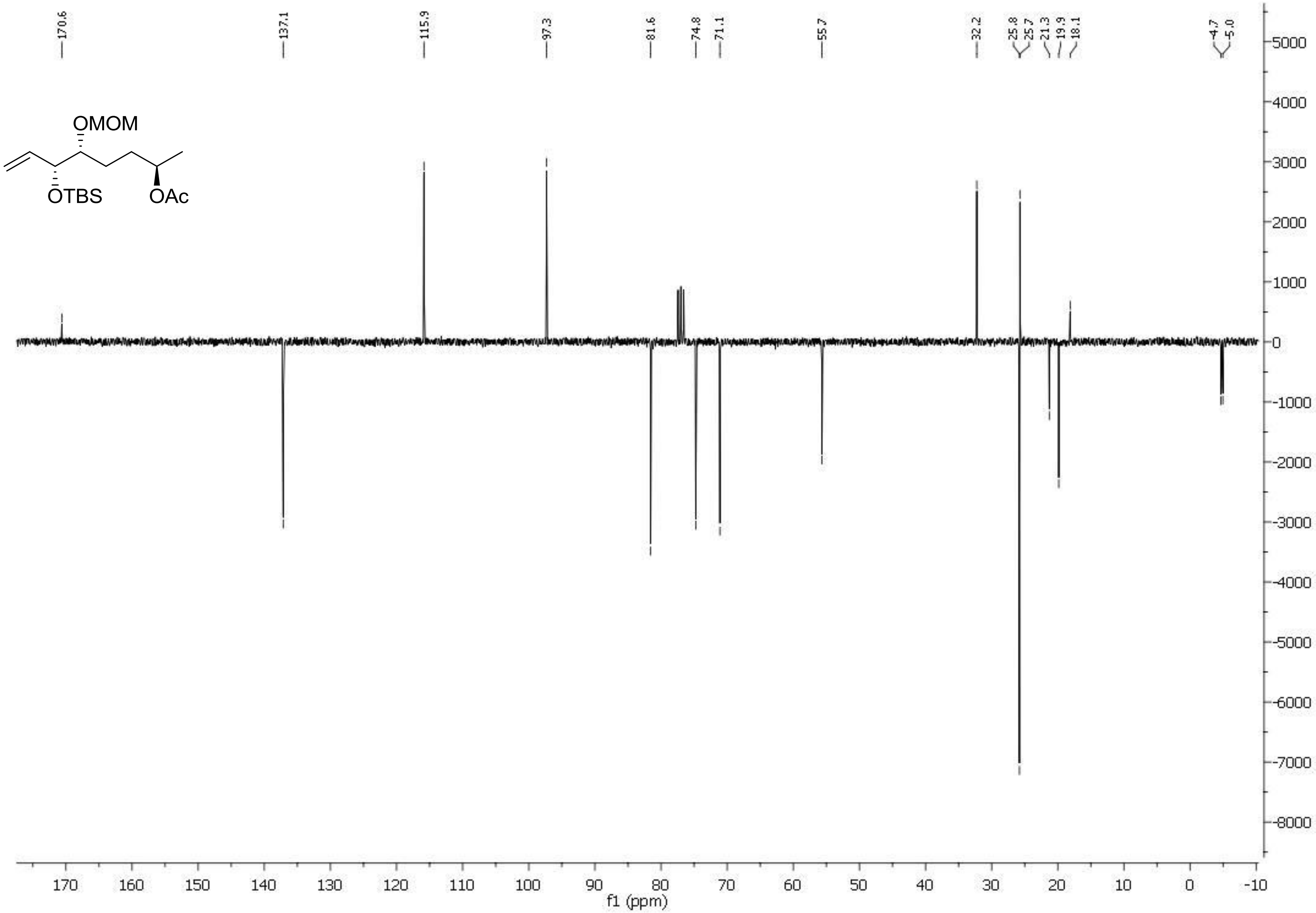

*B15 (2R,5R,6R)-6-Hydroxy-5-(methoxymethoxy)oct-7-en-2-yl acetate (**27**)*

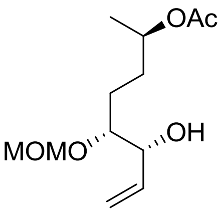

To a solution of **26** (432 mg, 1.20 mmol) in THF (10 mL) was added tetrabutylammonium fluoride (435 mg, 1.44 mmol) and the mixture was stirred for 16 h at room temperature. The reaction was quenched by the addition of water and the aqueous layer was extracted three times with MTBE. The combined organic layers were dried with $MgSO_4$, filtered and evaporated. The residue was purified by column chromatography on silica (eluent: hexanes/MTBE 1:1) to give **27** (272 mg, 92%) as a colourless oil: $[\alpha]^{22}_D$ = +52.4 (*c* 0.21, MeOH); ^{1}H NMR (300 MHz, $CDCl_3$) δ 5.78 (ddd, *J* = 17.0, 10.4, 6.3, 1H), 5.32 (d, *J* = 17.2, 1H), 5.17 (d, *J* = 10.4, 1H), 4.82 (m, 1H), 4.69 (d, *J* = 6.8, 1H), 4.63 (d, *J* = 6.8, 1H), 3.97 (dd, *J* = 6.3, 6.2, 1H), 3.37 (s, 3H), 3.36 (m, 1H), 3.15 (bs, 1H), 1.97 (s, 3H), 1.70-1.35 (4H), 1.16 (d, *J* = 6.3, 3H); ^{13}C NMR (75 MHz, $CDCl_3$) δ 170.5 (0), 137.2 (1), 116.8 (2), 97.3 (2), 82.9 (1), 74.5 (1), 70.8 (1), 55.8 (3), 31.4 (2), 27.0 (2), 21.2 (3), 19.7 (3); IR (neat) *v* 3452 (w), 2936 (w), 1731 (s), 1446 (w), 1373 (m), 1243 (s), 1031 (s); MS (ESI) *m/z* 122 (75), 197 (70), 247 ($[M+H]^+$, 5); HRMS (ESI) calcd. for $C_{12}H_{23}O_5^+$ ($[M+H]^+$) 247.1545, found 247.1549; Anal. calcd. for $C_{12}H_{22}O_5$: C, 58.5; H, 9.0; found: C, 58.5; H, 8.8.

^{1}H NMR (300 MHz, $CDCl_3$) of **27**

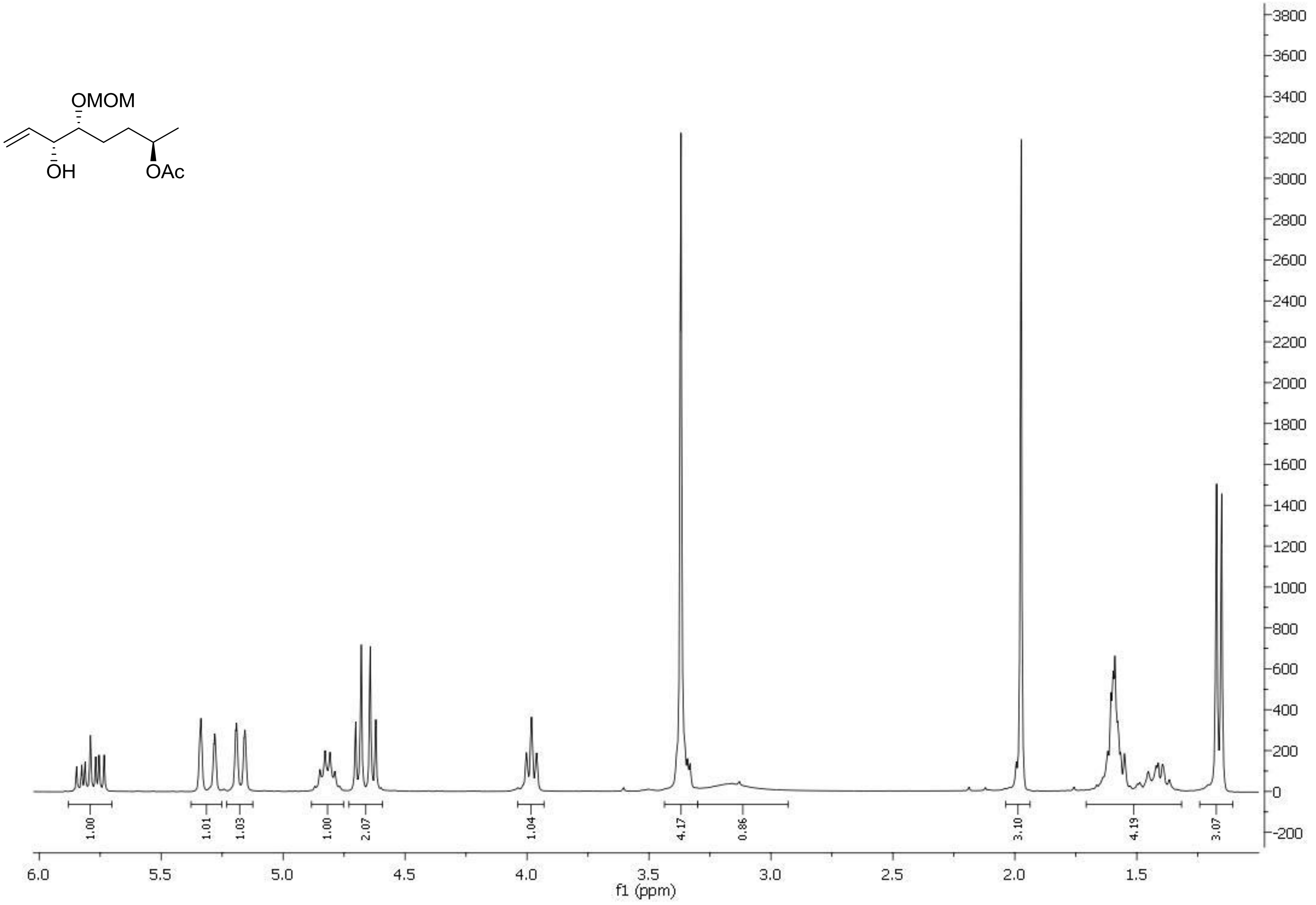

^{13}C NMR-APT (75 MHz, $CDCl_3$) of **27**

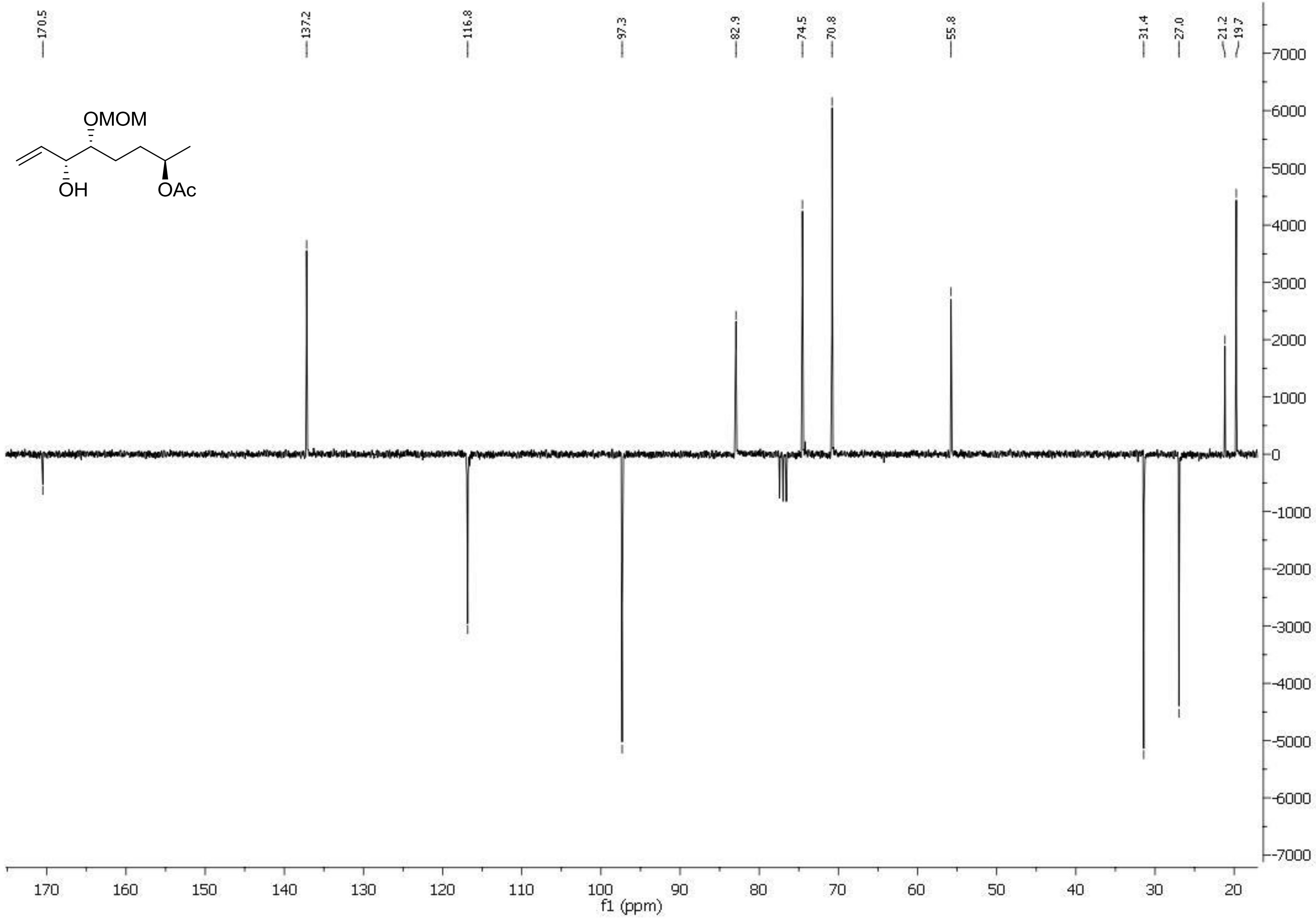

*B16 (3R,4R,7R)-7-Acetoxy-4-(methoxymethoxy)oct-1-en-3-yl but-3-enoate (**28**)*

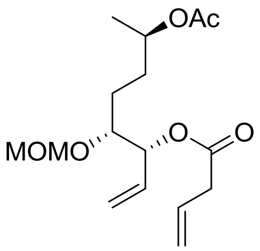

To a solution of allylic alcohol **27** (250 mg, 1.02 mmol) in dichloromethane (10 mL) was added vinylacetic acid (0.17 mL, 2.04 mmol), dicyclohexylcarbodiimide (420 mg, 2.04 mmol) and 4-*N*,*N*-dimethylaminopyridine (24 mg, 20 mol %) at 0 °C. The mixture was allowed to warm to room temperature and stirred for 2 h. The solution was filtered and washed three times with dichloromethane. The combined organic layers were washed with 1 M HCl (aq) solution and with $NaHCO_3$ (aq) solution, dried with $MgSO_4$, filtered and evaporated. The residue was purified by column chromatography on silica (eluent: hexanes/MTBE 5:1) to give **28** (302 mg, 94%) as a colourless oil: $[\alpha]^{23}_{D}$ = +33.7 (*c* 0.33, CH_2Cl_2); ^{1}H NMR (300 MHz, $CDCl_3$) δ 5.89 (ddt, *J* = 17.3, 10.0, 7.0, 1H), 5.79 (ddd, *J* = 17.1, 10.6, 6.3, 1H), 5.37-5.10 (5H), 4.84 (m, 1H), 4.69 (d, *J* = 6.9, 1H), 4.63 (d, *J* = 6.9, 1H), 3.60 (m, 1H), 3.36 (s, 3H), 3.11 (dd, *J* = 7.0, 1.3, 1H), 1.99 (s, 3H), 1.65-1.40 (4H), 1.17 (d, *J* = 6.3, 3H); ^{13}C NMR (75 MHz, $CDCl_3$) δ 170.5 (0), 170.2 (0), 132.7 (1), 130.0 (1), 118.6 (2), 118.4 (2), 96.8 (2), 78.5 (1), 75.5 (1), 70.8 (1), 55.8 (3), 39.2 (2), 31.4 (2), 26.5 (2), 21.2 (3), 19.8 (3); IR (neat) *v* 2934 (w), 1734 (s), 1646 (w), 1447 (w), 1372 (m), 1243 (s), 1034 (s); MS (ESI) *m/z* 122 (45), 137 (55), 196 (60), 253 (60), 315 ($[M+H]^+$, 1); HRMS (ESI) calcd. for $C_{16}H_{27}O_6^+$ ($[M+H]^+$) 315.1808, found 315.1793; Anal. calcd. for $C_{16}H_{26}O_6$: C, 61.1; H, 8.3; found: C, 61.3; H, 8.4.

^{1}H NMR (300 MHz, $CDCl_3$) of **28**

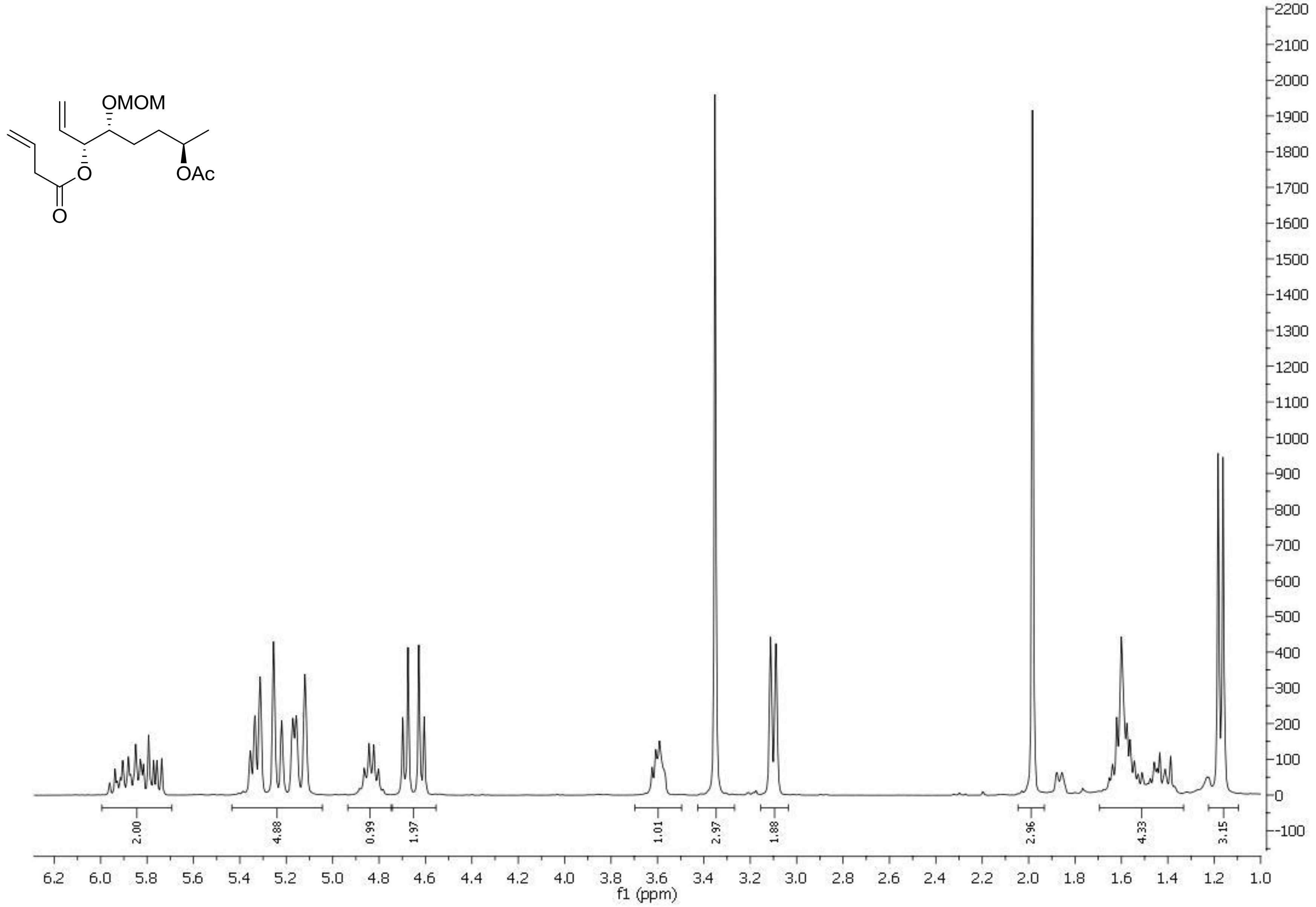

^{13}C NMR-APT (75 MHz, $CDCl_3$) of **28**

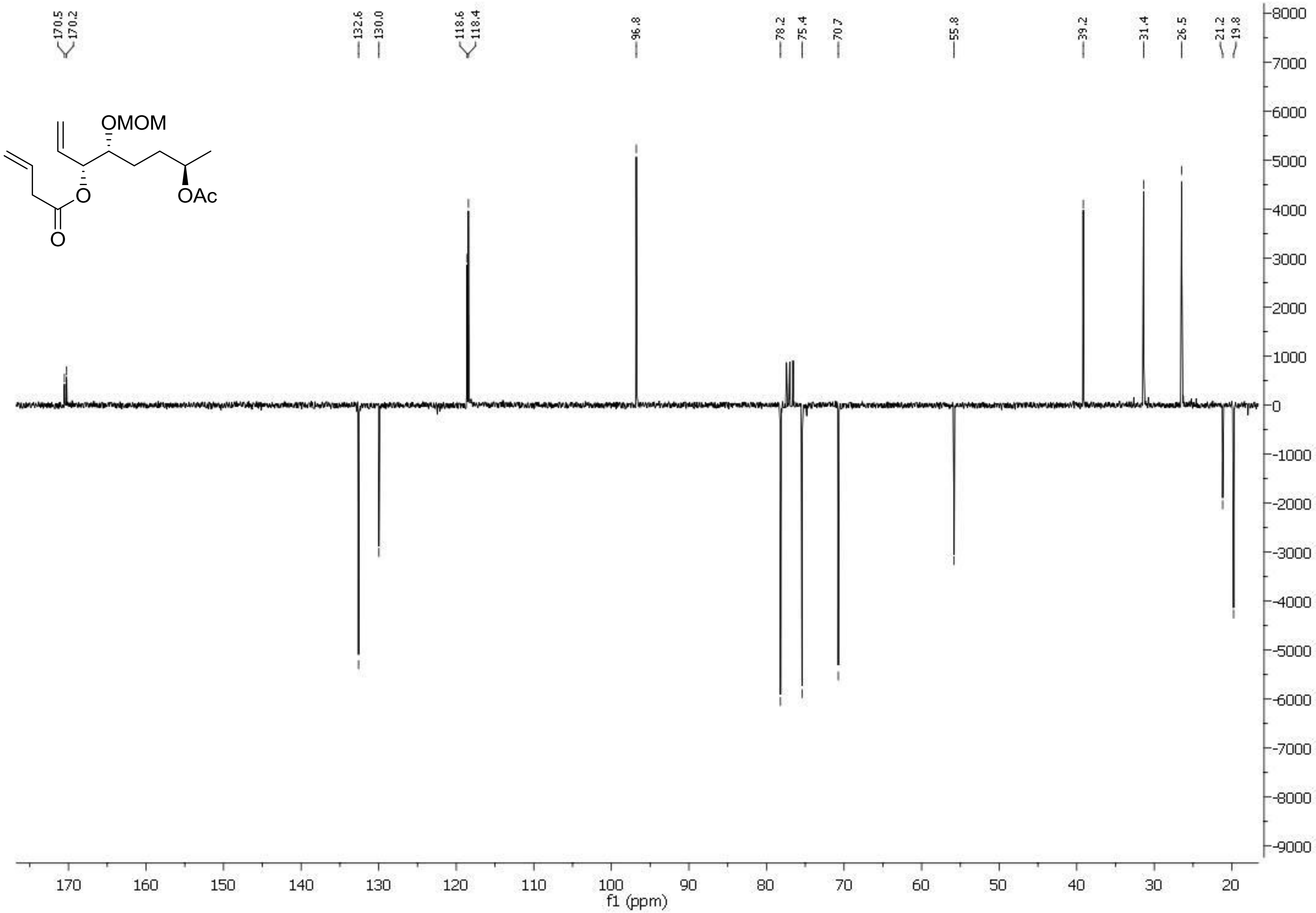

*B17 (2Z,4E,6R,9R)-9-Hydroxy-6-(methoxymethoxy)deca-2,4-dienoic acid (**29**)*[4]

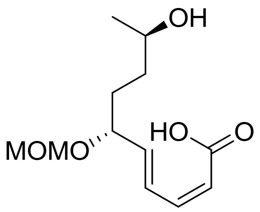

To a solution of **28** (157 mg, 0.50 mmol) in toluene (10 mL) was added Ru catalyst **B** (25 mg, 2 mol %) at 80 °C. The mixture was stirred for 30 min and sodium hydride (60 wt % in mineral oil, 30 mg, 0.75 mmol) was added. The mixture was stirred at 80 °C for 1 h and cooled to ambient temperature. Then aqueous NaOH (2 M, 10 mL) was added and the solution was stirred for 30 min at 60 °C. The reaction was quenched by the addition of water and acidified with 1 M HCl. The aqueous layer was extracted three times with methyl *tert*-butyl ether (MTBE) and the combined organic layers were dried with $MgSO_4$, filtered and evaporated. The residue was purified by column chromatography on silica (eluent: hexanes/ethyl acetate 1:1) to give **29** (99 mg, 81%) as a colourless oil: $[\alpha]^{22}_{D}$ = +10.4 (*c* 0.29, CH_2Cl_2); ^{1}H NMR (300 MHz, $CDCl_3$) δ 7.46 (dd, *J* = 15.4, 11.3, 1H), 6.64 (dd, *J* = 11.5, 11.5, 1H), 5.93 (dd, *J* = 15.5, 7.5, 1H), 5.67 (d, *J* = 11.4, 1H), 4.67 (d, *J* = 6.8, 1H), 4.58 (d, *J* = 6.8, 1H), 4.21 (m, 1H), 3.82 (m, 1H), 3.38 (s, 3H), 1.75-1.45 (4H), 1.19 (d, *J* = 6.2, 3H); ^{13}C NMR (75 MHz, $CDCl_3$) δ 170.5 (0), 145.4 (1), 143.9 (1), 128.0 (1), 117.6 (1), 94.5 (2), 76.5 (1), 68.0 (1), 55.6 (3), 34.7 (2), 31.6 (2), 23.4 (3); IR (neat) ν 2934 (w), 1734 (s), 1646 (w), 1447 (w), 1372 (m), 1243 (s), 1034 (s); HRMS (ESI) calcd. for $C_{12}H_{21}O_5^+$ ($[M+H]^+$) 245.1389, found 245.1382; Anal. calcd. for $C_{12}H_{20}O_5$: C, 59.0; H, 8.3; Found: C, 59.0; H, 8.4.

[4] Compound **29** has previously been reported in the course of Sabitha's synthesis of stagonolide E, however, no characterization data were given: Sabitha, G.; Padmaja, P.; Reddy, P. N.; Jadav, S. S.; Yadav, J. S. *Tetrahedron Lett.* **2010**, *51*, 6166-6168.

^{1}H NMR (300 MHz, $CDCl_3$) of **29**

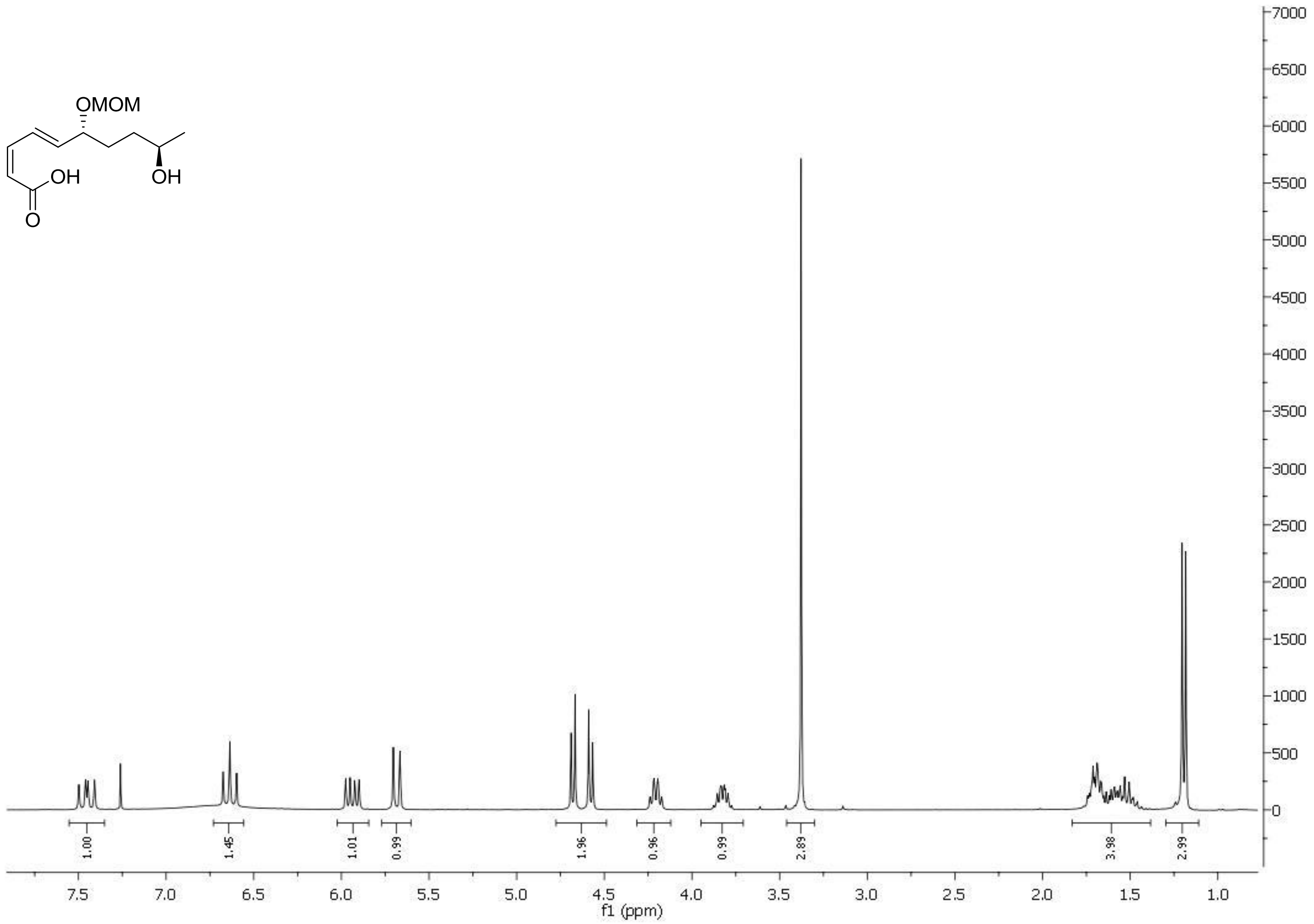

^{13}C NMR-APT (75 MHz, $CDCl_3$) of **29**

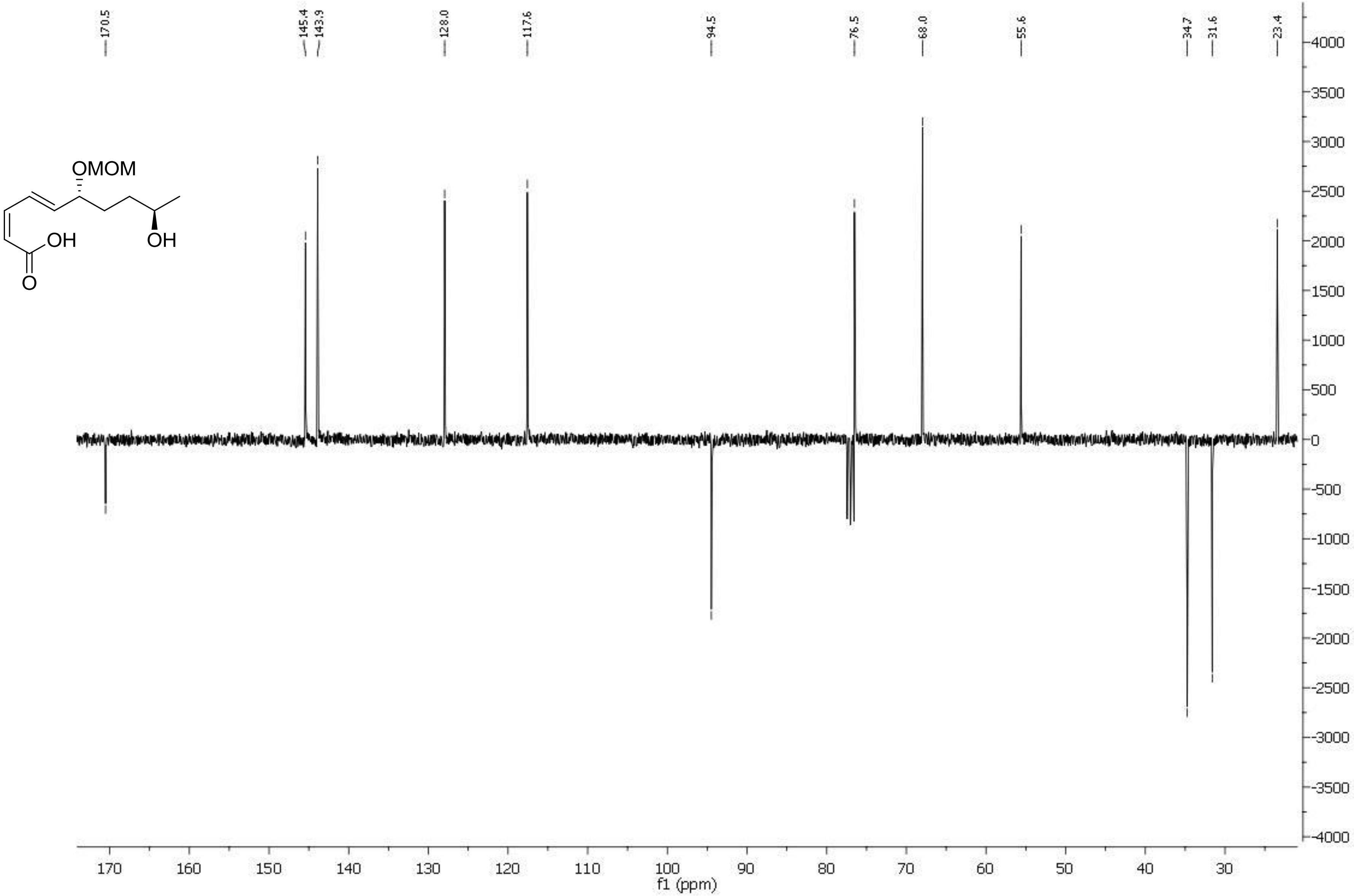

*B18 7-(tert-Butyldimethylsilyloxy)hept-1-en-3-yl but-3-enoate (**31**)*

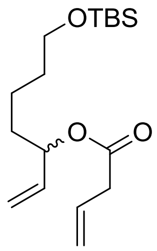

To a solution of allylic alcohol **30** (320 mg, 1.31 mmol) in dichloromethane (20 mL) was added vinylacetic acid (0.123 mL, 1.44 mmol), dicyclohexylcarbodiimide (297 mg, 1.44 mmol) and 4-*N*,*N*-dimethylaminopyridine (18 mg, 10 mol %) at 0 °C. The mixture was allowed to warm to room temperature and stirred for 2 h. The solution was filtered and washed three times with dichloromethane. The combined organic layers were washed with 1 M HCl (aq) solution and $NaHCO_3$ (aq) solution, dried with $MgSO_4$, filtered and evaporated. The residue was purified by column chromatography on silica (eluent: hexanes/MTBE 10:1) to give **31** (360 mg, 88%) as a colourless oil: ^{1}H NMR (300 MHz, $CDCl_3$) δ 5.93 (ddt, J = 17.4, 9.9, 7.0, 1H), 5.77 (ddd, J = 16.9, 10.5, 6.4, 1H), 5.27-5.12 (5H), 3.59 (t, J = 6.4, 2H), 3.09 (dt, J = 7.0, 1.4, 2H), 1.70-1.30 (6H), 0.88 (s, 9H), 0.03 (s, 6H); ^{13}C NMR (75 MHz, $CDCl_3$) δ 170.3 (0), 136.4 (1), 130.4 (1), 118.4 (2), 116.6 (2), 75.0 (1), 62.9 (2), 39.4 (2), 34.0 (2), 32.5 (2), 25.9 (3), 21.4 (2), 18.3 (0), −5.3 (3); IR (neat) ν 3084 (w), 2932 (m), 2857 (m), 1737 (s), 1097 (s); MS (ESI) 227 (60), 313 ($[M+H]^+$, 100); HRMS (ESI) calcd. for $C_{18}H_{34}O_3Si^+$ ($[M+H]^+$) 313.2199, found 313.2176; Anal. Calcd. for $C_{17}H_{32}O_3Si$: C, 65.3; H, 10.3; found: C, 65.1; H, 10.3.

^1H NMR (300 MHz, $CDCl_3$) of **31**

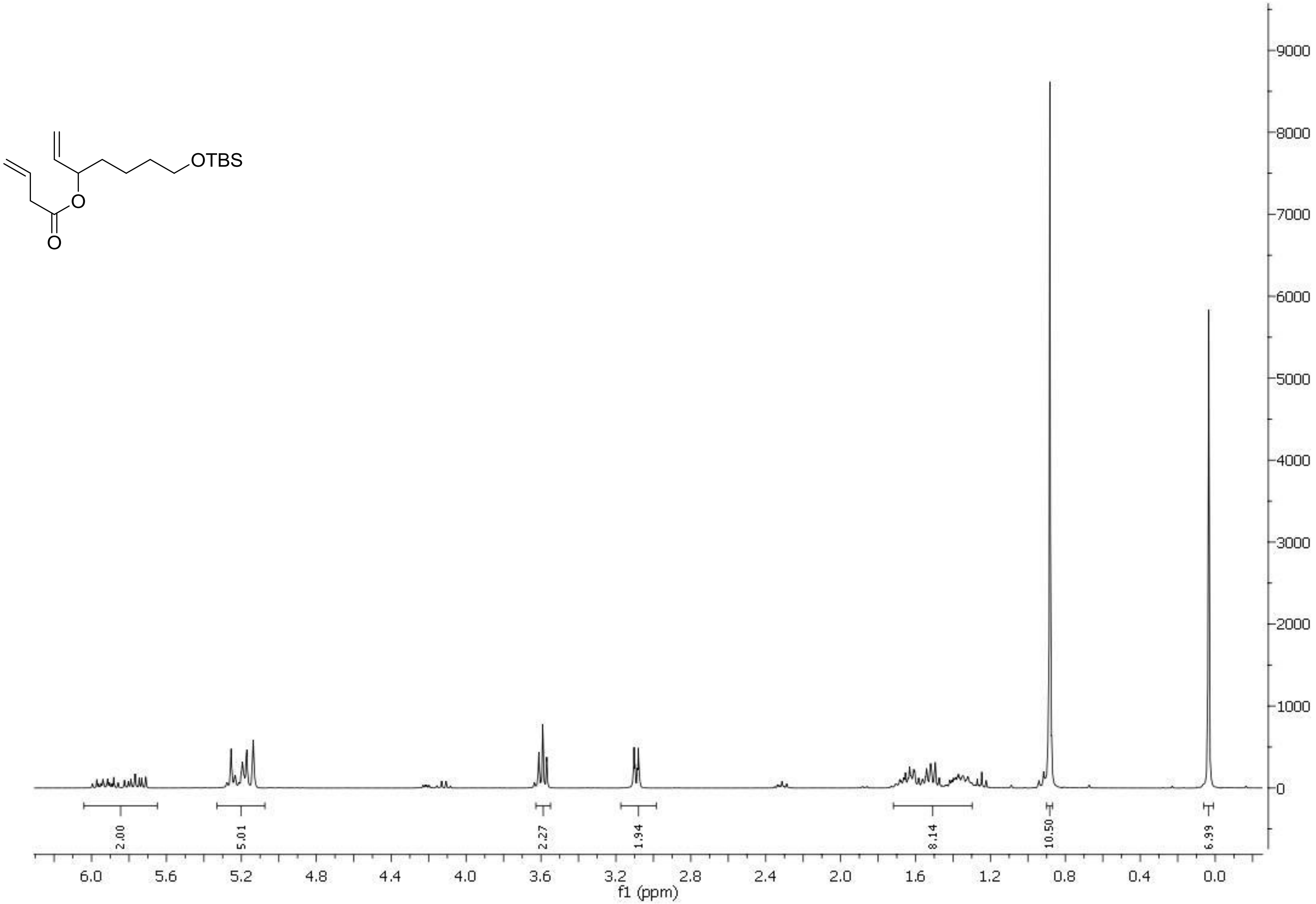

^{13}C NMR-APT (75 MHz, $CDCl_3$) of **31**

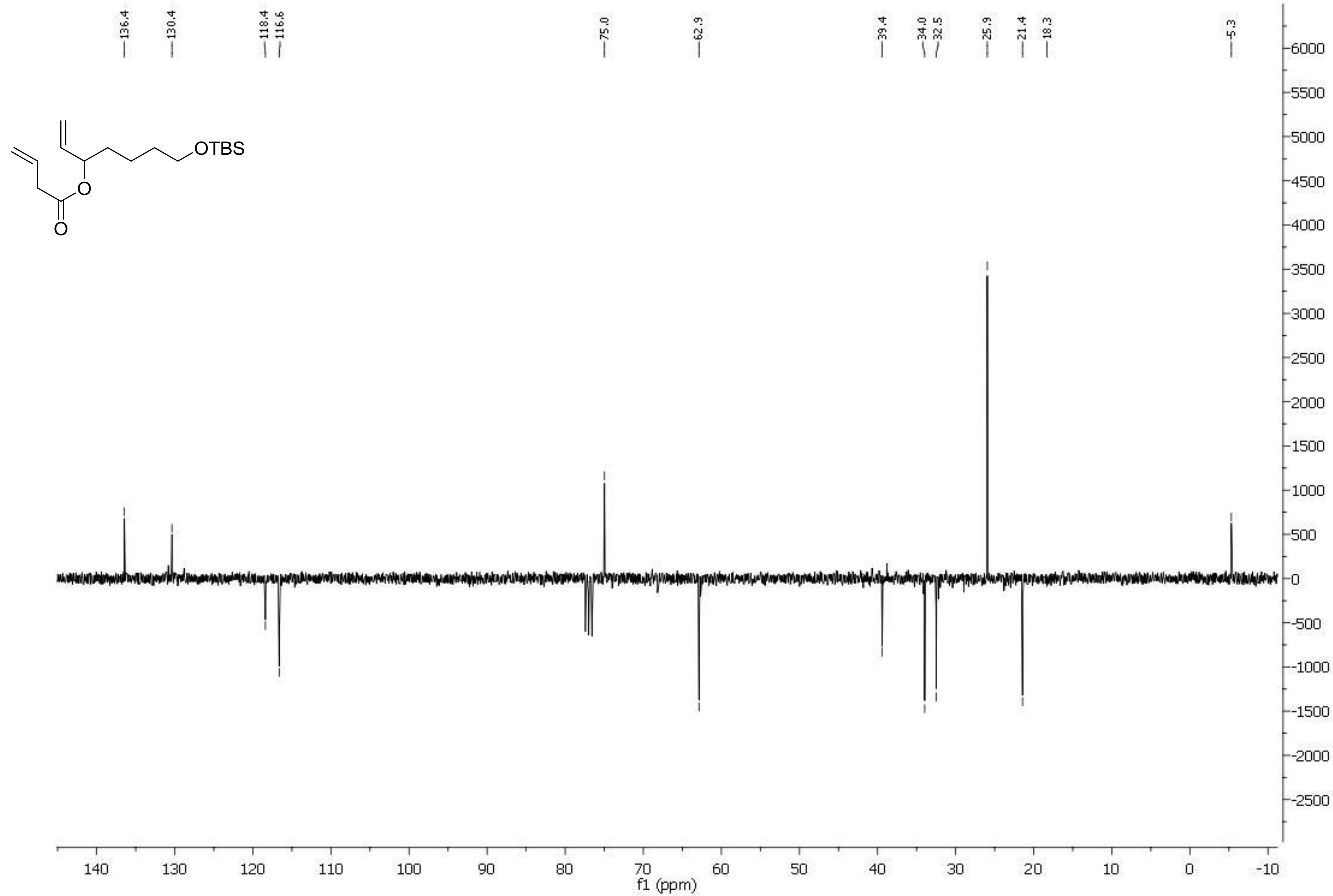

*B19 (2Z,4E)-9-(tert-Butyldimethylsilyloxy)nona-2,4-dienoic acid (**32**)*

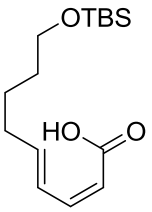

To a solution of **31** (320 mg, 1.02 mmol) in toluene (10 mL) was added Ru catalyst **B** (8.7 mg, 1 mol %) at 80 °C. The mixture was stirred for 30 min and sodium hydride (60 wt % in mineral oil, 37 mg, 1.53 mmol) was added. The mixture was stirred at 80 °C for 1 h and cooled to ambient temperature. The reaction was quenched by the addition of water and acidified with 1 M HCl. The aqueous layer was extracted three times with methyl *tert*-butyl ether (MTBE) and the combined organic layers were dried with $MgSO_4$, filtered and evaporated. The residue was purified by column chromatography on silica (eluent: hexanes/MTBE 5:1) to give **32** (195 mg, 67%) as a colourless oil: ^{1}H NMR (300 MHz, $CDCl_3$) δ 7.34 (ddd, J = 15.2, 11.4, 1.1, 1H), 6.65 (dd, J = 11.8, 11.4, 1H), 6.11 (dt, J = 15.2, 7.1, 1H), 5.57 (d, J = 11.4, 1H), 3.62 (t, J = 6.1, 2H), 2.22 (dt, J = 7.0, 6.4, 2H), 1.60-1.45 (4H), 0.89 (s, 9H), 0.05 (s, 6H); ^{13}C NMR (75 MHz, $CDCl_3$) δ 171.8 (0), 147.5 (1), 146.7 (1), 127.2 (1), 114.8 (1), 62.9 (2), 32.8 (2), 32.4 (2), 26.0 (3), 25.2 (2), 18.4 (0), −5.3 (3); IR (neat) ν 2930 (m), 2857 (m), 1691 (s), 1250 (s); MS (ESI) 176 (50), 202 (90), 285 ($[M+H]^+$, 100); HRMS (ESI) calcd. for $C_{15}H_{29}O_3Si^+$ ($[M+H]^+$) 285.1862, found 285.1886.

^{1}H NMR (300 MHz, $CDCl_3$) of **32**

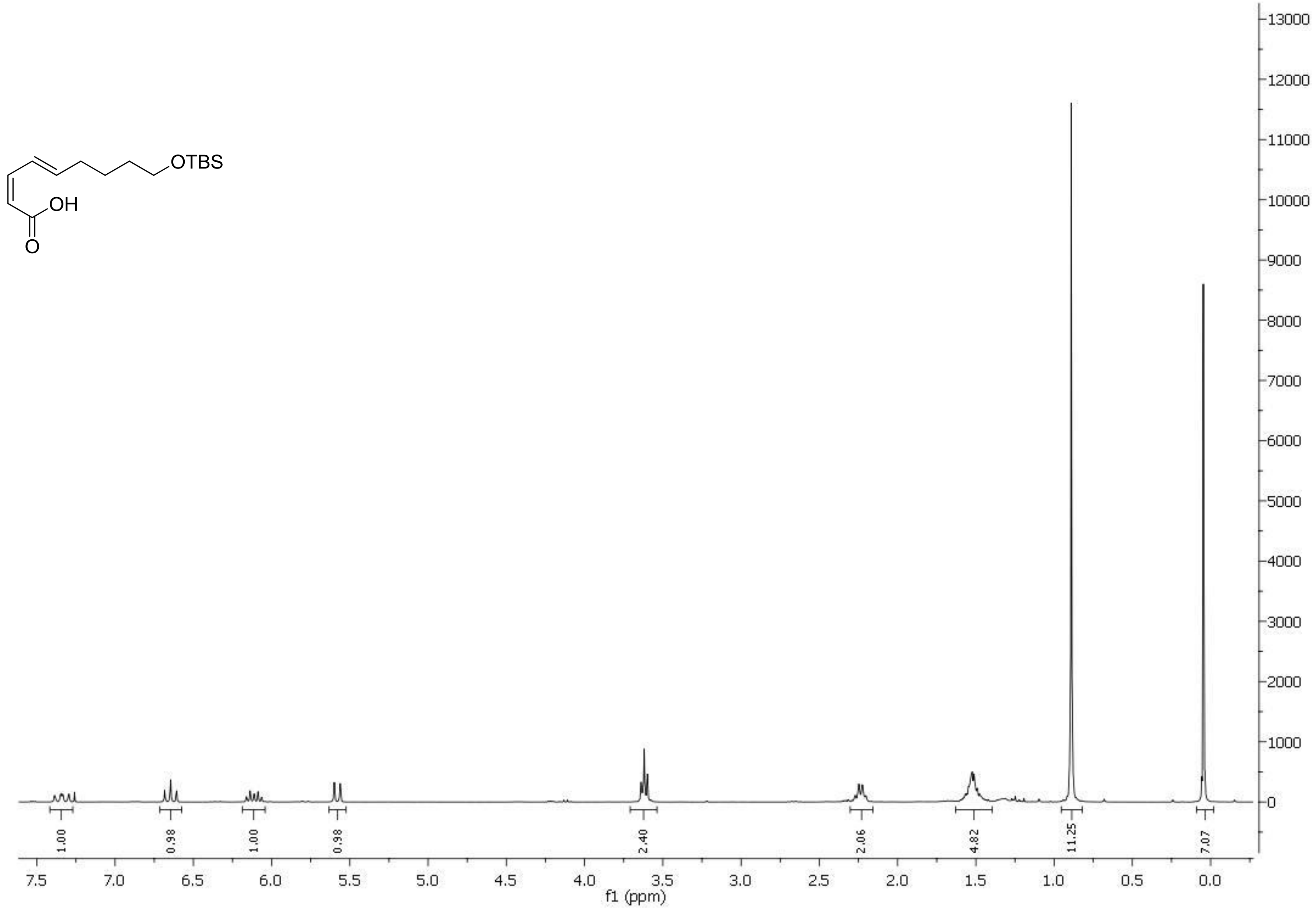

^{13}C NMR-APT (75 MHz, $CDCl_3$) of **32**

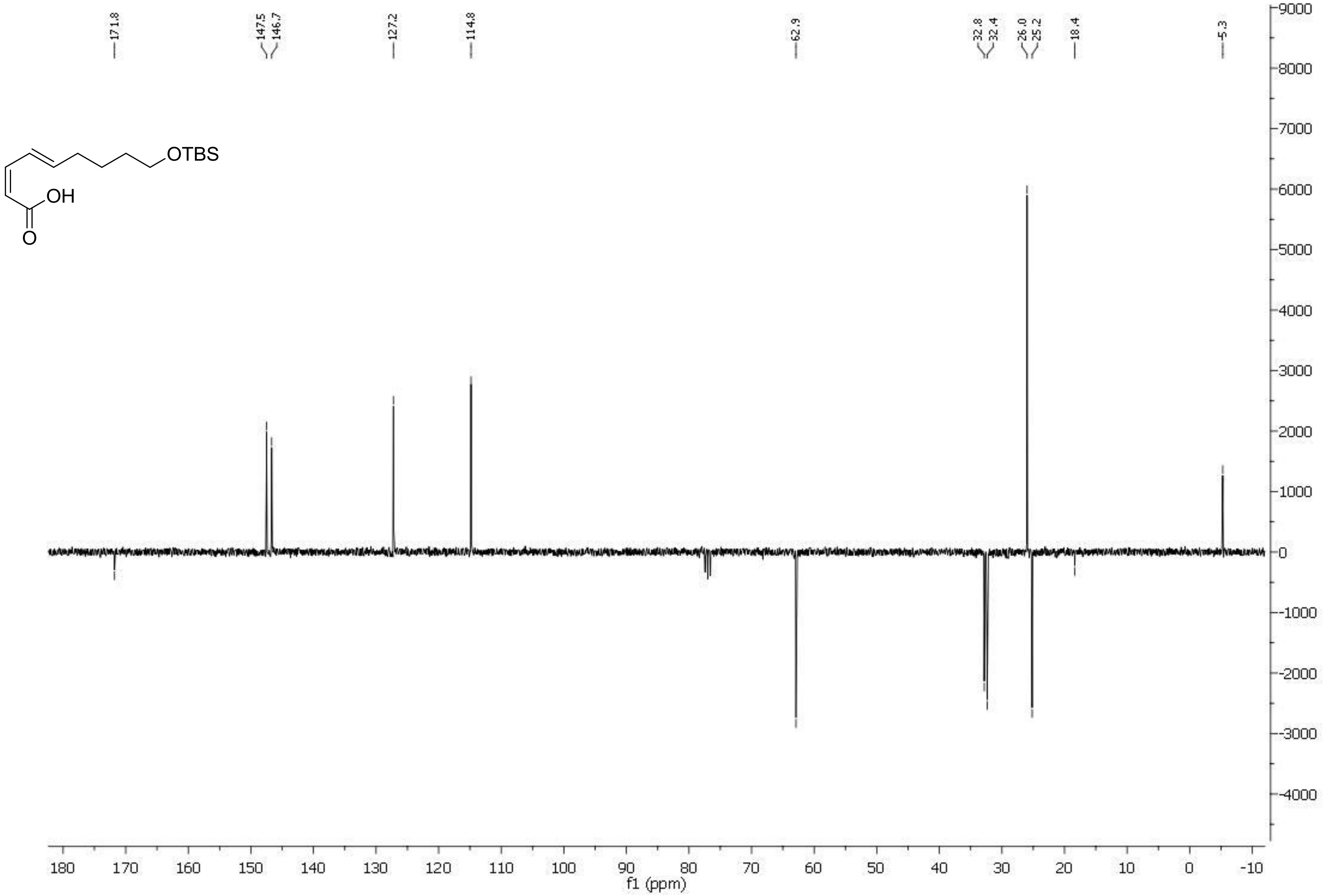

*B20 (2Z,4E)-9-Hydroxynona-2,4-dienoic acid (**33**)*

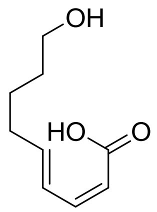

To a solution of **32** (140 mg, 0.49 mmol) in THF (5 mL) was added tetrabutylammonium fluoride (185 mg, 0.59 mmol) and the mixture was stirred for 48 h at room temperature. The reaction was quenched by the addition of water and the aqueous layer was extracted three times with MTBE. The combined organic layers were dried with $MgSO_4$, filtered and evaporated. The residue was purified by column chromatography on silica (eluent: hexanes/ethyl acetate 1:1) to give **33** (70 mg, 84%) as a colourless solid: Mp 59°C; ^{1}H NMR (300 MHz, $CDCl_3$) δ 7.32 (dd, *J* = 15.0, 11.6, 1H), 7.00-6.50 (bs, 2H), 6.63 (dd, *J* = 11.4, 11.4, 1H), 6.10 (dt, *J* = 15.3, 6.9, 1H), 5.57 (d, *J* = 11.3, 1H), 3.65 (t, *J* = 6.1, 2H), 2.23 (dt, *J* = 6.8, 6.6, 2H), 1.64-1.48 (4H); ^{13}C NMR (75 MHz, $CDCl_3$) δ 171.2 (0), 147.1 (1), 146.0 (1), 127.3 (1), 115.1 (1), 62.5 (2), 32.6 (2), 32.0 (2), 24.9 (2); IR (neat) $\tilde{\nu}$ 2934 (m), 2863 (m), 1684 (s), 1196 (s); MS (ESI) 153 (90), 171 (100), 173 ([M+H]$^+$, 8); HRMS (ESI) calcd. for $C_9H_{17}O_3^+$ ([M+H]$^+$) 173.1178, found 173.1189.

^{1}H NMR (300 MHz, $CDCl_3$) of **33**

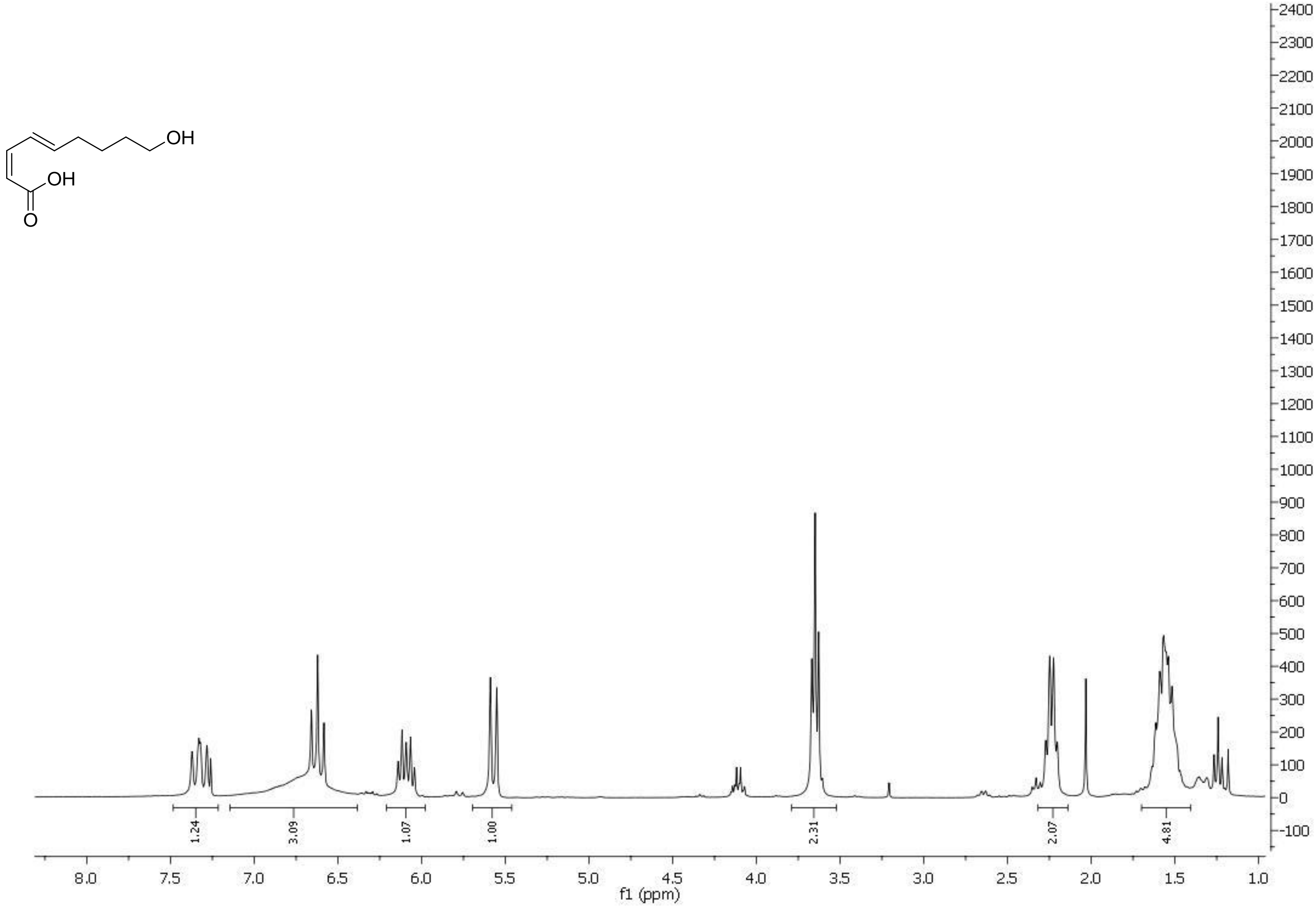

^{13}C NMR-APT (75 MHz, $CDCl_3$) of **33**

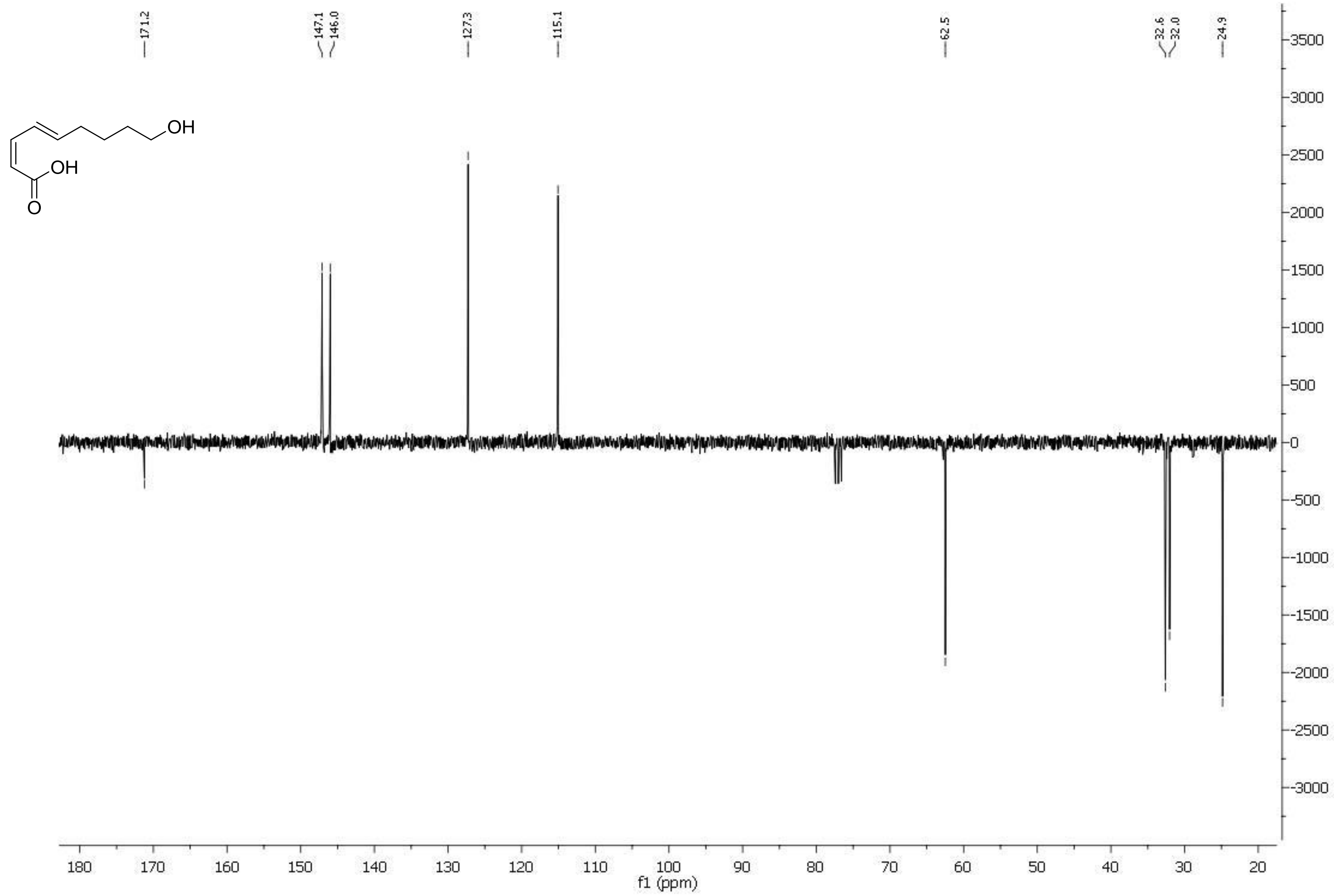

*B21 (3Z,5E)-7,8,9,10-Tetrahydro-2H-oxecin-2-one (**34**)*

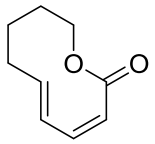

To a solution of **33** (50 mg, 0.29 mmol) and ethyl diisopropylamine (0.25 mL, 1.45 mmol) in THF (5 mL) was added 2,4,6-trichlorobenzoyl chloride (0.136 mL, 0.87 mmol) at room temperature. The solution was stirred for 2 h at room temperature. After removal of the ethyl diisopropylamine hydrochloride, the filtrate was diluted with toluene (20 mL) and added dropwise to a refluxing solution of DMAP (708 mg, 5.8 mmol) in toluene (290 mL) over a period of 1 h. The mixture was stirred at 110 °C for another 1 h and cooled to ambient temperature. After removal of the solvent the reaction mixture was diluted with MTBE (100 mL). The organic layers were washed with aqueous $NaHCO_3$ solution, aqueous NH_4Cl solution and dried with $MgSO_4$, filtered and evaporated. The residue was purified by column chromatography on silica (eluent: hexanes/MTBE 20:1) to give **34** (12 mg, 27%) as a colourless oil. ^{1}H NMR (300 MHz, $CDCl_3$) δ 6.61 (dm, J = 10.5, 1H), 5.95 (dm, J = 15.3, 1H), 5.79 (dd, J = 10.5, 1.2, 1H), 5.77 (td, J = 15.2, 1.5, 1H), 4.65 (m, 1H), 3.77 (m, 1H), 2.27 (m, 1H), 2.05-1.60 (5H); ^{13}C NMR (75 MHz, $CDCl_3$) δ 169.5, 140.9, 140.1, 126.1, 124.3, 66.0, 32.5, 30.5, 28.3; IR (neat) ν 3020 (w), 2928 (m), 2856 (w), 1710 (s), 1389 (m), 1250 (s); MS (ESI) *m/z* 135 (100), 153 ([M+H]$^+$, 80); HRMS (ESI) calcd. for $C_9H_{13}O_2^+$ ([M+H]$^+$) 153.0916, found 153.0914.

^{1}H NMR (300 MHz, $CDCl_3$) of **34**

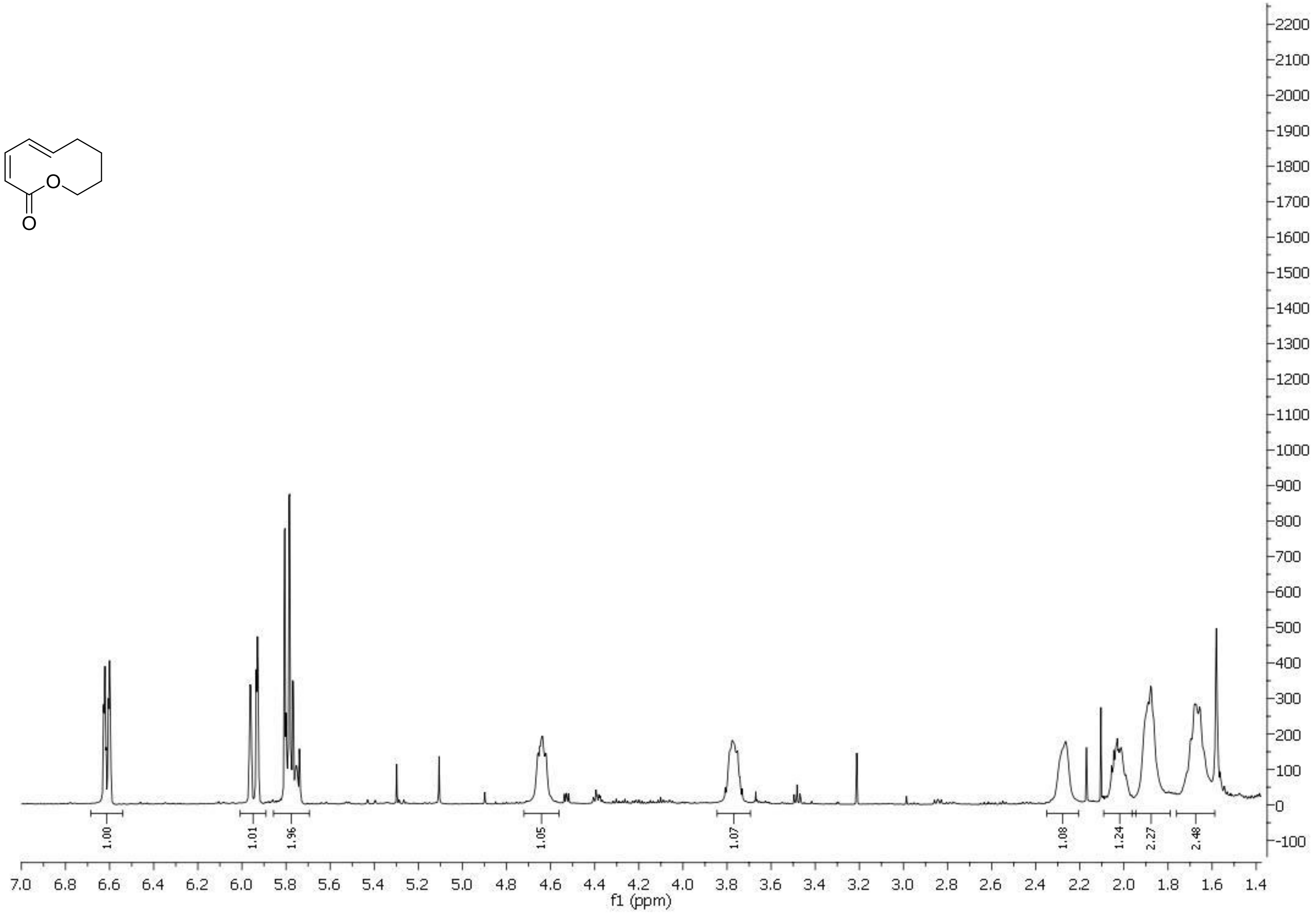

^{13}C NMR (75 MHz, $CDCl_3$) of **34**

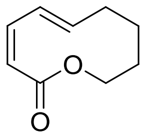

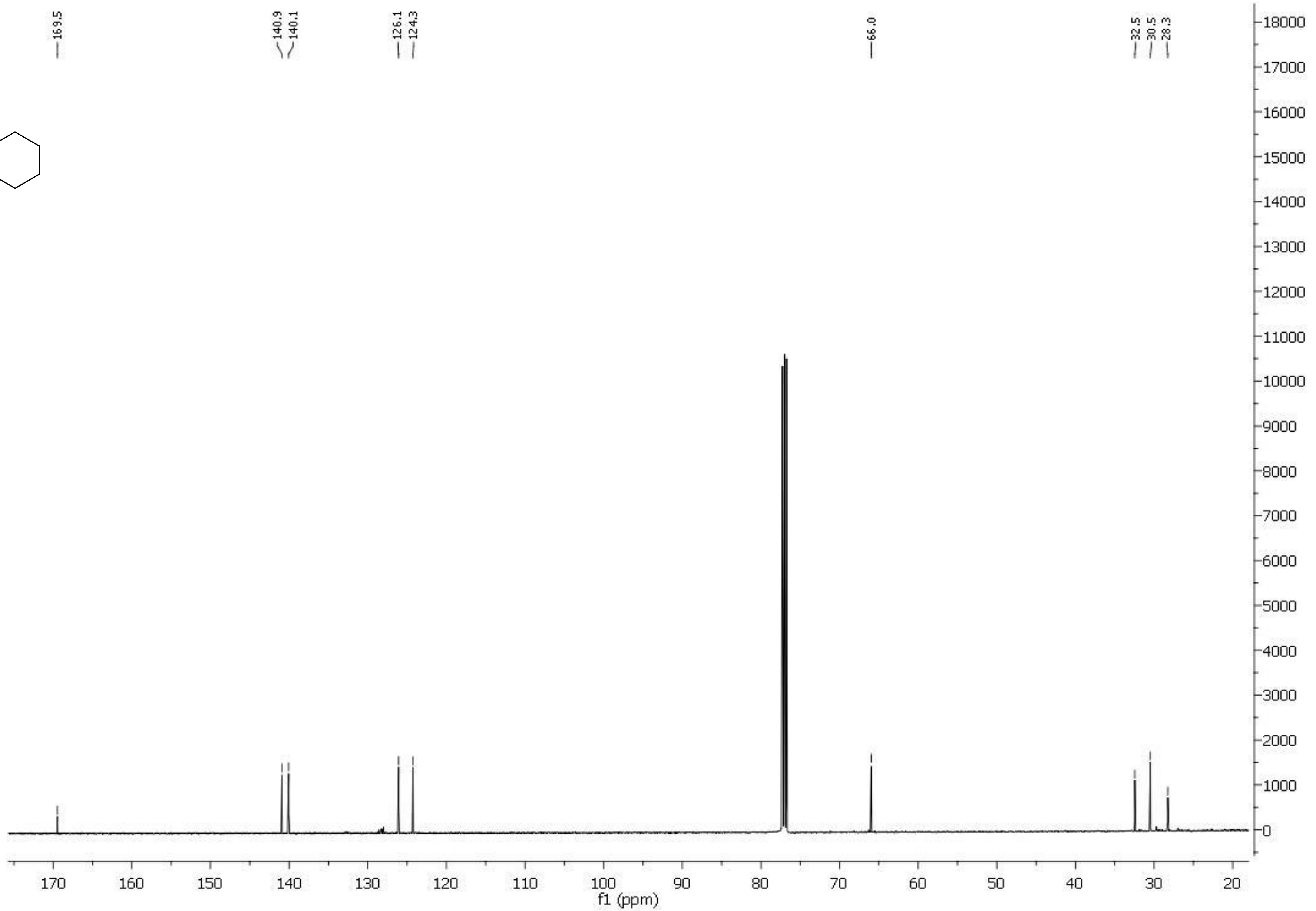

*B22 (R,3Z,5E)-10-Methyl-7,8,9,10-tetrahydro-2H-oxecin-2-one (**36**)*

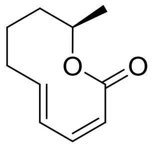

To a solution of **35** (50 mg, 0.27 mmol) and ethyl diisopropylamine (0.23 mL, 1.35 mmol) in THF (5 mL) was added 2,4,6-trichlorobenzoyl chloride (0.13 mL, 0.81 mmol) at room temperature. The solution was stirred for 2 h at room temperature. After removal of the ethyl diisopropylamine hydrochloride the filtrate was diluted with toluene (20 mL) and added dropwise to a refluxing solution of DMAP (660 mg, 5.4 mmol) in toluene (270 mL) over a period of 1 h. The mixture was stirred at 110 °C for another 1 h and cooled to ambient temperature. After removal of the solvent the reaction mixture was diluted with MTBE (100 mL). The organic layers were washed with aqueous $NaHCO_3$ solution, aqueous NH_4Cl solution and dried with $MgSO_4$, filtered and evaporated. The residue was purified by column chromatography on silica (eluent: hexanes/MTBE 5:1) to give **36** (24 mg, 53%) as a colourless oil. $[\alpha]^{25}_{D} = -112.3$ (*c* 0.18, CH_2Cl_2); ^{1}H NMR (300 MHz, $CDCl_3$) δ 6.60 (dm, J = 10.5, 1H), 5.95 (bd, J = 15.3, 1H), 5.77 (dd, J = 11.5, 1.1, 1H), 5.72 (m, 1H), 4.87 (m, 1H), 2.30 (bs, 1H), 2.05-1.53 (6H), 1.22 (d, J = 6.4, 3H); ^{13}C NMR (75 MHz, $CDCl_3$) δ 168.0 (0), 140.9 (1), 140.2 (1), 125.7 (1), 124.3 (1), 73.7 (1), 35.8 (2), 33.1 (2), 29.0 (2), 21.6 (3); IR (neat) v 2975 (w), 2930 (m), 2854 (w), 1709 (s), 1384 (w), 1260 (s); MS (ESI) *m/z* 167 ($[M+H]^+$, 100); HRMS (ESI) calcd. for $C_{10}H_{15}O_2^+$ ($[M+H]^+$) 167.1072, found 167.1073.

^{1}H NMR (300 MHz, $CDCl_3$) of **36**

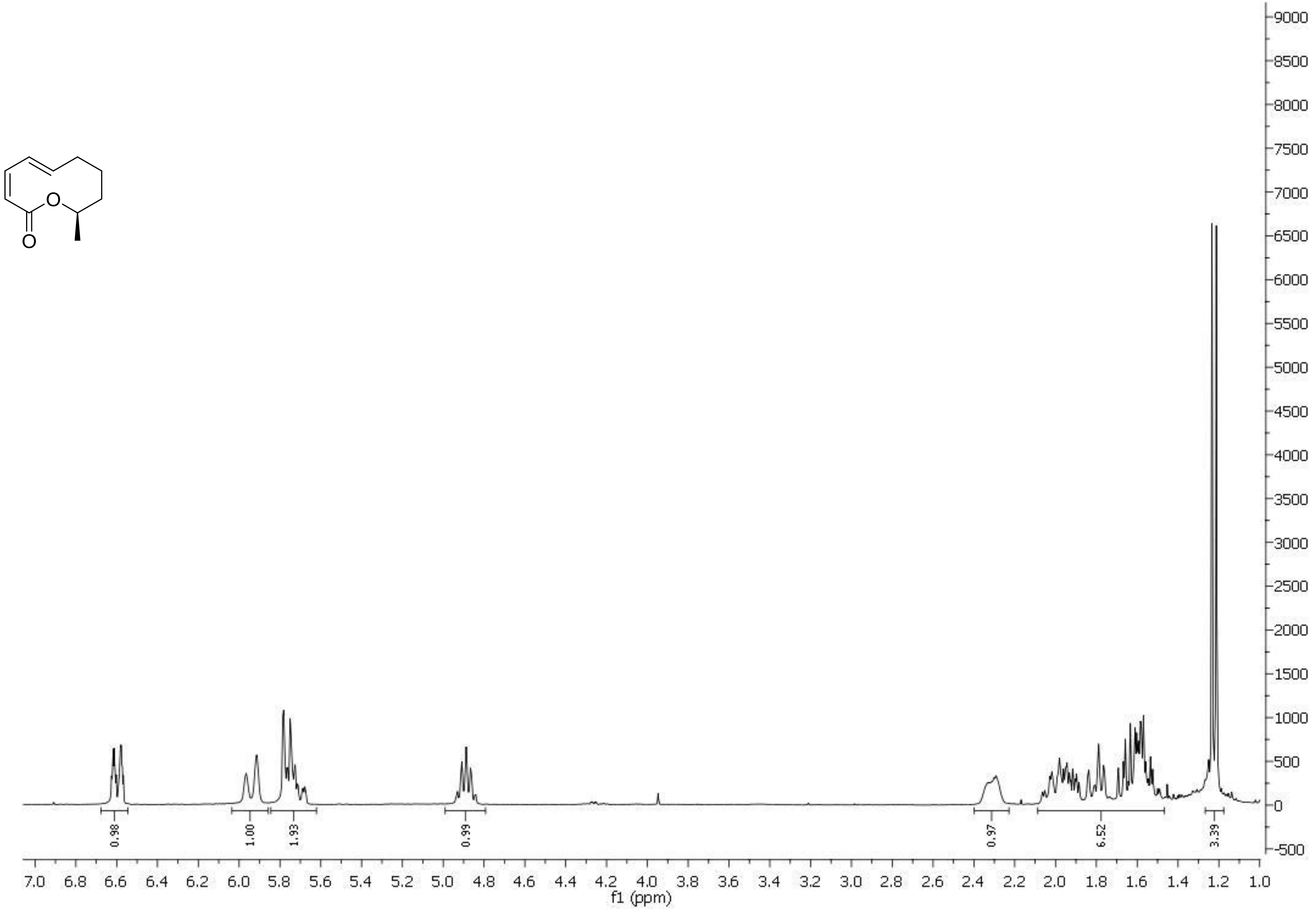

^{13}C NMR-APT (75 MHz, $CDCl_3$) of **36**

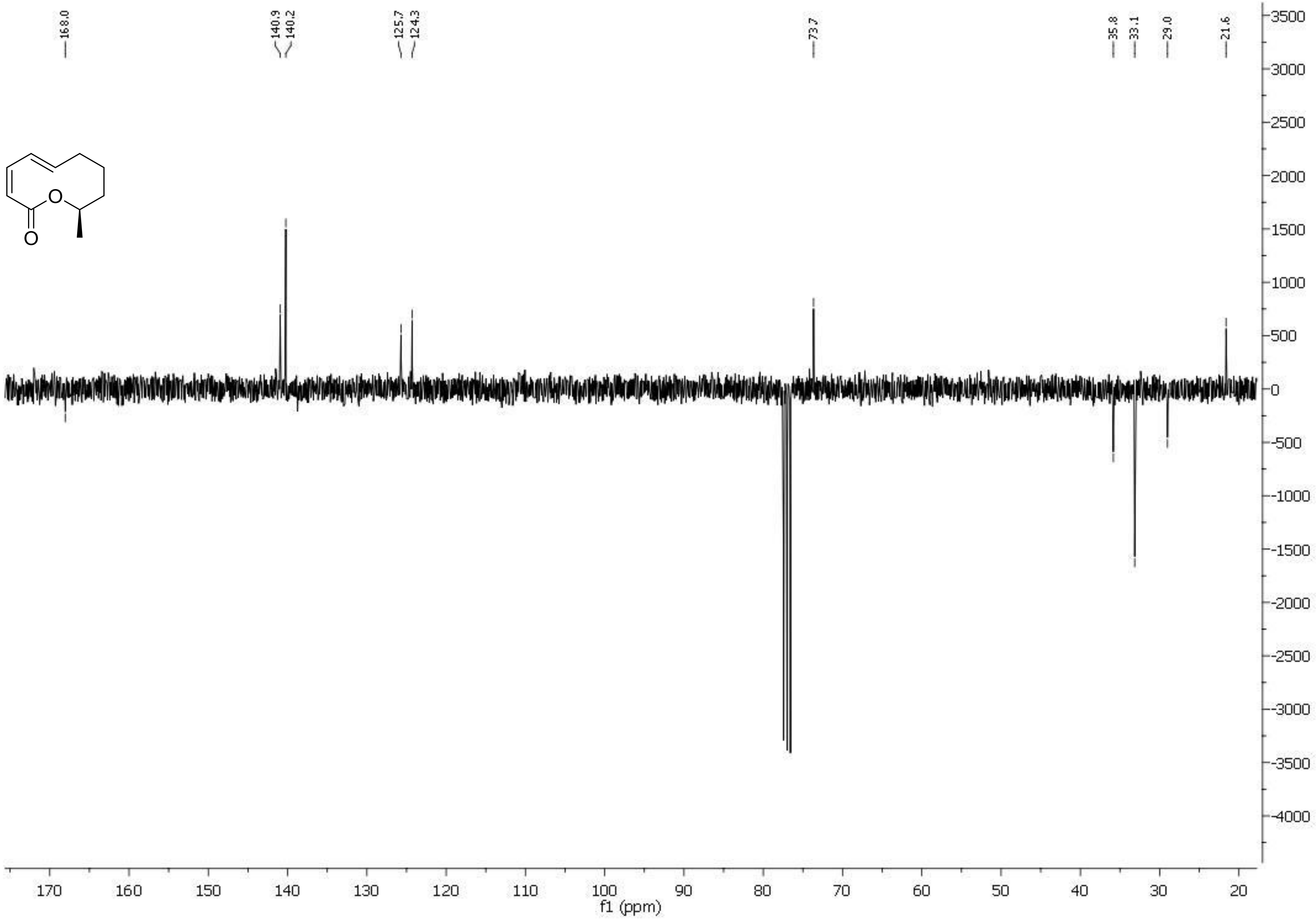

*B23 (2Z,4E,6R,9R)-6,9-Dihydroxydeca-2,4-dienoic acid (**37**)*

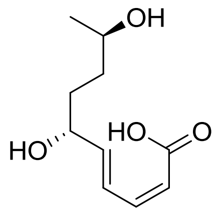

To a solution of **29** (50 mg, 0.20 mmol) in THF (2 mL) was added aqueous HCl (3 M, 1 mL) at room temperature. The mixture was stirred for 36 h and diluted with water. The aqueous layer was extracted five times with methyl *tert*-butyl ether (MTBE) and the combined organic layers were dried with $MgSO_4$, filtered and evaporated. The residue was purified by column chromatography on silica (eluent: hexanes/ethyl acetate 1:1) to give **37** (31 mg, 77%) as a colourless oil: $[\alpha]^{22}_{D}$ = −20.7 (*c* 1.05, MeOH); ^{1}H NMR (300 MHz, methanol-d_4) δ 7.47 (dd, *J* = 15.4, 11.4, 1H), 6.66 (dd, *J* = 11.4, 11.3, 1H), 6.07 (dd, *J* = 15.5, 6.2, 1H), 5.67 (d, *J* = 11.4, 1H), 4.18 (dt, *J* = 6.1, 6.0, 1H), 3.73 (m, 1H), 1.75-1.40 (4H), 1.16 (d, *J* = 6.2, 3H); ^{13}C NMR (75 MHz, methanol-d_4) δ 169.6 (0), 147.3 (1), 145.5 (1), 127.1 (1), 118.8 (1), 72.9 (1), 68.6 (1), 36.0 (2), 34.3 (2), 23.5 (3); IR (neat) $\tilde{\nu}$ 2934 (w), 1734 (s), 1646 (w), 1447 (w), 1372 (m), 1243 (s), 1034 (s); MS (ESI) *m/z* 122 (90), 147 (40), 165 (60), 183 (100), 201 ([M+H]$^+$, 50), 223 ([M+Na]$^+$, 95); HRMS (ESI) calcd. for $C_{10}H_{17}O_4^+$ ([M+H]$^+$) 201.1127, found 201.1131.

^{1}H NMR (300 MHz, MeOD-d^4) of **37**

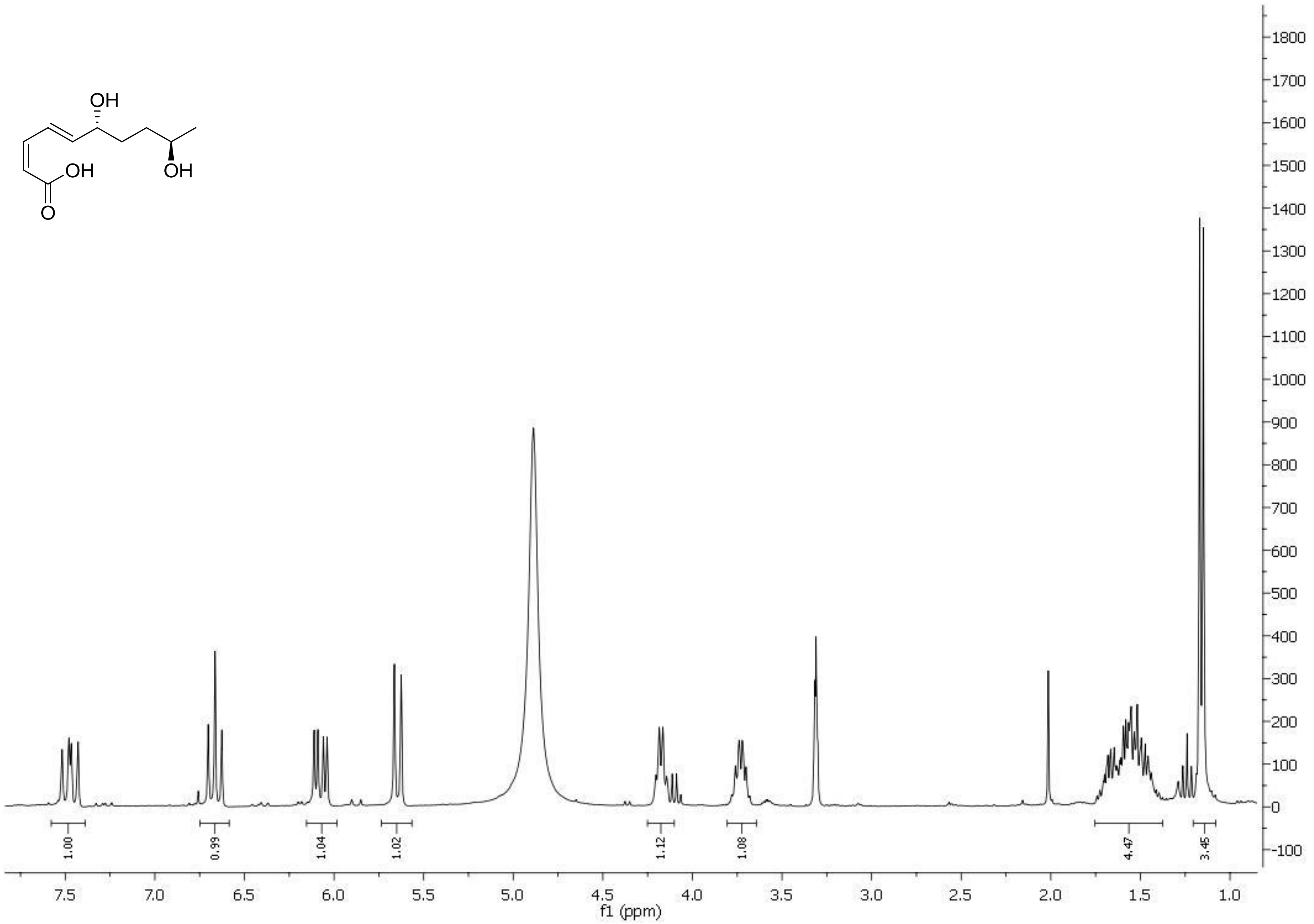

^{13}C NMR-APT (75 MHz, MeOD-d^4) of **37**

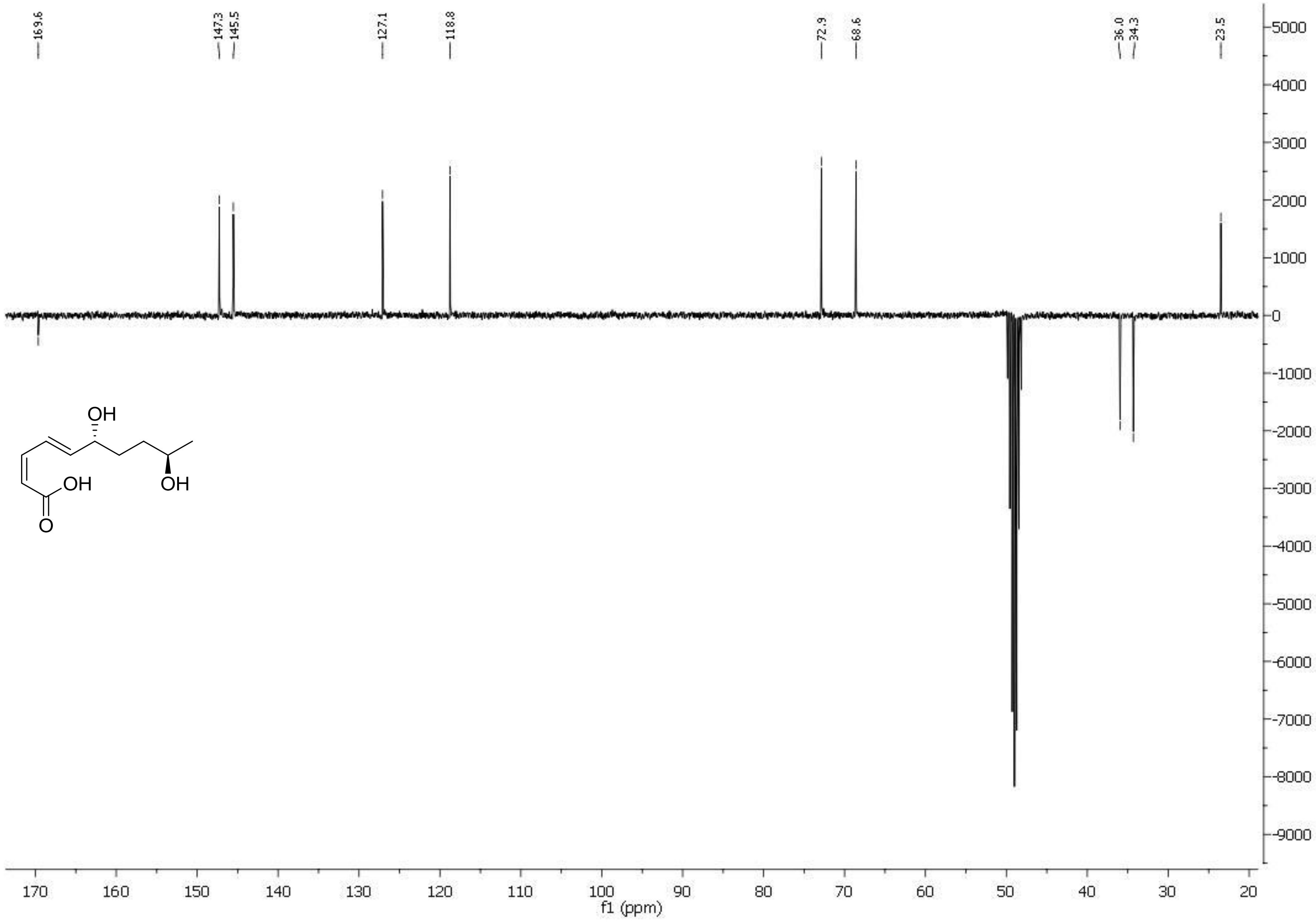

*B24 (3Z,5E,7R,10R)-7-(Methoxymethoxy)-10-methyl-7,8,9,10-tetrahydro-2H-oxecin-2-one (**38**)*[5]

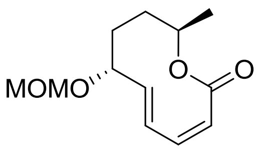

To a solution of **29** (50 mg, 0.21 mmol) and ethyl diisopropylamine (0.18 mL, 1.03 mmol) in THF (5 mL) was added 2,4,6-trichlorobenzoyl chloride (0.096 mL, 0.62 mmol) at room temperature. The solution was stirred for 2 h at room temperature. After removal of the ethyl diisopropylamine hydrochloride the filtrate was diluted with toluene (20 mL) and added dropwise to a refluxing solution of DMAP (500 mg, 4.1 mmol) in toluene (205 mL) over a period of 1 h. The mixture was stirred at 110 °C for another 1 h and cooled to ambient temperature. After removal of the solvent the reaction mixture was diluted with MTBE (100 mL). The organic layers were washed with aqueous $NaHCO_3$ solution, aqueous NH_4Cl solution and dried with $MgSO_4$, filtered and evaporated. The residue was purified by column chromatography on silica (eluent: hexanes/MTBE 5:1) to give **38** (31 mg, 67%) as a colourless oil. $[\alpha]^{24}_D = -63.3$ (*c* 0.58, CH_2Cl_2); ^1H NMR (300 MHz, $CDCl_3$) δ 6.62 (ddd, *J* = 10.6, 9.2, 1.4, 1H), 6.15 (bdd, *J* = 15.5, 1.8, 1H), 5.84 (dd, *J* = 10.7, 1.3, 1H), 5.64 (ddd, *J* = 15.5, 9.6, 1.3, 1H), 4.97 (qd, *J* = 6.6, 1.8, 1H), 4.67 (d, *J* = 6.7, 1H), 4.52 (d, *J* = 6.7, 1H), 4.14 (dt, *J* = 9.1, 4.2, 1H), 3.34 (s, 3H), 2.13-1.55 (4H), 1.21 (d, *J* = 6.5, 3H); ^{13}C NMR (75 MHz, $CDCl_3$) δ 168.2 (0), 139.6 (1), 138.6 (1), 128.3 (1), 125.5 (1), 93.9 (2), 77.0 (1), 73.0 (1), 55.3 (3), 35.0 (2), 30.4 (2), 21.2 (3); IR (neat) ν 2974 (w), 2936 (m), 1713 (s), 1384 (w), 1259 (s), 1035 (s); MS (ESI) *m/z* 165 (100), 195 (20), 227 ($[M+H]^+$, 15); HRMS (ESI) calcd. for $C_{12}H_{19}O_4^+$ ($[M+H]^+$) 227.1283, found 227.1299.

[5] Compound **38** has previously been synthesized in the course of Sabitha's stagonolide E synthesis. Analytical data reported by these authors match those found by us well, apart from the value for the specific rotation, which was reported to be $[\alpha]^{25}_D = +47.4$ (*c* 0.8, $CHCl_3$). See: Sabitha, G.; Padmaja, P.; Reddy, P. N.; Jadav, S. S.; Yadav, J. S. *Tetrahedron Lett.* **2010**, *51*, 6166-6168.

^{1}H NMR (300 MHz, $CDCl_3$) of **38**

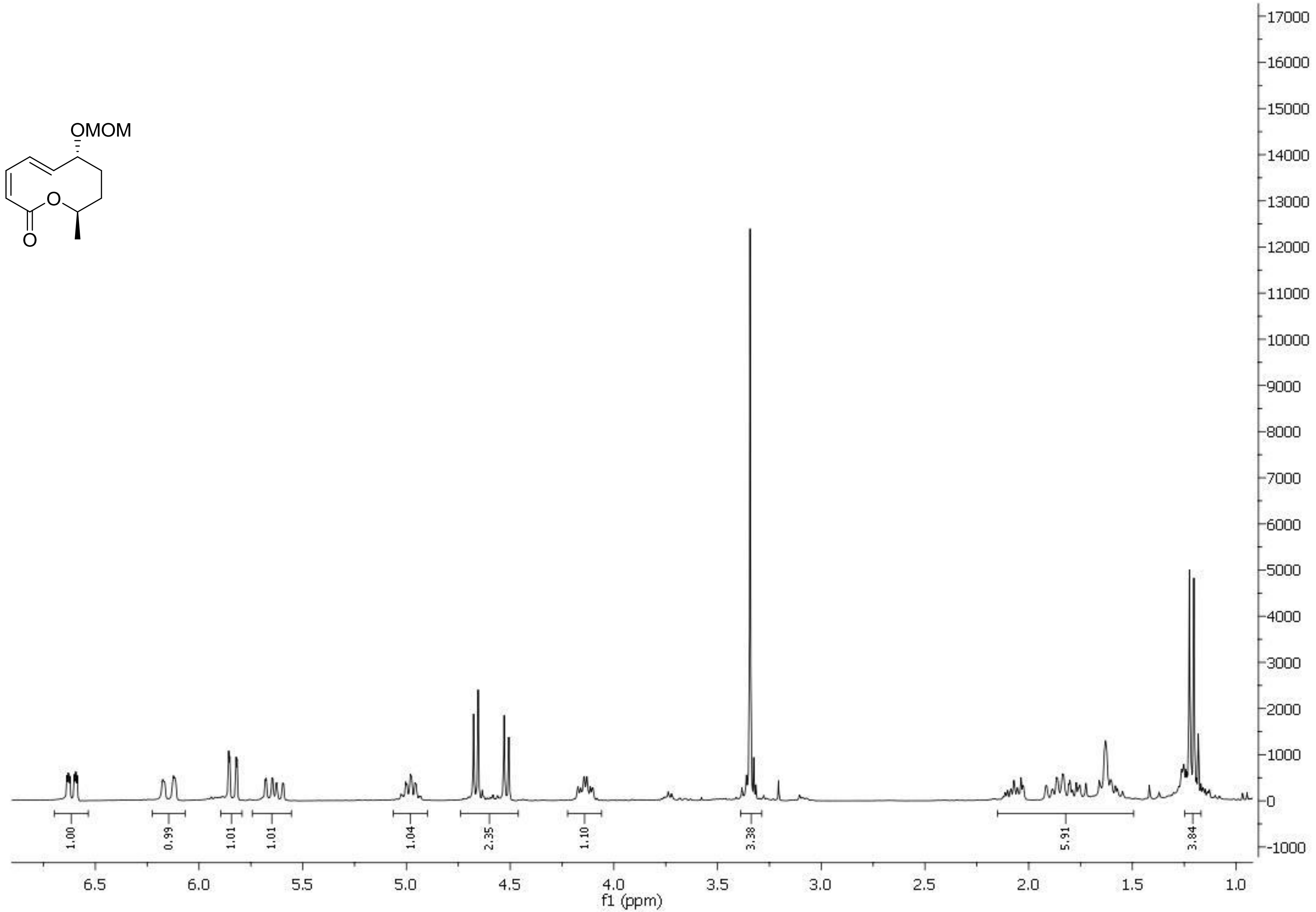

^{13}C NMR-APT (75 MHz, $CDCl_3$) of **38**

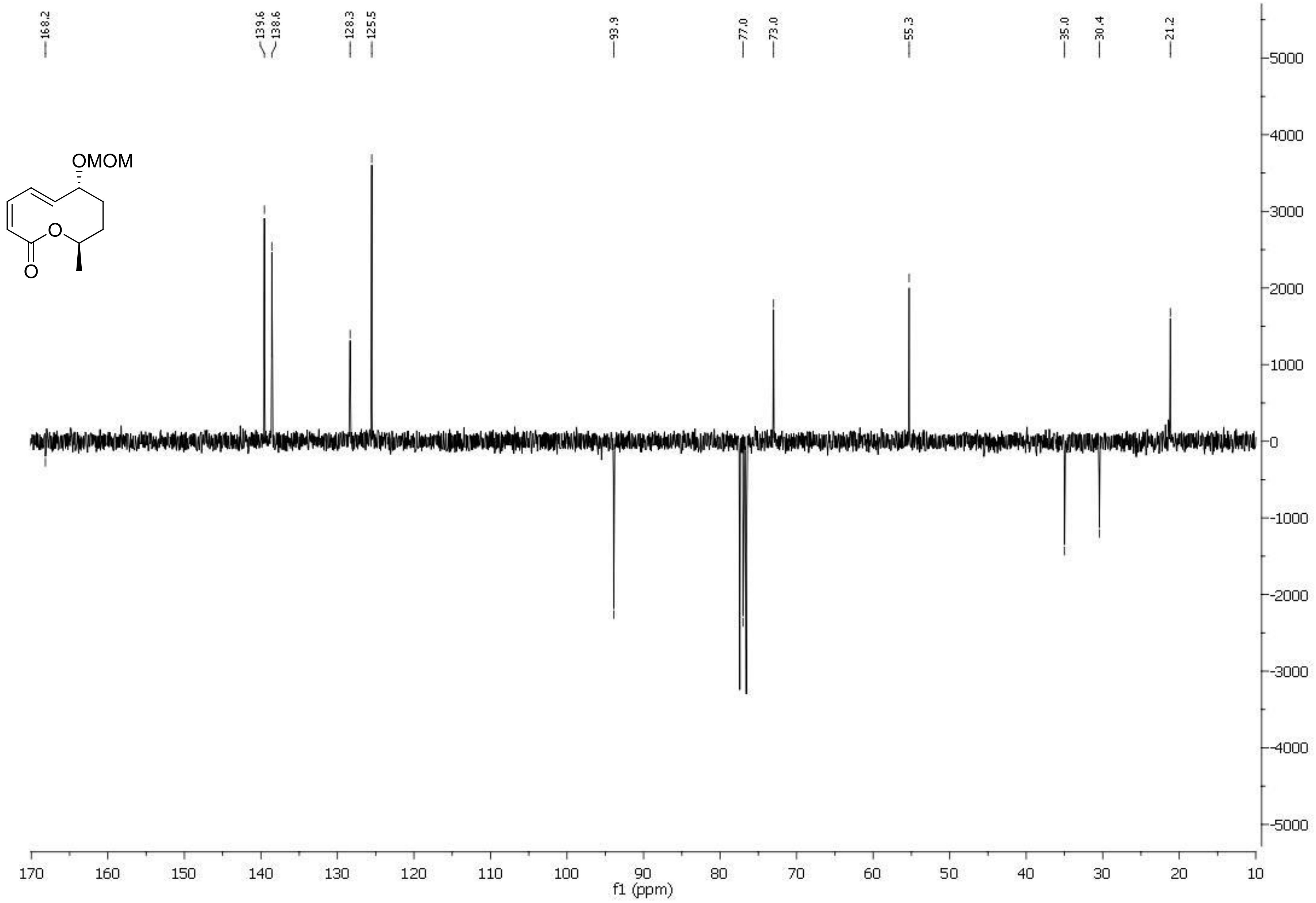

B25 Stagonolide E

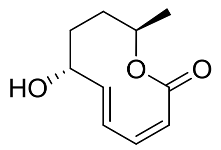

To a solution of **38** (25 mg, 0.11 mmol) in dichlormethane (4 mL) was added trifluoroacetic acid (1 mL) at room temperature. The mixture was stirred for 1.5 h, diluted with MTBE (10 mL) and neutralized with aqueous $NaHCO_3$ solution. The aqueous layer was extracted three times with methyl *tert*-butyl ether (MTBE) and the combined organic layers were dried with $MgSO_4$, filtered and evaporated. The residue was purified by column chromatography on silica (eluent: hexanes/MTBE 1:1) to give stagonolide E (18 mg, 90%) as a colourless oil: $[\alpha]^{24}_{D} = -177.3$ (*c* 0.65, CH_2Cl_2); ^1H NMR (300 MHz, $CDCl_3$) δ 6.61 (ddd, *J* = 10.6, 9.3, 1.4, 1H), 6.10 (bd, *J* = 15.4, 1H), 5.84 (dd, *J* = 10.7, 1.2, 1H), 5.72 (ddd, *J* = 15.3, 9.4, 1.1, 1H), 4.97 (dq, *J* = 7.0, 6.6, 1H), 4.22 (ddd, *J* = 9.0, 9.0, 3.9, 1H), 2.07 (ddd, *J* = 13.8, 8.8, 3.8, 1H), 1.90-1.55 (3H), 1.21 (d, *J* = 6.5, 3H); ^{13}C NMR (75 MHz, $CDCl_3$) δ 168.2 (0), 140.2 (1), 139.6 (1), 126.5 (1), 125.6 (1), 73.6 (1), 73.2 (1), 37.3 (2), 30.3 (2), 21.3 (3); IR (neat) ν 3405 (m), 2977 (w), 2933 (m), 1705 (s), 1385 (w), 1259 (s), 1055 (s); MS (ESI) *m/z* 122 (100), 167 (60), 183 ($[M+H]^+$, 10), HRMS (ESI) calcd. for $C_{10}H_{15}O_3^+$ ($[M+H]^+$) 183.1021, found 183.1024.

^{1}H NMR (300 MHz, $CDCl_3$)

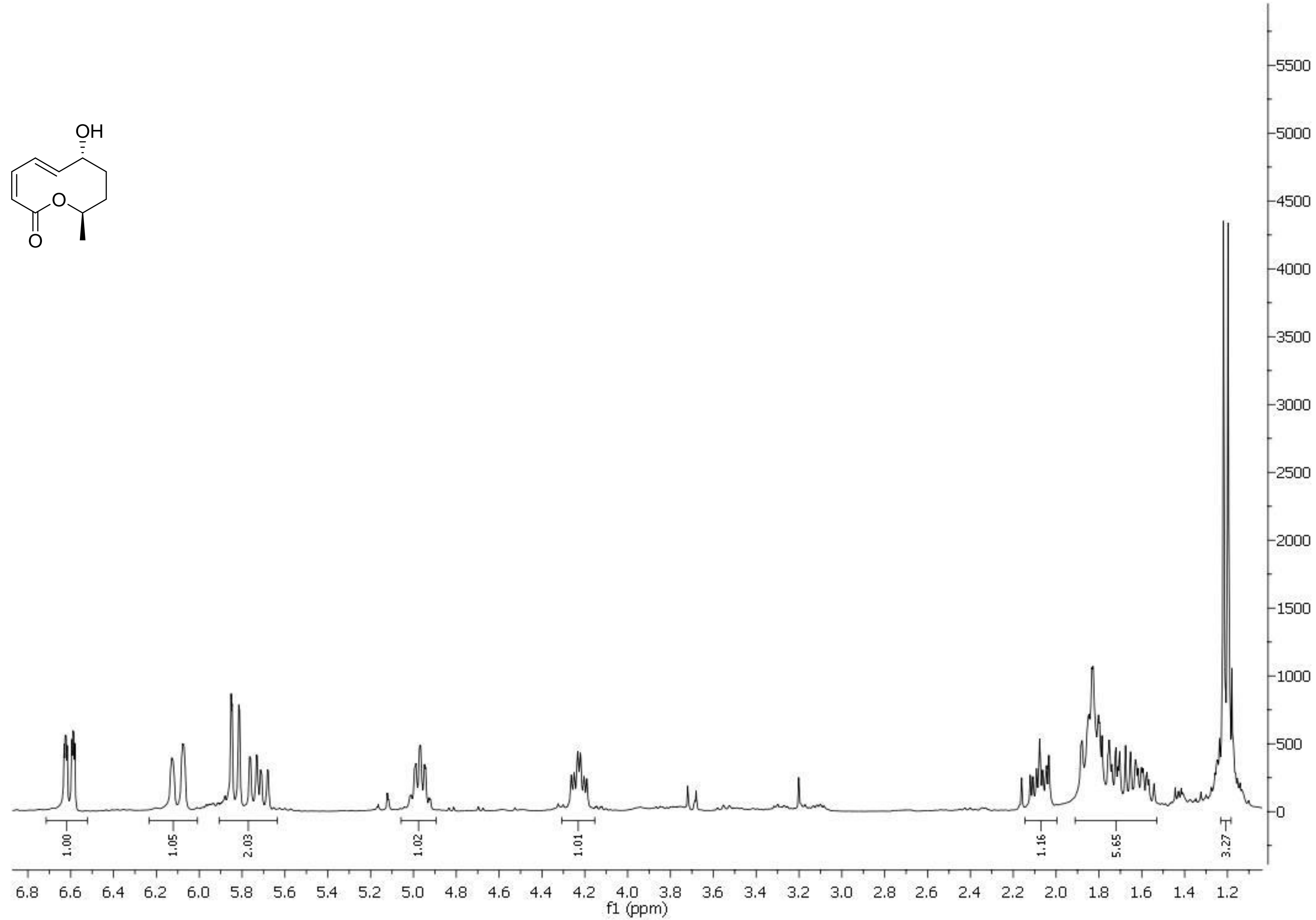

^{13}C NMR-APT (75 MHz, $CDCl_3$)

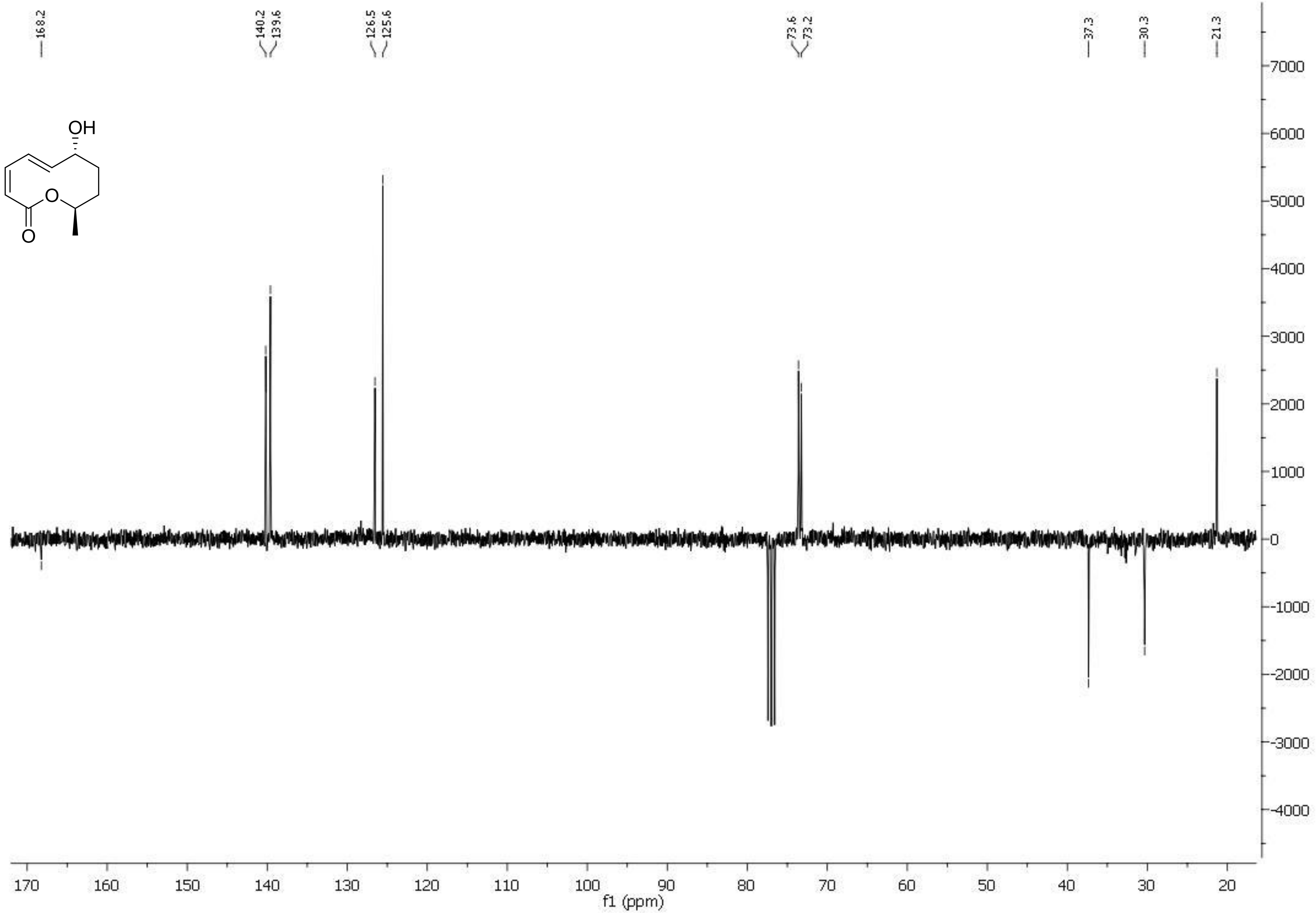

B26 Curvulide A

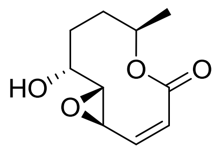

To a solution of $Ti(OiPr)_4$ (0.051 mL 0.17 mmol) in CH_2Cl_2 (5 mL) was added D-(−)-diethyl tartrate (0.034 mL, 0.20 mmol) at 0 °C. The mixture was stirred at this temperature for 15 min, and stagonolide E (25 mg, 0.14 mmol) was added. Stirring at 0 °C was continued for another 15 min, and *tert*-butyl hydroperoxide (5.5 M solution in decane, 0.076 mL, 0.42 mmol) was added and the reaction mixture was stirred for 24 h at 0 °C. After this time, $FeSO_4$ (0.2 g) was dissolved in an aqueous solution of tartaric acid (15 wt %, 10 mL) and added to the reaction mixture at 0 °C. The mixture was allowed to warm to room temperature and filtered through a pad of celite, extracted three times with MTBE, and washed with brine. The organic layers were dried with $MgSO_4$, filtered and evaporated. The residue was purified by column chromatography on silica (eluent: hexanes/MTBE 1:1) to give curvulide A (16 mg, 58%) as a colourless oil: $[\alpha]^{21}_{D}$ = +117.0 (*c* 0.18, MeOH); ^1H NMR (300 MHz, acetone-d^6) δ 6.52 (dd, J = 11.1, 1.0, 1H), 5.90 (dd, J = 11.1, 2.0, 1H), 4.72 (m, 1H), 3.63 (dt, J = 2.0, 1.6, 1H), 3.21 (tm, J = 8.1, 1H), 2.60 (dd, J = 8.2, 2.2, 1H), 2.00-1.90 (2H), 1.75-1.65 (2H), 1.26 (d, J = 6.2, 3H); ^{13}C NMR (75 MHz, acetone-d^6) δ 165.3, 143.0, 125.6, 76.1, 74.8, 65.0, 56.7, 34.1, 33.3, 21.1; IR (neat) v 3427 (m), 2930 (m), 2853 (w), 1717 (s), 1383 (m), 1270 (s); MS (ESI) *m/z* 181 (70), 199 ([M+H]$^+$, 100), 221 ([M+Na]$^+$, 15), HRMS (ESI) calcd. for $C_{10}H_{15}O_4^+$ ([M+H]$^+$) 199.0970, found 199.0973.

^{1}H NMR (300 MHz, acetone-d^6)

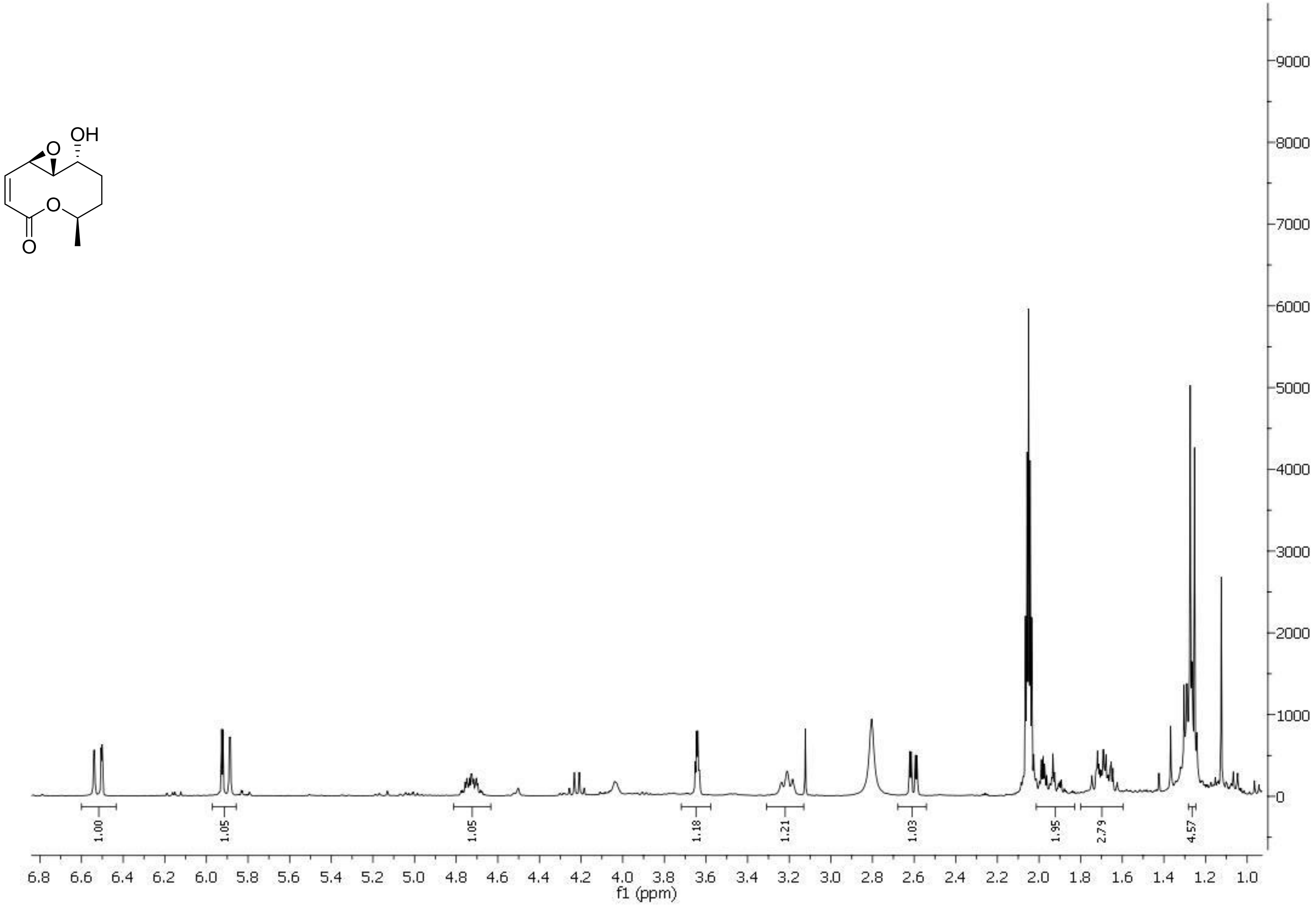

^{13}C NMR (75 MHz, acetone-d^6)

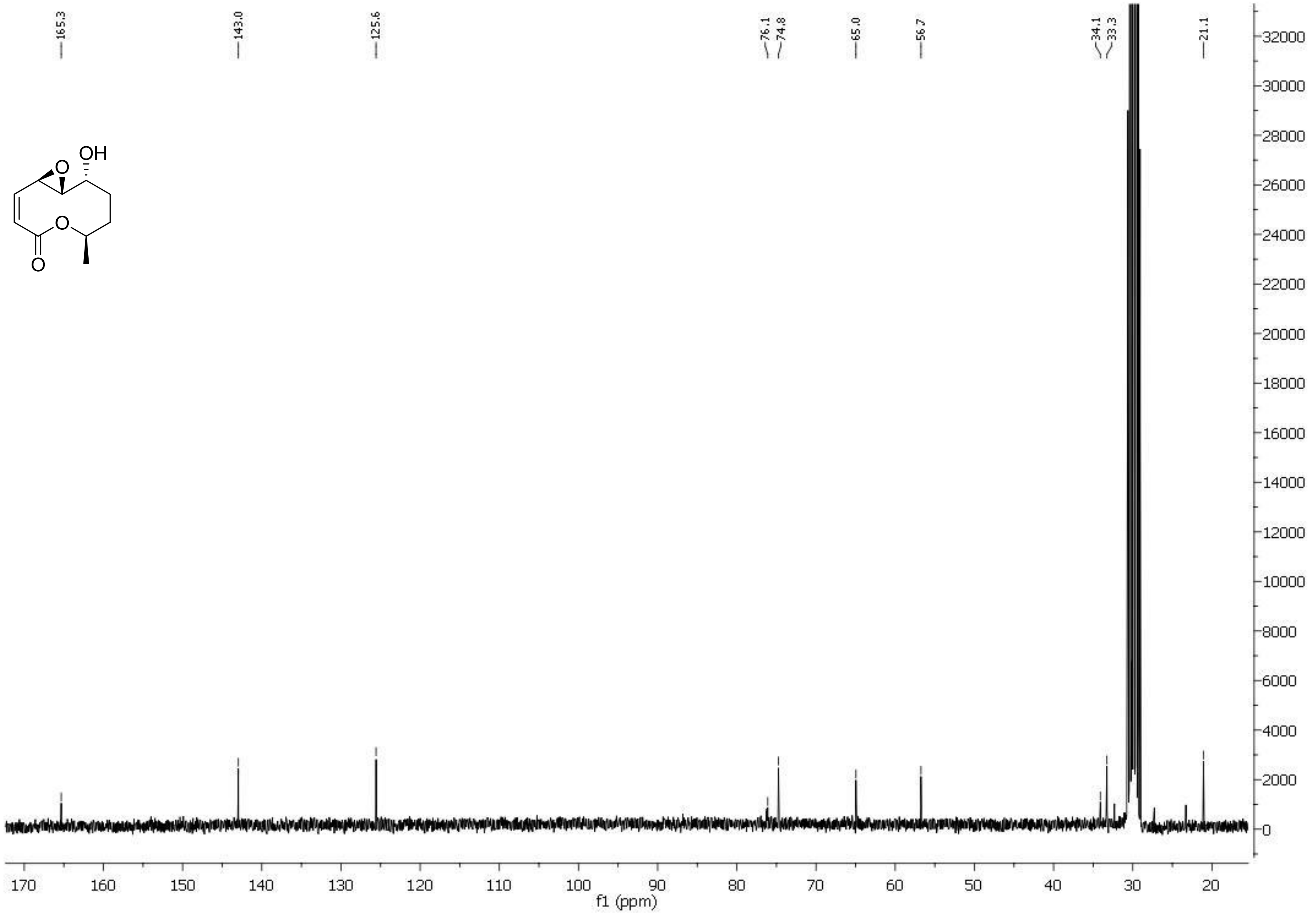

Supplement: File 1 — Experimental procedures, characterization data and copies of 1H and 13C NMR spectra. [file Beilstein_J_Org_Chem-09-2544-s001.pdf]
